# Candidemia in intensive care units over nine years at a large Italian university hospital: Comparison with other wards

**DOI:** 10.1371/journal.pone.0252165

**Published:** 2021-05-26

**Authors:** Sara Mazzanti, Lucia Brescini, Gianluca Morroni, Elena Orsetti, Antonella Pocognoli, Abele Donati, Elisabetta Cerutti, Christopher Munch, Roberto Montalti, Francesco Barchiesi

**Affiliations:** 1 Dipartimento di Scienze Biomediche e Sanità Pubblica Università Politecnica delle Marche, Azienda Ospedaliera Universitaria Ospedali Riuniti Umberto I°- Lancisi-Salesi, Ancona, Italy; 2 Clinica Malattie Infettive, Azienda Ospedaliera Universitaria Ospedali Riuniti Umberto I°- Lancisi-Salesi, Ancona, Italy; 3 Malattie Infettive, Ospedale Murri, Fermo, Italy; 4 Laboratorio di Microbiologia, Azienda Ospedaliera Universitaria Ospedali Riuniti Umberto I°- Lancisi-Salesi, Ancona, Italy; 5 Clinica di Anestesia e Rianimazione, Azienda Ospedaliera Universitaria Ospedali Riuniti Umberto I°- Lancisi-Salesi, Ancona, Italy; 6 Anestesia e Rianimazione, Azienda Ospedaliera Universitaria Ospedali Riuniti Umberto I°- Lancisi-Salesi, Ancona, Italy; 7 Anestesia e Rianimazione Cardiochirurgica, Azienda Ospedaliera Universitaria Ospedali Riuniti Umberto I°- Lancisi-Salesi, Ancona, Italy; 8 Unità di Chirurgia Epato-Bilio-Pancreatica, Mininvasiva e Robotica, Dipartimento di Sanità Pubblica, Università Federico II, Napoli, Italy; 9 Malattie Infettive, Azienda Ospedaliera Ospedali Riuniti Marche Nord, Pesaro, Italy; University of Palermo, ITALY

## Abstract

**Purpose:**

Candidemia is an alarming problem in critically ill patients including those admitted in intensive care units (ICUs). We aimed to describe the clinical and microbiological characteristics of bloodstream infections (BSIs) due to *Candida* spp. in patients admitted to ICUs of an italian tertiary referral university hospital over nine years.

**Methods:**

A retrospective observational study of all cases of candidemia in adult patients was carried out from January 1, 2010 to December 31, 2018 at a 980-bedded University Hospital in Ancona, Italy, counting five ICUs. The incidence, demographics, clinical and microbiologic characteristics, therapeutic approaches and outcomes of ICU-patients with candidemia were collected. Non-ICU patients with candidemia hospitalized during the same time period were considered for comparison purposes. Early (7 days from the occurrence of the episode of *Candida* BSI) and late (30 days) mortality rates were calculated.

**Results:**

During the study period, 188/505 (36%) episodes of candidemia occurred in ICU patients. Cumulative incidence was 9.9/1000 ICU admission and it showed to be stable over time. *Candida albicans* accounted for 52% of the cases, followed by *C*. *parapsilosis* (24%), and *C*. *glabrata* (14%). There was not a significant difference in species distribution between ICU and non-ICU patients. With the exception of isolates of *C*. *tropicalis* which showed to be fluconazole resistant in 25% of the cases, resistance to antifungals was not of concern in our patients. Early and late mortality rates, were 19% and 41% respectively, the latter being significantly higher than that observed in non-ICU patients. At multivariate analysis, factors associated with increased risk of death were septic shock, acute kidney failure, pulmonary embolism and lack of antifungal therapy. The type of antifungal therapy did not influence the outcome. Mortality did not increased significantly over time.

**Conclusion:**

Neither cumulative incidence nor crude mortality of candidemia in ICU patients increased over time at our institution. However, mortality rate remained high and significantly associated with specific host-related factors in the majority of cases.

## Introduction

Invasive fungal diseases and in particular candidemia, are an alarming problem in critically ill patients including those admitted in intensive care units (ICUs). The incidence of candidemia in these patients ranges between 2 and up to 10 cases per 1000 ICU admissions, with a crude mortality rate reaching 60% [1–4].

Although *Candida albicans* accounts for the majority of *Candida* infections, an increasing number of cases due to *Candida* spp. other than *C*. *albicans* are often reported in some series or in specific geographic areas [5–9]. Additionally, the implementation of antifungals for empiric or preemptive strategies in critically ill patients, has led to the emergence of *Candida* spp. that are resistant to azoles and/or echinocandins [10–13]. However, most of these studies focused on specific populations or they were conducted for a limited period of time [14, 15].

The primary objective of the present study was to estimate the cumulative incidence of bloodstream infections (BSIs) due to *Candida* spp. in patients admitted to ICUs of an italian tertiary referral university hospital over nine years. The secondary objectives included the analysis of demographics, clinical and microbiologic characteristics, therapeutic approaches and outcome of candidemia in these patients. Non-ICU patients with candidemia hospitalized during the same time period were also considered for comparison purposes.

## Materials and methods

### Hospital setting and study design

The setting was a 980-bedded University Hospital in Ancona, Italy, including five ICUs (one each of cardiologic unit, post-cardiac surgery unit, general and post-solid organ transplant surgery unit, medical unit and high care unit), 11 medical and 11 surgical wards. A retrospective observational study of all cases of candidemia in adult patients (>16 years old) was carried out from January 1, 2010 to December 31, 2018. The Institutional Review Board of the “Azienda Ospedaliero-Universitaria Ospeadali Riuniti Umberto I-Lancisi-Salesi” granted retrospective access to the data without need for individual informed consent. The consent was not given since the data were analyzed anonymously.

### Case definition

A case of candidemia was defined as isolation of *Candida* species from blood culture in a patient with temporally related clinical signs and symptoms of infection.

Episodes were considered to be distinct if they were caused by different *Candida* spp. or they occurred at least 30 days apart with elapsing resolution of clinical features of infection and at least one negative blood culture.

### Data collection

All *Candida* BSIs were identified through the microbiological laboratory database. Demographic, clinical risk factors, and laboratory data were collected from the patient’s medical records. A catheter-related candidemia was defined according to the guidelines of the Infectious Disease Society of America (IDSA) [16]. Appropriate antifungal therapy was considered when an appropriate drug (based on subsequent in vitro susceptibility testing results) with adequate dosage was started within 72 hours from the first blood culture performed. Adequate dosage of an antifungal agent was defined according to IDSA guidelines [17, 18]. Mortality was calculated after seven days (early mortality) and 30 days (late mortality) from the occurrence of the episode of *Candida* BSI.

### Microbiological methods

*Candida* species were isolated from blood samples using BacT/ALERT (bioMérieux) and identified with the MALDI-TOF Biotyper (Bruker Daltonics, Germany).

Antifungal susceptibility testing was performed for fluconazole, caspofungin and amphotericin B using the SensititreYeastOne colorimetric plate (Trek Diagnostic System) and MIC results were interpreted according to the latest species-specific clinical breakpoints as established by the Clinical and Laboratory Standards Institute (CLSI) [19]. The three drugs were selected since each of them is the representative of a specific class.

### Statistical analysis

Cumulative incidence of candidemia were calculated per 1000 hospital admissions using annual hospital activity. Linear regression analysis was utilized to define the correlations between years and incidence of candidemia and mortality. Categorical variables were expressed as absolute numbers and their relative frequencies; continuous variables were expressed as median and interquartile range (IQR).

Categorical variables were compared by the χ2 or Fisher exact test, while continuous variables were evaluated by the Student *t* test (for normally distributed variables) or the Mann-Whitney *U* test (for nonnormally distributed variables). Variables which reached a statistical significance (*p* < 0.05) at univariate analysis were analyzed by multivariate logistic regression analysis to identify independent risk factors for either early mortality or late mortality. Results were expressed as hazard ratio (HR) and 95% CI. All statistical analyses were performed using the statistical package SPSS for Windows v. 20 (SPSS Inc., Chicago, IL, USA). A *p* value <0.05 was considered to represent statistical significance and all statistical tests were two-tailed.

## Results

### Incidence of candidemia

**Fig 1:**
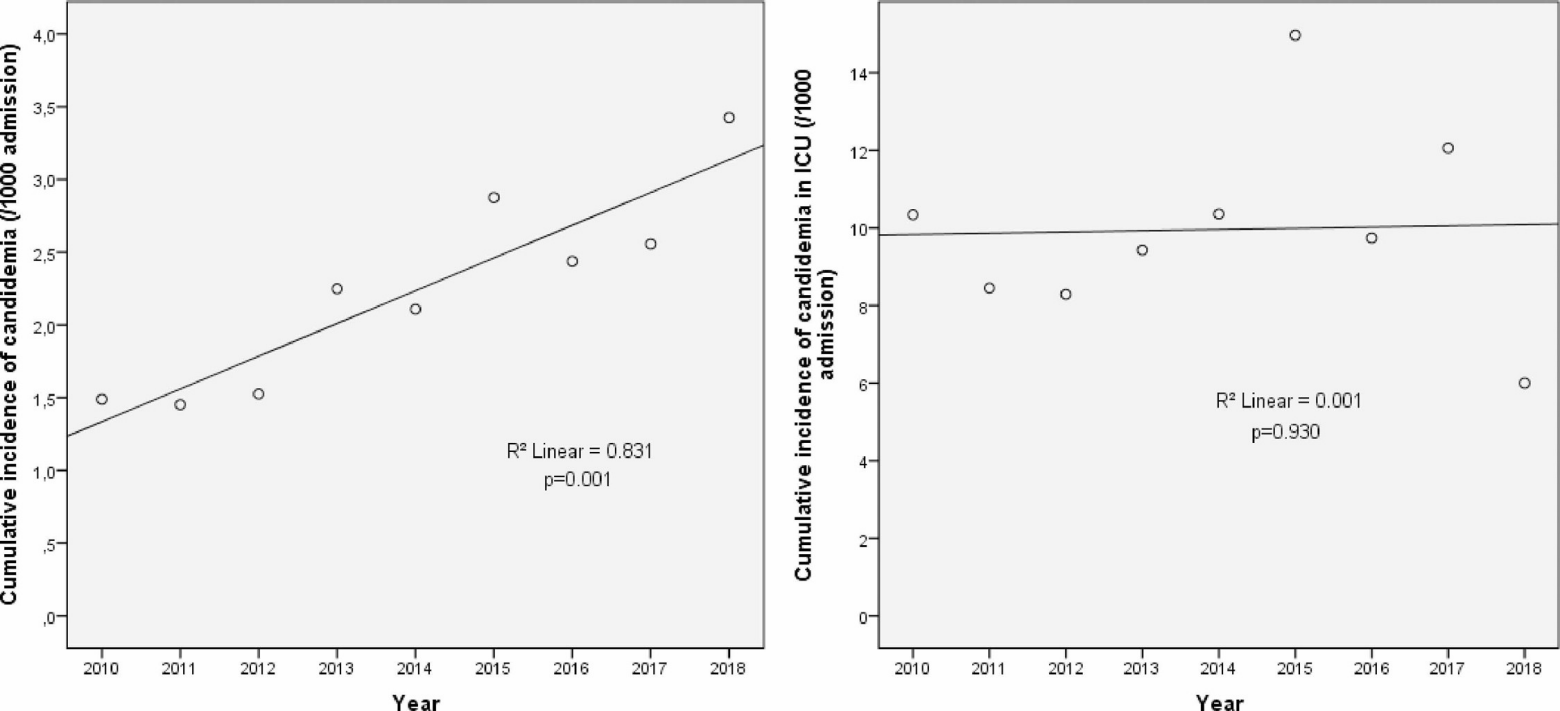
Cumulative incidence of candidemia over nine years in the overall population (A) and in ICU patients (B).

During the study period, 505 episodes of candidemia from 470 patients were diagnosed. There were 188 episodes (36%) occurring in 176 ICU patients and 317 episodes (63%) occurring in 294 non-ICU patients. The overall cumulative incidence (mean) of candidemia was 2.2/1000 hospital admission with a significant increase over time (Fig 1A). The cumulative incidence of candidemia in ICU patients was 9.9/1000 hospital admission and it was stable over time (Fig 1B).

### Demographic and clinical characteristics of the study population

Demographic and clinical characteristics of ICU patients with candidemia compared to non-ICU patients are provided in Table 1. The majority were male (62%), with a median age of 71 years. Chronic comorbidities were frequent. The majority of patients were suffering from cardiovascular diseases (71%), followed by neurological diseases (27%), diabetes mellitus (26%), and chronic renal failure (22%). Surgery within the past 30 days was found in 51% of the patients with cardiovascular surgery the most frequent (32%).

**Table 1:** Demographic and clinical characteristics of patients included in the study.

Characteristics	All patients	ICU	non-ICU	*P* ^a^	
	n = 505	n = 188	n = 317		
Male sex, *n (%)*	313 (62)	116 (62)	197 (62)	0.921	
Age, median (IQR) ^b^	70 (60–77)	71 (60–77)	70 (60–77)	0.355	
Chronic pulmonary diseases, *n (%)* ^c^	74 (15)	40 (21)	34 (11)	0.001	
Hematological malignancy, *n (%)*	28 (6)	6 (3)	22 (7)	0.075	
Cardiovascular diseases, *n (%)* ^d^	284 (56)	134 (71)	150 (47)	<0.0001	
Neurological diseases, *n (%)* ^e^	122 (24)	51 (27)	71 (22)	0.230	
Gastrointestinal diseases, *n (%)* ^f^	132 (26)	39 (21)	93 (29)	0.034	
Diabetes mellitus, *n (%)*	101 (20)	48 (26)	53 (17)	0.017	
Chronic renal failure, *n (%)*	81 (16)	42 (22)	39 (12)	0.003	
Solid tumors, *n (%)*	164 (33)	37 (20)	124 (40)	<0.0001	0.006
Chronic hepatic disease, *n (%)*	52 (10)	12 (6)	40 (13)	0.026	
Solid organ transplant, *n (%)*	16 (3)	1 (1)	15 (5)	0.009	
Any Surgery *n (%)*	229 (45)	96 (51)	133 (43)	0.047	
Gastrointestinal surgery, *n (%)*	60 (12)	19 (10)	41 (13)	0.342	
Cardiovascular surgery, *n (%)*	89 (18)	61 (32)	28 (9)	<0.0001	<0.001
Neurosurgery, *n (%)*	41 (8)	13 (7)	28 (9)	0.446	
Charlson’s score, median (IQR) ^b^	5 (4–7)	5 (4–7)	5 (7–7)	0.222	
Central venous catheter, *n (%)*	448 (89)	177 (94)	271 (86)	0.003	0.033
Central venous catheter-related BSIs, *n (%)* ^g^	332 (66)	119 (64)	213 (68)	0.336	
Early central venous catheter removal, *n (%)* ^h^	103 (20)	36 (19)	67 (21)	0.592	
Other devices, *n (%)* ^i^	440 (87)	175 (93)	265 (84)	0.002	
Previous invasive procedures (<72 hours), *n (%)* ^j^	146 (29)	79 (42)	67 (21)	<0.0001	0.005
Parenteral nutrition, *n (%)*	318 (63)	114 (61)	204 (64)	0.446	
Renal Replacement Therapy, *n (%)*	44 (9)	29 (16)	15 (5)	<0.001	
Steroid therapy, *n (%)*	145 (29)	52 (28)	93 (29)	0.714	
Immunosuppressive therapy, *n (%)* ^k^	49 (10)	4 (2)	45 (14)	<0.0001	0.01
Neutropenia, *n (%)*	14 (3)	2 (1)	12 (4)	0.073	
Pneumonia, *n (%)*	164 (33)	72 (39)	32 (29)	0.028	
Septic shock, *n (%)*	75 (15)	45 (24)	30 (10)	<0.001	
Acute kidney failure, *n (%)*	46 (9)	30 (16)	16 (5)	<0.001	0.012
*Candida* species					
*Candida albicans*, *n (%)*	256 (51)	98 (52)	158 (50)	0.077	
*Candida parapsilosis*, *n (%)*	129 (26)	45 (24)	84 (27)		
*Candida tropicalis*, *n (%)*	44 (9)	13 (7)	31 (10)		
*Candida glabrata*, *n (%)*	50 (10)	26 (14)	24 (8)		
Other *Candida* species, *n (%)* ^l^	26 (5)	6 (3)	20 (6)		
Appropriate antifungal therapy, *n (%)* ^m^	271 (54)	91 (49)	180 (57)	0.077	
Primary antifungal therapy					
Azoles, *n (%)*	221 (45)	79 (42)	142 (45)	0.544	
Echinocandins, *n (%)*	153 (30)	48 (25)	105 (33)	0.073	
No treatment, *n (%)*	123 (25)	59 (32)	64 (20)	0.003	0.001
7-days mortality, *n(%)*	71 (14)	35 (19)	36 (11)	0.022	
30-days mortality, *n(%)*	150 (30)	77 (41)	73 (23)	<0.001	0.014

*a* In the first column, p-value is referred to the univariate analysis performed by Chi-Square test or Fisher Exact Test when expected frequencies were less than five, in the second column p-value is referred to logistic regression analysis.

*b* IQR, Interquartile range

*c* Chronic pulmonary diseases include asthma, chronic bronchitis, emphysema and lung fibrosis

*d* Cardiovascular diseases include heart failure, ischemic heart disease, endocarditis and arrhythmia

*e* Neurological diseases include Parkinson’s disease, Alzheimer’s disease and paralysis

*f* Gastrointestinal diseases include Crohn’s disease, ulcerative colitis, chronic pancreatitis and gallbladder stones

*g* A catheter-related candidemia was defined according to the guidelines of the infectious diseases society of America (IDSA: Mermel et al. [16])

*h* Early central venous catheter removal was considered occurring within 48 h from blood cultures drawing

*i* Other devices include urinary catheter, surgical drainage, cutaneous gastrostomy and tracheostomy tube

*j* Previous invasive procedures include endoscopy and positioning of any device

*k* Immunosuppressive therapy include calcineurin inhibitors and monoclonal antibodies

*l* Other *Candida* species included *Candida guilliermondii* (n = 10), *Candida krusei* (n = 5), *Candida lusitaniae* (n = 4), *Candida dubliniensis* (n = 2), and one isolate each of *Candida kefyr*, *Candida norvegensis*, *Candida pelliculosa*, *Candida rugosa* and *Candida utilis*

m Appropriate antifungal therapy was considered when the appropriate drug with adequate dosage was started within 72 hours the first blood culture performed

The median Charlson’s score was 5. Central venous catheter (CVC) was present in 94% of the cases and 64% of the analyzed catheters were found to be the source of the *Candida* BSI. Early CVC removal occurred in 19% of the episodes. In 93% of cases there were additional devices. The most common acute complications during candidemia were pneumonia (39%), septic shock (24%) and acute kidney failure (16%). When comparing ICU *vs* non-ICU patients, the former had more frequently undergone cardiovascular surgery, inserted CVCs at the onset of candidemia, previous invasive procedures, and acute kidney failure. On the contrary, solid tumors and immunosuppressive therapy were significantly less common in ICU patients.

### Microbiology and antifungal susceptibility testing results

*Candida albicans* accounted for 52% of the cases observed in ICU patients, followed by *C*. *parapsilosis* (24%), and *C*. *glabrata* (14%) (Table 1). The latter species was more common in ICU patients while *C*. *tropicalis* and uncommon *Candida* species were more frequently isolated in non-ICU patients. Still, there was not a statistically significant difference in species distribution between ICU and non-ICU patients.

**Fig 2:**
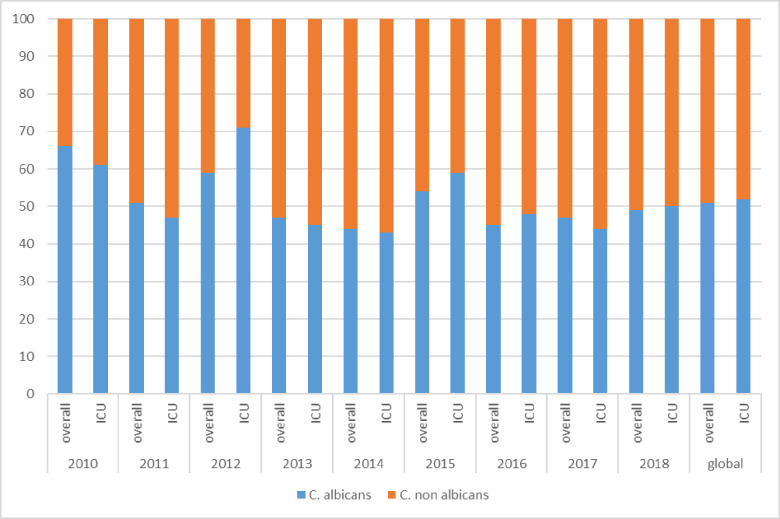
Ratio variation of *Candida albicans*/other *Candida* species isolation over nine years.

Fig 2 depicts the proportion of *C*. *albicans /* other *Candida* spp. during the study period. In the years 2011, 2013, 2014, 2016 and 2017 the isolates of *Candida* spp. other than *C*. *albicans* exceeded those of *C*. *albicans* in ICU-patients.

**Fig 3:**
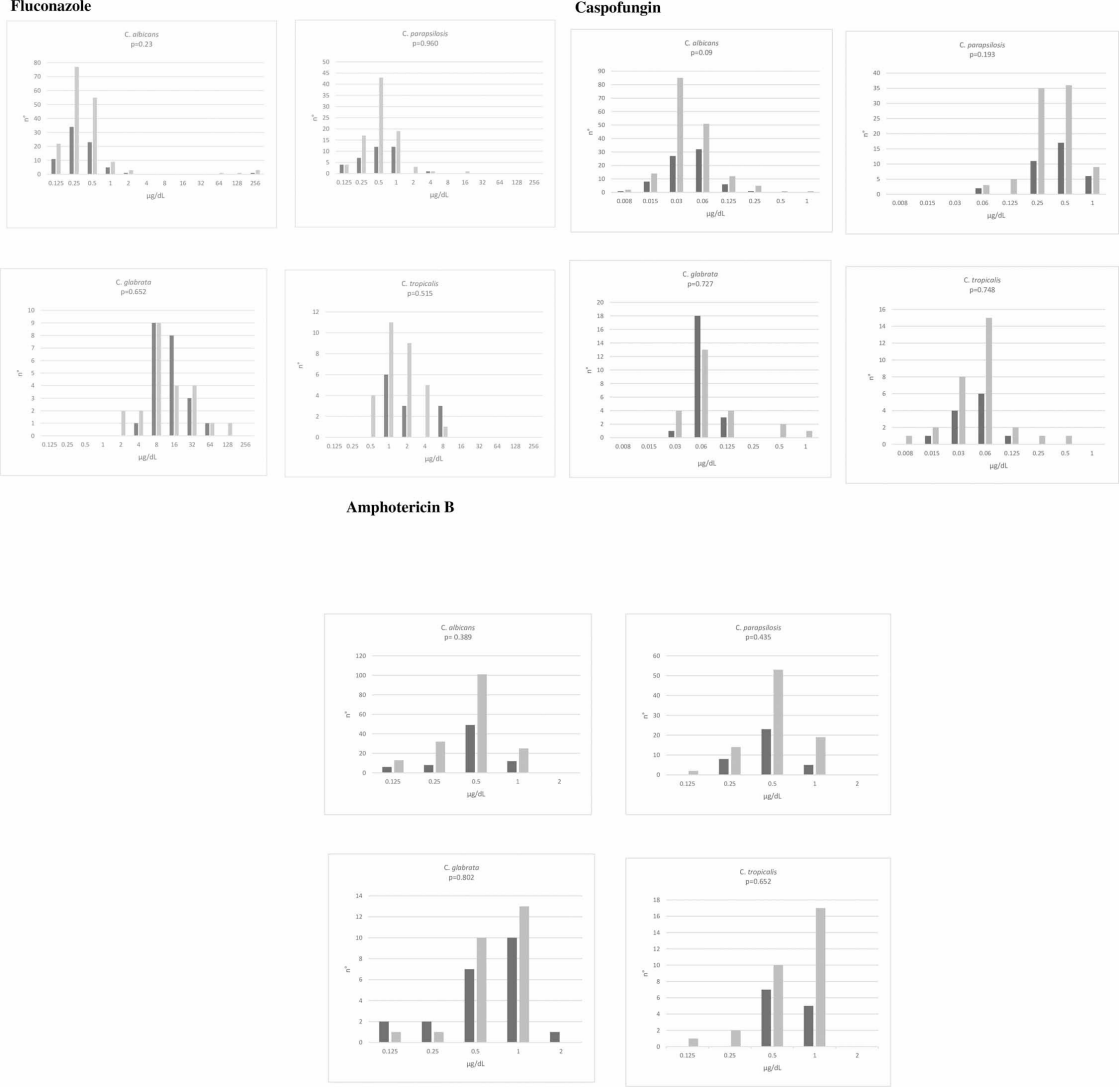
Fluconazole, caspofungin and amphotericin-B MIC distribution for strains of *Candida* spp. isolated from ICU (black bars) and non-ICU patients (grey bars).

Fig 3 shows antifungal susceptibility patterns of the isolates belonging to the four most common *Candida* species to fluconazole, caspofungin and amphotericin B. MICs results were available for 78% of the isolated yeasts from ICU patients.

Fluconazole resistance accounted for 1%, 0%, 5% and 25% of isolates of *C*. *albicans* (1/75), *C*. *parapsilosis* (0/36), *C*. *glabrata* (1/22) and *C*. *tropicalis* (3/12), respectively. With the exception of one isolate of *C*. *glabrata* (5%) which showed an amphotericin B MIC of 2.0 μg/ml, all isolates were susceptible to both caspofungin and amphotericin B. MIC distributions between strains isolated from ICU- and non- ICU patients were similar for all antifungal agents/*Candida* species (Fig 3).

### Antifungal therapy

Antifungal therapy was considered to be appropriate in about half of the cases (49%), with azoles (mainly fluconazole) being the most commonly used drugs (42%), followed by echinocandins (25%) (Table 1). When comparing ICU *vs* non-ICU patients, the proportion of untreated patients of the former group was higher (32% *vs* 20%).

### Outcome

**Fig 4:**
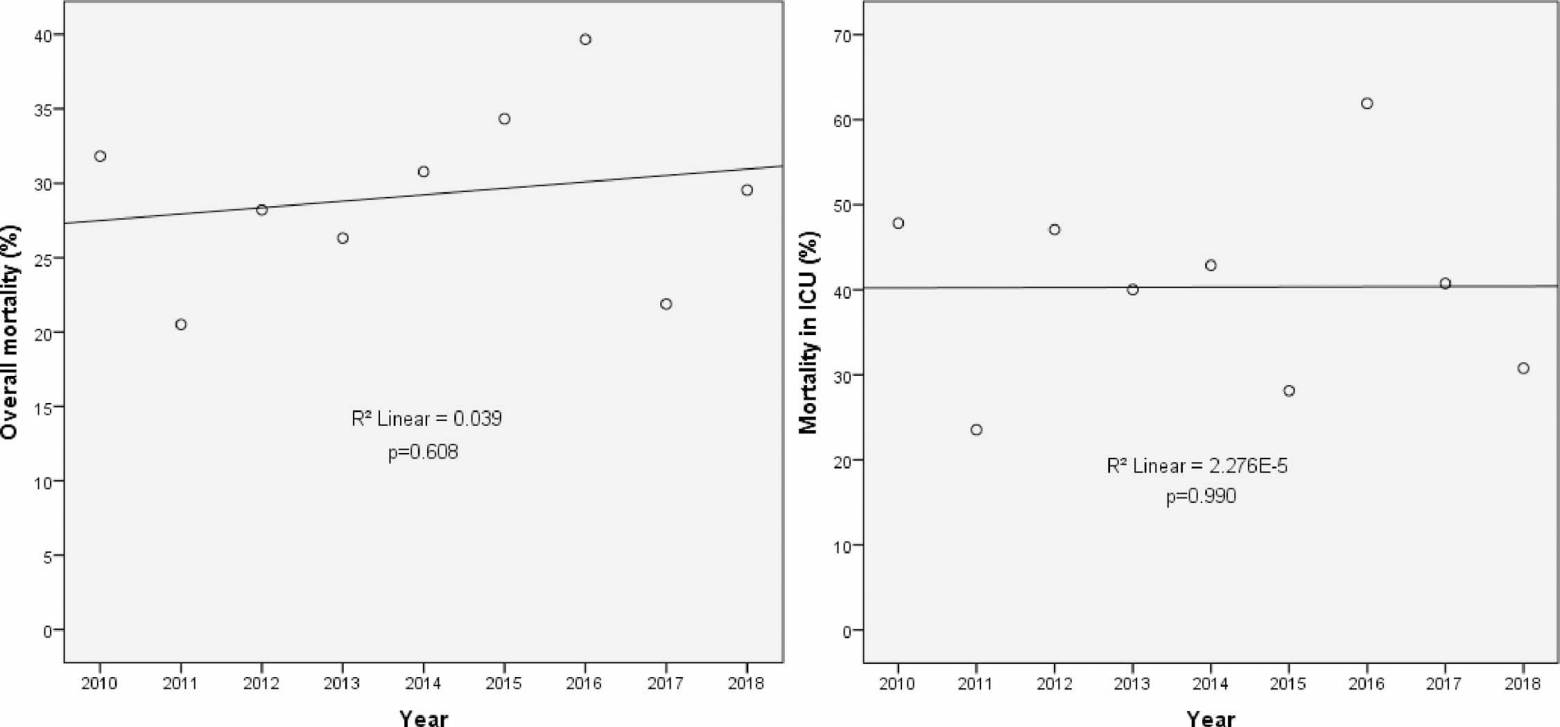
Mortality rate of candidemia over nine years in the overall population (A) and in ICU patients (B).

Both early or late mortality rates were higher in ICU- than in non-ICU patients and this difference reached a statistical significance on day 30 post-infection (41% *vs* 23%) (Table 1). Neither the overall mortality rate nor the mortality rate of ICU- patients with candidemia increased significantly over time (Fig 4).

The risk of death in ICU patients was then analyzed according to its timing (early [day 7] or late [day 30]) using logistic regression analysis. All variables with a p<0.05 at univariate analysis were introduced into the model. On day 7 post-infection, the following variables were significantly more common in patients with unfavorable outcome: septic shock, acute kidney failure, pulmonary embolism and lack of antifungal therapy while primary therapy with azoles was more common in surviving patients (Table 2).

**Table 2:** Characteristics of ICU patients with candidemia: 7-day mortality.

Characteristics	Survival	Death	*p* value ^a^
	(n = 153)	(n = 35)	
Male sex, *n (%)*	95 (62)	21 (60)	0.971
Age, median (IQR) ^b^	71 (62–77)	76 (67–80)	0.051
Chronic pulmonary diseases, *n (%)* ^c^	31 (20)	9 (26)	0.630
Haematological malignancy, *n (%)*	5 (3)	1 (3)	>0.999
Cardiovascular diseases, *n (%)* ^d^	111 (72)	23 (66)	0.549
Neurological diseases, *n (%)* ^e^	45 (29)	6 (17)	0.207
Gastrointestinal diseases, *n (%)* ^f^	30 (19)	9 (26)	0.567
Diabetes mellitus, *n (%)*	43 (28)	5 (14)	0.140
Chronic renal failure, n (%)	37 (24)	5 (14)	0.297
Solid tumors, n (%)	28 (18)	9 (26)	0.448
Charlon’s score, median (IQR)	5 (3–7)	6 (3–7)	0.385
Previous any surgery (<30 days), *n (%)*	78 (51)	18 (51)	>0.999
Gastrointestinal surgery, *n (%)*	14 (9)	5 (14)	0.358
Cardiovascular surgery, *n (%)*	53 (35)	8 (23)	0.253
Renal replacement therapy, *n (%)*	20 (13)	9 (26)	0.112
Central venous catheter, *n (%)*	144 (94)	33 (94)	>0.999
Central venous catheter-related BSIs, *n (%)* ^g^	94 (62)	25 (71)	0.385
Early central venous catheter removal, *n (%)*^h^	31 (20)	5 (14)	0.567
Other devices, *n (%)* ^i^	142 (93)	33 (94)	>0.999
Previous invasive procedures (<72 hours), *n (%)* ^j^	63 (41)	16 (46)	0.786
Parenteral nutrition, *n (%)*	94 (62)	20 (57)	0.748
Steroid therapy, n (%)	39 (26)	13 (37)	0.247
Immunosuppressive therapy, *n (%)* ^k^	3 (2)	1 (3)	0.567
Neutropenia, *n (%)*	2 (1.3)	0 (0)	>0.999
Acute kidney failure, *n (%)*	19 (12)	11 (31)	0.013
Septic shock, *n (%)*	29 (19)	16 (46)	0.002
Pneumonia, *n (%)*	55 (36)	17 (49)	0.244
Pulmonary embolism, *n* (%)	4 (3)	4 (11)	0.042
*Candida* species			0.501
*Candida albicans*, *n (%)*	80 (52)	18 (51)	
*Candida parapsilosis*, *n (%)*	38 (25)	7 (20)	
*Candida tropicalis*, *n (%)*	12 (8)	1 (3)	
*Candida glabrata*, *n (%)*	19 (12)	7 (20)	
Other *Candida* species, *n (%)* ^l^	4 (3)	2 (6)	
Appropriate antifungal therapy, *n (%)* ^m^	76 (50)	15 (43)	0.566
Primary antifungal therapy			
Azoles, *n (%)*	70 (46)	9 (26)	0.048
Echinocandins, *n (%)*	40 (26)	8 (23)	0.851
No treatment, *n (%)*	42 (27)	18 (51)	0.011

*a* Comparisons between groups were performed using Chi-Square test or Fisher Exact Test when expected frequencies were less than five

*b* IQR, Interquartile range

*c* Chronic pulmonary diseases include asthma, chronic bronchitis, emphysema and lung fibrosis

*d* Cardiovascular diseases include heart failure, ischemic heart disease, endocarditis and arrhythmia

*e* Neurological diseases include Parkinson’s disease, Alzheimer’s disease and paralysis

*f* Gastrointestinal diseases include Crohn’s disease, ulcerative colitis, chronic pancreatitis and gallbladder stones

*g* A catheter-related candidemia was defined according to the guidelines of the infectious diseases society of America (IDSA: Mermel et al. [16])

*h* Early central venous catheter removal was considered occurring within 48 h from blood cultures drawing

*i* Other devices include urinary catheter, surgical drainage, cutaneous gastrostomy and tracheostomy tube

*j* Previous invasive procedures include endoscopy and positioning of any device

*k* Immunosuppressive therapy include calcineurin inhibitors and monoclonal antibodies

*l* Other *Candida* species included *Candida guilliermondii* (n = 4) and one isolate each of *Candida krusei* and *Candida utilis*

*m* Appropriate antifungal therapy was considered when the appropriate drug with adequate dosage was started within 72 hours the first blood culture performed

On day 30 post-infection, septic shock and acute kidney failure were significantly more common in patients with unfavorable outcome while cardiovascular surgery was more common in surviving patients (Table 3).

**Table 3:** Characteristics of ICU patients with candidemia: 30-day mortality.

Characteristics	Survival	Death	*p* value ^a^
	(n = 111)	(n = 77)	
Male sex, *n (%)*	71 (64)	45 (58)	0.540
Age, median (IQR) ^b^	71 (61–78)	71 (60–77)	0.700
Chronic pulmonary diseases, *n (%)* ^c^	23 (21)	17 (22)	0.966
Haematological malignancy, *n (%)*	3 (3)	3 (4)	0.690
Cardiovascular diseases, *n (%)* ^d^	81(73)	53(69)	0.650
Neurological diseases, *n (%)* ^e^	29 (26)	22 (29)	0.838
Gastrointestinal diseases, *n (%)* ^f^	20 (18)	19(25)	0.355
Diabetes mellitus, *n (%)*	29(26)	19(25)	0.957
Chronic renal failure, n (%)	24 (22)	18 (23)	0.916
Solid tumors, n (%)	18 (16)	19 (25)	0.212
Charlon’s score, median (IQR) ^b^	5 (4–7)	5 (4–7)	0.101
Previous any surgery (<30 days), *n (%)*	61 (55)	35 (45)	0.257
Gastrointestinal surgery, *n (%)*	10 (9)	9 (12)	0.724
Cardiovascular surgery, *n (%)*	44 (39)	17 (22)	0.018
Renal replacement therapy, *n (%)*	13 (12)	16 (21)	0.144
Central venous catheter, *n (%)*	104 (94)	73 (95)	>0.999
Central venous catheter-related BSIs, *n (%)* ^g^	65 (59)	54 (70)	0.165
Early central venous catheter removal, *n (%)* ^h^	23 (21)	13 (17)	0.639
Other devices, *n (%)* ^i^	103 (93)	72 (93)	>0.999
Previous invasive procedures (<72 hours), *n (%)* ^j^	43 (39)	36 (47)	0.372
Parenteral nutrition, *n (%)*	65 (59)	49 (64)	0.635
Steroid therapy, n (%)	26 (24)	26 (34)	0.175
Immunosuppressive therapy, *n (%)* ^k^	3 (3)	1(1)	0.644
Neutropenia, *n (%)*	1 (1)	1 (1)	>0.999
Acute kidney failure, *n (%)*	12 (11)	18 (23)	0.037
Septic shock, *n (%)*	17 (15)	28 (36)	0.002
Pneumonia, *n (%)*	39 (35)	33 (43)	0.384
Pulmonary embolism, n (%)	3 (3)	5 (6)	0.277
*Candida* species			0.443
*Candida albicans*, *n (%)*	59 (53)	39 (51)	
*Candida parapsilosis*, *n (%)*	26 (23)	19 (25)	
*Candida tropicalis*, *n (%)*	10 (9)	3 (4)	
*Candida glabrata*, *n (%)*	12 (11)	14 (18)	
Other *Candida* species, *n (%)* ^l^	4 (4)	2 (3)	
Appropriate antifungal therapy, *n (%)* ^m^	53 (48)	38 (50)	0.993
Primary antifungal therapy			
Azoles, *n (%)*	53 (48)	26 (34)	0.078
Echinocandins, *n (%)*	25 (22)	23 (30)	0.334
No treatment, *n (%)*	32 (29)	28 (36)	0.352

*a* Comparisons between groups were performed using Chi-Square test or Fisher Exact Test when expected frequencies were less than five

*b* IQR, Interquartile range

*c*Chronic pulmonary diseases include asthma, chronic bronchitis, emphysema and lung fibrosis

*d* Cardiovascular diseases include heart failure, ischemic heart disease, endocarditis and arrhythmia

*e* Neurological diseases include Parkinson’s disease, Alzheimer’s disease and paralysis

*f* Gastrointestinal diseases include Crohn’s disease, ulcerative colitis, chronic pancreatitis and gallbladder stones

*g* A catheter-related candidemia was defined according to the guidelines of the infectious diseases society of America (IDSA: Mermel et al. [16])

*h* Early central venous catheter removal was considered occurring within 48 h from blood cultures drawing

*i* Other devices include urinary catheter, surgical drainage, cutaneous gastrostomy and tracheostomy tube

*j* Previous invasive procedures include endoscopy and positioning of any device

*k* Immunosuppressive therapy include calcineurin inhibitors and monoclonal antibodies

*l* Other *Candida* species included *Candida guilliermondii* (n = 4) and one isolate each of *Candida krusei* and *Candida utilis*

*m* Appropriate antifungal therapy was considered when the appropriate drug with adequate dosage was started within 72 hours the first blood culture performed

Table 4 shows independent factors for increased risk of death: septic shock, acute kidney failure, pulmonary embolism and lack of antifungal therapy (day 7) and septic shock (day 30).

**Table 4:** Risk factors associated with 7- and 30-day mortality in ICU patients with candidemia analyzed by logistic regression.

Factors		7-days mortality			30-days mortality			
	Hazard ratio	CI 95%		*p* value	Hazard ratio	CI 95%		*p* value
		Lower limit	Upper limit			Lower limit	Upper limit	
Cardiovascular surgery	-	-	-	**-**	1.985	0.996	3.958	0.051
Septic shock	3.583	1.510	8.501	0.004	2.465	1.195	5.083	0.015
Acute kidney failure	4.441	1.645	11.988	0.003	2.196	0.952	5.065	0.065
Polmunary embolism	6.492	1.226	34.365	0.028	-	-	-	**-**
Primary therapy with azoles	1.752	0.576	5.327	0.323	**-**	**-**	**-**	**-**
No antifungal therapy	4.072	1.704	9.732	0.002	-	-	-	-

## Discussion

In this study we analyzed the BSIs due to *Candida* spp. in ICU over nine years in a large italian university hospital. Although we observed a significant increase of the overall cumulative incidence of candidemia over time, the cumulative incidence in ICU was relatively stable. We registered a cumulative incidence of 9.9 cases per 1000 ICU admissions. This figure is somewhat similar to that reported in the literature. One multicenter study involving 23 european ICUs found a wide variation of cumulative incidence based on the type of ICU considered, with the lowest (1.7/1000) and the highest (19/1000) in surgical and medical ICUs, respectively [20]. When mixed ICUs (medical plus surgical) were considered, as in our study, a cumulative incidence of 8.4/1000 was found.

About half of our cases were caused by *Candida* species other than *C*. *albicans*, with *C*. *parapsilosis* being the most frequently isolated species. These data agree with those reported in the literature in the last years showing a general trend towards increasing isolation of non-*albicans* species which are generally less susceptible than *C*. *albicans* to common antifungal agents [6, 10, 11]. Antifungal resistance is a major threat for critically ill patients, especially in patients admitted in ICUs. Of note, the recent emergence of multidrug and pandrug resistant *C*. *auris* strains has raised concern among intensivists all around the world, and strict tracing of this infection is currently carried out by international organizations as CDC and ECDC [21, 22]. However, contrary to that observed by others [10, 14], resistance to antifungals was not of concern in our patients. Isolates of *C*. *tropicalis* represented an exception showing resistance to fluconazole in 25% of the cases. Overall, these data would indicate that, with the exception of the latter species in which an antifungal susceptibility result might be of some help to guide targeted therapy, early prescription of antifungals based on the most likely species and the known susceptibility profile of its wild-type isolates are recommended in our patients.

It is interesting to note that fluconazole represented the most frequent drug utilized in our patients as primary therapy. Among ICU patients, 42% were treated with fluconazole and 25% with an echinocandin. Although some studies found that receiving an echinocandin as first-line therapy reduced the death rate [23, 24], other reports revealed that the type of primary antifungal did not influence the outcome in these patients [25–27]. In the present study, outcome did not change according to initial antifungal therapy. Rather, there was a trend to better outcome in patients treated with fluconazole although the significance was lost after regression analysis. International guidelines consider echinocandins to be the first choice drugs in invasive candidiasis of critical and unstable patients due to several advantages over fluconazole in terms of spectrum of activity, pharmacodynamic properties, drug-drug interactions and toxicities [18, 28, 29]. However, fluconazole as first-line therapy represented a reasonable alternative to an echinocandin for ICU patients in our institution.

Surprisingly, we registered a high percentage of ICU patients (32%) who did not receive any antifungal treatment. This phenomenon, which has been already described in other series although in lower percentages [27, 30], is difficult to explain. One can speculate that the rapid clinical evolution of some patients along with a diagnostic delay might play a role in the lack of antifungal intervention. The lack of antifungal therapy in our patients impacted early deaths representing an independent risk factor for early poor prognosis.

Candidemia represents an infection associated with high morbidity and mortality. This is particularly evident in ICU patients that are often critical, unstable and with serious acute complications. Early and late mortality rates in our ICU patients were higher than those observed in non-ICU patients. This difference reached statistical significance on day 30 post-infection: 41% *vs* 23%. Overall mortality after candidemia has been reported to be up to 40% in the general population, rising to 50% in critically ill patients and 70% in patients with septic shock [14, 31–34].

Accordingly, we found septic shock to independently associated with increased risk for death. Our long-time period of observation allowed us to focus on trends in mortality. Contrary to what has been reported by others [27], we did not observe an increase in mortality over time in ICU patients. It is noteworthy how the type of management did not influence the infection outcome in our series. In particular, neither appropriate antifungal therapy nor timely catheter withdrawal were associated with better outcome, thereby suggesting that host-related factors might have more impact on mortality in ICU patients with candidemia rather than any early intervention.

The present study has some limitations. First, it is a retrospective observational study and the lack of a control group preclude any causality inference in this setting. This is particularly true for interventional variables, like the impact of therapies the results of which has to be interpreted cautionally. Second, since our data come from a single- center experience, our findings may not be relevant to other patient populations. It must be noted, however, that several ICUs differing target populations were involved in this study thus increasing the heterogeneity in terms of patient care. Third, although we have made every attempt to collect and analyze as much clinical data as possible to reveal useful information for the patients management, some biochemical and/or clinical data (i.e.: data for calculating SOFA and Apache scores in all patients) could not be explored because of missing data (especially in older cases).

In conclusion, neither cumulative incidence nor mortality rate of candidemia in ICU patients increased over time at our institution. However, mortality rate remained high and it was significantly associated with specific host-related factors in the majority of cases.

## Supporting information

S1 File (PDF)

S2 File (PDF)

S3 File (XLSX)
