# Supplementary material for: Candidemia in intensive care units over nine years at a large Italian university hospital: Comparison with other wards
Source: PLoS One. 2021 May 26;16(5):e0252165. doi: 10.1371/journal.pone.0252165 (PMC8153423; doi:10.1371/journal.pone.0252165)
Supplement: S1 File — (PDF) [file pone.0252165.s001.pdf]

n.	Age	Gender	Ward at the time of candidemi	Other chronic comorbidity	COPD	Diabetes	Haematological malignancies	Solid Tumors
1	48	1	2	2	2	2	2	1
2	23	2	2	2	2	2	2	2
3	30	1	2	2	2	2	2	2
4	35	2	2	2	2	2	2	2
5	38	1	2	1	2	2	2	2
6	33	2	2	2	2	2	2	2
7	43	1	3	2	2	2	2	1
8	32	2	2	2	2	2	2	2
9	46	2	1	2	2	2	2	2
10	46	2	1	2	2	2	2	2
11	44	1	1	1	2	2	2	2
12	40	1	1	2	2	2	2	2
13	49	1	2	1	2	2	2	2
14	45	2	1	2	2	2	2	2
15	49	1	3	2	2	2	2	2
16	39	1	2	1	2	2	2	2
17	25	1	3	2	2	2	2	2
18	35	1	2	1	2	2	2	2
19	31	2	1	1	2	2	2	2
20	48	2	2	2	2	2	1	2
21	53	1	1	2	2	2	2	2
22	41	1	1	2	2	2	2	2
23	23	2	2	1	2	2	2	2
24	46	2	2	1	2	2	2	2
25	46	2	2	1	2	2	2	2
26	57	1	2	1	2	1	2	2
27	54	2	1	2	2	1	2	2
28	54	2	2	2	2	2	2	2
29	54	2	1	2	2	2	2	2
30	57	2	1	2	2	2	2	2
31	78	1	1	2	2	2	2	2
32	30	2	3	1	2	2	2	2
33	23	2	2	1	2	2	2	2
34	76	2	1	2	2	2	2	2
35	46	2	1	2	2	2	2	2
36	51	2	1	1	2	2	2	1
37	29	2	1	1	2	2	2	2
38	52	1	1	1	2	2	2	2
39	57	1	1	2	2	2	2	2
40	62	2	1	2	1	2	2	2
41	69	1	1	2	2	2	2	2
42	57	1	2	2	2	2	2	1
43	52	1	1	2	2	2	2	2

44	68	2	2	1	2	2	2	2
45	54	1	1	2	2	2	2	2
46	62	1	2	2	2	2	2	2
47	59	1	1	2	2	2	2	2
48	61	2	2	2	2	2	2	2
49	65	2	1	2	2	2	2	2
50	46	2	1	2	2	2	2	1
51	67	2	3	2	2	2	2	2
52	51	2	3	2	2	1	2	2
53	58	2	3	2	2	1	2	2
54	65	2	1	1	2	2	2	2
55	65	2	1	2	2	2	2	2
56	60	2	2	2	2	2	2	2
57	60	2	2	2	2	2	2	2
58	63	1	2	2	2	2	2	2
59	61	1	2	2	2	2	2	2
60	18	1	2	2	2	2	2	2
61	68	2	1	2	1	2	2	2
62	69	2	1	2	1	2	2	2
63	65	2	1	2	2	2	2	2
64	64	1	1	2	2	2	2	2
65	65	1	1	2	2	2	2	2
66	69	2	1	2	1	2	2	2
67	69	2	3	2	1	2	2	2
68	18	1	2	2	2	2	2	2
69	71	2	1	2	2	2	2	2
70	51	2	1	2	2	2	2	2
71	38	1	1	2	2	2	2	2
72	50	1	2	1	2	2	1	2
73	32	2	3	2	2	2	2	1
74	67	2	3	2	2	2	2	1
75	64	1	1	2	2	2	2	2
76	58	2	2	1	2	2	2	1
77	61	2	2	2	2	2	2	1
78	68	2	1	2	2	2	2	2
79	72	1	1	2	1	2	2	2
80	65	2	1	1	2	2	2	2
81	68	1	2	2	2	2	2	2
82	62	2	1	2	2	2	2	2
83	76	2	1	2	1	2	2	2
84	61	2	3	2	2	2	2	2
85	65	2	2	2	2	1	2	2
86	69	1	1	2	1	1	2	2
87	62	1	2	1	2	2	2	1
88	61	2	2	2	2	2	2	1
89	62	2	2	2	2	2	2	2
90	62	2	3	2	2	2	2	1

91	52	2	3	1	2	2	2	2
92	75	2	1	2	2	2	2	2
93	48	2	2	2	2	2	2	2
94	64	1	1	2	2	2	2	2
95	76	2	1	2	2	2	2	2
96	66	2	1	2	2	1	2	2
97	72	2	1	2	2	2	2	2
98	61	2	3	2	2	2	2	2
99	61	2	1	2	2	1	2	2
100	52	2	2	1	2	2	2	2
101	78	1	1	2	2	2	2	2
102	78	1	2	2	2	2	2	2
103	63	2	3	2	2	2	2	1
104	56	2	3	2	2	2	2	2
105	55	2	3	1	2	2	2	1
106	77	1	1	2	2	2	2	2
107	55	2	3	1	2	2	2	1
108	62	2	2	2	2	2	1	2
109	66	1	2	2	2	2	2	1
110	75	2	2	2	2	2	2	2
111	72	2	1	2	2	2	2	2
112	69	2	3	2	2	2	2	1
113	62	2	1	2	2	1	2	2
114	93	1	3	2	2	2	2	2
115	64	1	2	2	2	2	2	1
116	63	1	2	2	2	2	2	1
117	25	2	2	1	2	2	2	2
118	68	2	2	2	2	1	2	2
119	60	1	1	2	2	2	2	1
120	60	2	3	2	2	2	2	1
121	79	1	2	2	2	2	2	2
122	34	2	1	2	2	2	2	2
123	34	2	1	2	2	2	2	2
124	75	1	1	2	2	2	2	2
125	62	2	1	1	2	2	2	2
126	60	2	2	2	2	1	2	2
127	57	2	1	1	2	2	2	2
128	64	2	3	2	2	2	2	2
129	66	1	2	2	2	2	2	1
130	76	2	1	2	2	2	2	2
131	77	2	2	2	2	2	2	2
132	58	2	1	2	1	2	2	1
133	76	2	1	2	2	2	2	2
134	76	2	1	2	2	2	2	2
135	78	1	1	2	2	2	2	2
136	40	2	2	2	2	2	2	2
137	66	1	1	2	2	1	2	2

138	72	2	3	2	2	2	2	2
139	68	1	2	2	2	2	1	2
140	74	1	3	2	2	2	2	2
141	71	2	1	2	2	2	2	2
142	66	1	1	2	2	1	2	2
143	57	2	2	1	2	2	2	2
144	76	1	1	2	1	2	2	2
145	50	2	2	2	2	2	2	1
146	62	1	3	1	2	2	2	2
147	62	1	2	1	2	2	2	2
148	60	2	1	2	2	2	2	2
149	63	1	2	2	2	2	2	2
150	74	1	1	2	1	2	2	2
151	71	2	1	2	2	2	2	2
152	53	2	2	2	2	2	2	1
153	78	2	3	2	2	2	2	2
154	69	2	1	2	1	2	2	2
155	69	2	1	2	1	2	2	2
156	78	2	3	2	2	2	2	2
157	76	1	3	2	2	2	2	2
158	78	2	3	2	2	2	2	2
159	75	2	2	2	2	2	2	2
160	79	2	3	2	2	2	2	2
161	66	1	1	2	2	1	2	2
162	50	1	2	1	2	2	2	2
163	79	1	2	2	2	2	2	2
164	60	1	1	1	2	2	2	2
165	60	1	1	1	2	2	2	2
166	71	2	1	2	2	1	2	2
167	75	1	3	2	2	2	2	2
168	59	2	2	2	2	2	2	2
169	48	2	3	1	2	1	2	2
170	73	1	2	1	1	2	2	2
171	78	2	2	2	2	2	2	2
172	18	2	3	2	2	2	2	2
173	42	1	3	2	2	2	2	1
174	34	1	2	2	2	2	2	2
175	51	2	2	1	2	1	2	2
176	56	2	2	1	2	2	2	1
177	74	2	1	2	2	1	2	2
178	78	1	1	2	2	2	2	2
179	62	2	1	1	2	2	2	2
180	61	2	1	1	2	2	2	2
181	77	1	1	2	2	2	2	2
182	74	1	2	1	1	2	2	2
183	78	1	1	2	2	2	2	2
184	77	2	1	2	1	1	2	2

185	72	1	2	2	2	2	2	2
186	95	1	1	1	2	2	2	2
187	83	2	1	2	2	2	2	2
188	81	2	1	2	2	1	2	2
189	84	2	2	2	2	2	2	2
190	86	2	1	2	2	2	2	2
191	85	2	3	2	2	2	2	2
192	58	2	2	2	2	2	2	1
193	82	2	3	2	1	2	2	2
194	76	1	1	2	2	1	2	2
195	87	2	3	2	2	2	2	2
196	79	2	1	2	1	1	2	2
197	83	1	1	2	1	2	2	2
198	80	2	3	2	2	2	2	2
199	77	2	3	1	1	2	2	2
200	73	1	1	2	1	1	2	2
201	83	1	1	2	2	2	2	2
202	82	1	1	2	1	2	2	2
203	72	2	3	2	2	1	2	2
204	78	2	2	2	2	1	2	2
205	80	2	1	1	1	2	2	2
206	93	2	1	2	2	2	2	2
207	53	2	3	2	2	2	2	1
208	88	1	2	2	2	2	2	2
209	71	1	3	2	2	1	2	2
210	53	1	1	1	2	2	2	1
211	81	2	3	2	2	2	2	2
212	71	1	1	2	2	1	2	2
213	76	2	2	2	2	2	2	1
214	84	1	1	2	2	2	2	2
215	81	1	3	2	2	2	2	2
216	76	2	1	2	1	1	2	2
217	78	2	1	2	1	2	2	2
218	77	2	1	2	2	1	2	2
219	69	1	3	1	2	2	2	2
220	75	1	3	2	2	2	2	1
221	72	1	3	2	2	2	2	1
222	88	2	1	2	2	2	2	2
223	72	1	2	2	2	2	2	1
224	77	2	2	2	2	2	2	1
225	71	2	3	1	2	1	2	2
226	83	1	1	2	2	2	2	2
227	78	2	3	2	2	2	2	2
228	76	2	1	2	1	1	2	2
229	80	2	1	2	1	1	2	2
230	76	2	2	2	2	1	2	2
231	72	2	1	2	1	2	2	2

232	74	2	1	2	2	1	2	2
233	77	2	2	2	2	2	2	1
234	76	1	3	2	2	2	2	1
235	72	2	2	2	1	1	2	2
236	69	2	1	2	2	2	2	2
237	83	2	3	2	1	1	2	2
238	71	2	3	2	2	1	2	2
239	67	1	2	1	2	2	2	2
240	76	1	2	2	2	2	1	2
241	65	2	3	1	2	2	2	1
242	75	1	3	2	2	2	2	1
243	70	2	2	1	2	2	2	2
244	88	2	2	2	2	2	2	2
245	75	1	3	2	2	2	2	1
246	75	1	1	2	2	2	1	2
247	69	1	1	1	2	2	2	1
248	86	1	2	2	1	2	2	2
249	92	2	2	2	2	1	2	2
250	75	1	2	2	2	2	2	1
251	76	2	2	2	2	2	1	2
252	60	1	2	2	2	2	2	2
253	70	2	2	1	1	1	1	2
254	87	1	1	1	2	2	2	2
255	87	1	1	2	1	2	2	2
256	83	2	1	2	2	1	2	2
257	78	2	2	2	2	2	1	2
258	73	2	1	2	1	1	1	2
259	71	1	2	2	2	2	2	1
260	87	2	1	2	2	1	2	2
261	91	1	2	2	1	2	1	2
262	84	2	1	2	2	1	2	2
263	77	1	1	2	2	2	2	1
264	71	2	2	2	2	2	2	1
265	78	1	2	2	2	2	2	1
266	74	2	2	2	2	1	2	2
267	77	1	2	1	2	2	1	2
268	75	2	2	2	2	2	2	1
269	74	2	1	2	1	2	2	2
270	72	1	2	2	2	2	2	1
271	71	2	3	2	2	1	1	2
272	67	2	3	2	2	2	2	1
273	44	2	1	1	2	1	2	1
274	59	2	2	1	2	2	2	1
275	55	2	2	1	2	2	2	1
276	75	1	3	1	2	1	2	1
277	73	2	2	2	1	1	2	2
278	72	2	1	2	2	2	2	1

279	91	2	1	2	2	1	2	2
280	75	2	2	2	1	2	2	1
281	63	2	3	1	1	2	2	1
282	82	2	2	2	2	2	2	1
283	72	2	3	2	2	2	2	2
284	69	2	1	2	2	2	2	1
285	54	2	2	1	2	2	2	1
286	80	2	1	2	2	2	1	2
287	57	2	2	2	2	2	2	1
288	73	1	1	2	2	2	2	2
289	77	2	2	2	1	2	2	1
290	74	2	3	2	2	2	2	1
291	80	2	1	2	2	2	1	2
292	73	2	3	2	2	2	2	1
293	73	2	1	1	2	2	2	1
294	71	1	3	2	2	2	2	1
295	66	2	2	2	2	1	2	1
296	69	2	2	2	2	1	2	1
297	72	2	3	2	2	2	2	1
298	64	2	3	1	2	2	2	1
299	69	2	2	2	1	1	2	1
300	85	2	3	2	2	2	2	1
301	51	2	2	1	2	1	2	2
302	73	2	2	2	2	2	2	1
303	67	2	1	1	2	2	2	1
304	64	2	2	1	2	2	2	1
305	79	2	1	2	2	2	2	1
306	79	2	1	2	2	2	2	1
307	81	2	3	1	2	2	2	1
308	71	1	2	2	1	1	2	1
309	56	1	1	2	1	2	2	1
310	67	1	2	1	2	1	2	2
311	71	2	2	2	1	2	2	1
312	75	2	1	2	1	2	2	1
313	69	1	1	1	1	1	2	2
314	89	2	3	2	1	2	2	1
315	77	1	1	2	1	2	2	1
316	70	2	2	2	1	2	2	1
317	71	2	3	2	2	2	2	1
318	82	2	1	2	2	2	2	1
319	86	1	1	2	2	2	2	1
320	82	2	3	2	2	2	2	1
321	53	2	2	1	2	2	2	2
322	80	2	2	2	2	2	2	1
323	85	1	1	1	2	2	2	1
324	83	2	2	2	2	1	2	1
325	77	2	3	2	2	1	2	1

326	85	2	1	2	2	2	2	1
327	79	2	2	1	2	2	2	1
328	81	1	3	2	2	2	2	1
329	80	2	2	2	2	1	1	2
330	83	1	3	2	2	2	2	1
331	80	2	2	2	2	2	2	1
332	80	1	1	2	2	2	2	1
333	72	1	2	2	2	2	2	1
334	80	2	1	2	2	1	2	2
335	85	2	1	2	2	2	2	1
336	28	2	1	2	2	2	2	2
337	74	1	1	2	2	1	2	1
338	80	1	2	2	2	2	2	1
339	45	2	1	2	2	2	2	2
340	77	2	2	2	2	2	2	1
341	74	2	2	2	2	1	2	1
342	70	1	1	2	2	1	2	1
343	87	1	1	2	2	2	2	1
344	72	2	1	2	1	2	2	1
345	80	2	1	2	2	2	2	1
346	81	2	2	1	1	1	2	1
347	70	2	2	2	2	1	2	1
348	58	1	2	2	2	2	2	2
349	83	2	3	2	1	1	2	1
350	73	2	1	2	2	1	2	1
351	79	1	1	1	2	1	2	1
352	87	1	1	2	2	1	2	1
353	44	1	3	2	2	2	2	2
354	82	2	3	2	1	2	2	1
355	92	2	2	2	1	2	2	1
356	79	2	3	2	1	1	1	1
357	72	1	1	2	1	1	2	1
358	64	2	1	2	1	2	2	2
359	71	2	2	2	2	2	2	1
360	76	2	1	2	2	2	2	2
361	74	1	1	2	2	2	2	2
362	67	2	1	2	2	1	2	2
363	67	2	1	2	2	1	2	2
364	80	2	1	2	2	2	2	2
365	75	2	2	2	1	2	2	2
366	74	2	2	2	2	2	2	2
367	88	1	2	2	2	2	2	1
368	92	1	2	2	2	1	2	2
369	78	2	2	2	2	2	2	2
370	82	2	3	2	1	1	2	1
371	55	1	3	2	2	1	2	2
372	62	1	2	2	2	2	2	2

373	81	2	2	2	2	1	1	2
374	36	2	2	2	2	2	2	1
375	36	2	2	2	2	2	2	1
376	88	1	2	2	2	2	2	2
377	86	1	2	2	2	1	2	2
378	72	2	2	2	1	1	1	2
379	79	2	1	2	1	1	2	2
380	71	2	1	2	2	2	2	2
381	63	1	1	1	2	1	2	2
382	80	1	1	2	2	2	2	1
383	49	2	1	2	2	1	2	2
384	74	2	1	2	1	1	2	2
385	77	1	1	2	2	2	2	2
386	84	2	1	2	2	2	1	1
387	30	2	3	2	2	2	2	2
388	68	2	3	2	2	2	2	2
389	75	2	1	2	1	1	2	2
390	70	1	1	2	2	1	1	2
391	61	2	1	2	2	1	2	2
392	83	2	1	2	1	1	2	2
393	82	2	1	2	2	2	2	2
394	77	1	1	2	2	1	2	2
395	77	1	2	2	2	2	2	1
396	83	1	2	2	2	2	1	1
397	55	2	2	2	2	2	2	2
398	57	2	2	1	2	1	2	1
399	76	1	2	2	2	2	2	2
400	92	1	2	2	2	1	2	2
401	85	1	2	2	2	2	2	2
402	81	2	2	2	2	1	2	2
403	77	2	2	2	2	2	2	1
404	50	1	2	2	2	2	2	1
405	36	2	3	2	2	2	2	2
406	70	1	2	2	2	2	2	1
407	67	2	2	2	2	2	2	2
408	67	2	2	2	2	2	2	2
409	77	2	3	2	2	2	2	1
410	67	2	1	2	2	2	2	2
411	88	2	3	2	2	2	2	2
412	70	1	3	2	2	2	2	2
413	70	2	1	2	2	2	2	2
414	84	2	2	2	2	2	2	2
415	75	1	3	2	2	2	2	2
416	66	2	1	2	2	2	2	2
417	62	1	1	2	2	2	2	2
418	71	1	1	2	1	2	2	2
419	66	1	1	2	2	2	2	2

420	68	1	3	2	2	2	2	2
421	66	1	2	1	2	2	1	1
422	66	1	3	1	2	2	2	2
423	66	1	3	1	2	2	2	1
424	50	1	3	2	2	2	2	2
425	28	1	3	1	2	2	2	2
426	74	2	2	1	1	2	2	1
427	79	1	2	1	2	2	2	2
428	71	1	2	1	2	2	2	2
429	57	2	2	1	2	2	2	2
430	68	2	2	1	2	2	2	1
431	70	1	2	1	2	1	1	1
432	82	2	2	1	1	2	2	2
433	72	1	2	1	1	2	2	2
434	68	1	2	1	2	2	2	2
435	73	1	2	2	2	2	2	2
436	48	2	1	1	2	2	2	2
437	56	2	3	1	2	1	2	2
438	37	1	3	2	2	2	2	2
439	81	1	3	1	2	2	2	2
440	77	2	2	1	2	2	2	2
441	65	2	2	1	2	2	1	1
442	69	2	2	1	2	2	1	2
443	61	2	2	1	2	2	2	2
444	46	2	3	1	2	1	2	2
445	54	2	3	1	2	2	2	2
446	62	2	3	1	2	2	2	1
447	34	1	2	1	2	2	2	2
448	84	2	2	1	1	2	2	2
449	79	2	2	1	2	2	2	1
450	70	2	2	1	1	2	2	1
451	61	1	2	1	2	2	2	1
452	77	1	2	1	2	2	1	2
453	63	2	2	1	2	2	2	2
454	49	1	2	1	2	2	2	2
455	28	2	2	1	2	2	2	2
456	47	1	2	1	2	2	2	2
457	47	1	2	1	2	2	2	2
458	77	1	2	1	2	2	2	2
459	77	1	2	1	2	2	2	2
460	41	2	2	1	2	2	2	2
461	77	1	2	1	2	2	2	2
462	78	2	2	2	2	2	2	2
463	58	2	2	1	2	2	2	1
464	45	2	2	1	2	2	2	2
465	70	2	2	1	2	2	2	2
466	74	1	3	1	2	2	2	1

467	52	1	2	1	2	2	2	2
468	61	2	2	1	2	2	2	2
469	61	2	2	1	2	2	2	2
470	61	2	2	1	2	2	2	2
471	42	2	2	1	2	2	2	2
472	89	2	2	1	2	1	2	2
473	46	1	2	1	2	2	2	2
474	80	1	2	1	2	2	2	1
475	47	1	2	1	2	2	2	2
476	64	2	2	1	1	2	2	2
477	61	2	2	1	2	2	1	1
478	84	2	2	1	2	2	2	1
479	86	1	2	1	2	2	2	2
480	84	2	2	1	2	2	2	1
481	88	1	2	1	2	2	2	1
482	79	2	2	1	2	1	2	1
483	81	2	2	1	2	2	2	1
484	81	2	2	1	2	2	2	1
485	84	2	2	1	2	2	2	2
486	57	2	2	1	2	2	2	2
487	62	1	2	1	2	1	2	1
488	40	2	2	1	2	2	2	1
489	40	2	2	1	2	2	2	1
490	61	2	1	1	2	2	2	2
491	75	1	1	1	1	1	2	2
492	72	2	1	1	2	2	2	1
493	88	2	1	1	2	2	2	2
494	75	2	1	1	2	2	2	2
495	60	2	1	1	2	2	2	2
496	69	2	1	1	2	2	2	1
497	77	2	1	1	2	1	2	1
498	58	1	2	1	2	2	2	1
499	72	2	2	1	2	2	2	1
500	62	2	2	1	2	2	2	1
501	46	2	2	1	2	2	2	1
502	72	1	2	1	2	2	2	1
503	79	1	3	1	2	1	2	1
504	79	1	2	1	1	1	2	2
505	81	1	1	1	1	2	2	2

Legend:	1 Famle	1 ICU		1 yes
	2 Male	2 internal medicine wa		2 no
		3 surgical ward		

Hepatic chronic diseases	Gastrointestinal diseases	Cardiovascular diseases	Neurological diseases	Chronic kidney failure	Solid organ transplantation	Surgery within 6 month before	Gastrointestinal surgery	Cardiovascular surgery
2	2	2	2	2	2	2	2	2
2	2	2	2	2	2	2	2	2
2	2	2	1	2	2	1	2	2
2	2	1	2	2	2	2	2	2
1	1	2	2	2	2	2	2	2
2	2	1	2	2	2	2	2	2
2	2	2	2	2	2	1	1	2
2	1	2	1	2	2	2	2	2
2	1	1	2	2	2	2	2	2
2	1	1	2	2	2	2	2	2
2	1	2	1	2	2	2	2	2
2	2	1	2	2	2	1	2	1
2	2	2	2	1	1	2	2	2
2	2	2	1	2	2	2	2	2
2	2	1	1	2	2	1	2	2
1	2	2	1	2	2	2	2	2
2	2	1	1	2	2	1	1	2
2	2	1	2	2	2	1	2	1
1	2	1	2	2	2	1	2	1
2	2	2	2	1	2	2	2	2
2	2	2	1	2	2	1	2	2
2	1	1	1	2	2	1	2	2
1	2	1	2	2	2	2	2	2
1	2	1	2	2	2	1	2	1
1	2	1	2	2	2	1	2	1
2	1	2	2	2	1	1	2	2
2	2	2	2	2	2	1	2	1
2	2	2	1	2	2	2	2	2
2	2	2	1	2	2	2	2	2
2	2	1	1	2	2	1	2	1
2	2	2	2	2	2	2	2	2
1	2	1	2	2	2	2	2	2
1	1	2	2	2	2	2	2	2
2	2	2	2	2	2	1	2	1
2	2	2	2	2	2	2	2	2
2	2	2	2	2	2	2	2	2
1	2	1	2	2	2	1	2	1
2	2	1	1	2	2	1	2	1
2	1	1	2	2	2	2	2	2
2	2	2	2	2	2	1	2	2
2	1	2	1	2	2	2	2	2
2	2	2	2	2	2	2	2	2
2	2	1	1	2	2	2	2	2

2	2	1	2	1	1	2	2	2
2	2	1	1	2	2	2	2	2
2	1	2	1	2	2	2	2	2
2	2	1	2	2	2	1	2	1
2	2	2	1	2	2	2	2	2
2	2	2	1	2	2	2	2	2
2	2	1	2	2	2	1	1	2
2	2	1	2	2	2	1	2	2
2	2	1	2	2	2	1	1	1
2	2	1	2	2	2	1	2	1
2	2	2	1	2	2	2	2	2
2	1	2	1	2	2	1	1	2
2	1	1	2	2	2	2	2	2
2	1	1	2	2	2	2	2	2
2	1	2	2	2	2	1	1	2
2	1	2	2	2	2	2	2	2
2	1	1	1	1	2	2	2	2
2	2	1	2	2	2	1	2	2
2	1	1	2	2	2	1	2	1
2	2	1	2	2	2	1	2	1
2	2	1	2	2	2	1	2	1
2	2	1	2	2	2	2	2	2
2	1	1	2	2	2	1	2	1
2	1	1	2	2	2	1	1	2
2	1	1	1	1	2	2	2	2
2	2	2	1	2	2	2	2	2
2	2	1	1	2	2	1	2	1
2	2	2	2	2	2	1	2	2
1	2	2	2	2	2	2	2	2
2	2	2	2	2	2	1	1	2
2	1	2	2	2	2	1	1	2
2	2	1	2	2	2	1	2	1
1	1	2	2	2	2	2	2	2
2	2	2	2	2	2	2	2	2
2	2	1	2	2	2	1	2	1
2	1	2	2	2	2	2	2	2
2	2	1	2	2	1	1	2	1
2	1	1	2	2	2	2	2	2
2	2	1	2	2	2	1	2	1
2	1	1	2	2	2	1	1	1
2	2	2	2	2	2	1	2	2
2	2	2	2	2	2	2	2	2
2	2	1	2	2	2	1	2	1
2	1	2	2	2	2	2	2	2
2	2	2	2	2	2	2	2	2
2	1	1	1	2	2	2	2	2
2	2	2	2	2	2	2	2	2

1	2	2	2	2	2	1	2	2
2	2	1	2	2	2	2	2	2
2	1	2	2	2	2	1	2	2
2	2	1	1	2	2	1	2	1
2	2	1	2	1	2	1	1	1
2	2	1	2	1	2	1	2	1
2	2	1	2	2	2	1	1	2
2	2	2	2	2	2	1	2	2
2	1	2	2	2	2	1	1	2
1	2	2	2	2	2	2	2	2
2	2	1	2	1	2	1	2	1
2	2	1	2	2	2	2	2	2
2	2	2	2	2	2	1	2	2
2	2	2	2	2	2	1	2	2
1	2	2	2	2	1	1	1	2
2	2	1	2	2	2	1	2	1
1	2	2	2	2	1	1	1	2
2	2	2	2	2	2	2	2	2
2	2	2	2	2	2	2	2	2
2	2	1	2	2	2	1	1	2
2	1	2	1	2	2	2	2	2
2	1	2	2	2	2	1	1	2
2	1	1	2	1	2	2	2	2
2	2	2	2	2	2	1	2	2
2	2	2	2	2	2	1	2	2
2	2	2	2	2	2	2	2	2
2	1	2	2	2	2	1	1	2
2	2	1	2	2	2	2	2	2
2	1	2	2	2	2	2	2	2
2	1	2	2	2	2	1	1	2
2	2	2	1	2	2	2	2	2
2	1	2	2	2	2	2	2	2
2	1	2	2	2	2	2	2	2
2	2	2	1	2	2	2	2	2
2	2	1	2	1	2	1	2	1
2	2	1	2	2	2	1	2	1
2	2	1	1	1	2	2	2	2
2	2	2	2	2	2	2	2	2
2	2	2	2	2	2	2	2	2
2	2	1	2	2	2	1	2	1
2	2	1	2	2	2	2	2	2
2	1	2	2	2	2	1	1	2
2	2	1	2	2	2	1	2	1
2	2	1	2	2	2	1	2	1
2	2	1	2	2	2	1	2	1
2	1	2	2	2	2	1	2	2
2	2	1	2	1	2	1	2	2

2	2	1	2	2	2	2	2	2
2	2	2	2	2	2	2	2	2
2	2	1	2	2	2	1	2	2
2	2	1	2	2	2	1	2	1
2	2	1	2	1	2	1	2	2
2	2	1	1	1	2	2	2	2
2	2	2	1	2	2	2	2	2
2	2	2	1	2	2	1	2	2
2	1	2	2	2	2	1	1	2
2	1	2	2	2	2	2	2	2
2	2	2	2	2	2	1	2	2
2	2	2	2	2	2	1	2	2
2	2	1	2	2	2	1	2	1
2	2	2	2	2	2	2	2	2
2	1	2	1	2	2	2	2	2
2	2	1	1	2	2	1	2	2
2	2	1	2	1	2	2	2	2
2	1	1	2	1	2	2	2	2
2	2	1	1	2	2	1	2	2
2	2	1	2	2	2	1	2	2
2	2	1	1	2	2	1	2	2
2	2	1	1	2	2	2	2	2
2	2	2	2	2	2	2	2	2
2	2	1	2	1	2	1	2	2
1	1	2	2	2	2	2	2	2
2	2	1	1	2	2	2	2	2
2	2	2	2	2	2	2	2	2
2	2	2	2	2	2	2	2	2
2	2	2	1	2	2	2	2	2
2	2	2	2	2	2	2	2	2
2	2	2	2	2	2	2	2	2
1	2	1	1	1	2	2	2	2
2	2	1	1	2	2	2	2	2
2	2	1	2	2	2	1	2	1
2	2	2	2	2	2	1	2	1
2	2	2	2	1	2	1	2	2
2	1	2	2	2	2	1	1	2
1	1	2	2	2	2	1	1	2
2	2	2	2	1	1	1	2	1
2	2	2	2	1	2	1	2	1
2	2	1	1	2	2	2	2	2
1	2	1	2	2	2	2	2	2
1	1	1	2	2	2	1	2	1
2	2	1	2	2	2	1	2	1
2	2	1	1	2	2	2	2	2
2	2	1	2	2	2	1	1	2
2	2	1	2	2	2	2	2	2

2	2	1	2	2	2	1	2	1
2	2	1	1	2	2	2	2	2
2	1	1	2	1	2	2	2	2
2	2	1	2	2	2	2	2	2
2	2	1	2	2	2	2	2	2
2	2	1	2	2	2	2	1	1
2	2	1	2	2	2	1	2	1
2	2	1	2	2	2	1	2	2
2	2	1	2	2	2	1	2	1
2	2	1	2	2	2	2	2	2
2	1	1	2	1	2	1	2	1
2	2	1	2	2	2	1	2	1
2	2	1	2	2	2	1	2	1
2	2	1	2	2	2	2	2	2
1	2	1	2	2	2	1	2	2
2	2	1	2	2	2	2	2	2
2	2	1	2	2	2	2	2	2
2	2	1	2	2	2	2	2	2
2	2	1	2	2	2	1	2	2
2	2	1	1	2	2	2	2	2
2	2	1	2	2	2	1	2	1
2	1	1	2	2	2	2	2	2
2	1	1	2	2	2	1	1	2
2	2	1	1	2	2	2	2	2
2	2	1	2	2	2	1	2	2
2	2	1	2	2	2	2	2	2
2	1	1	1	2	2	2	2	2
2	2	1	2	2	2	1	2	1
2	1	2	2	2	2	1	1	2
2	2	1	1	2	2	2	2	2
2	2	1	2	2	2	1	2	1
2	1	2	2	1	2	1	2	1
2	2	1	2	1	2	1	2	2
2	2	1	2	2	2	1	2	1
1	2	2	2	2	2	1	2	2
2	1	2	2	2	2	1	1	2
2	2	2	2	2	2	1	2	2
2	2	1	2	1	2	1	2	2
2	2	2	2	2	2	1	2	2
2	1	2	2	2	2	2	2	2
2	2	1	2	2	2	1	2	1
2	1	1	2	2	2	1	2	1
2	1	1	1	1	2	1	2	1
2	2	2	2	1	2	2	2	2
2	1	1	2	2	2	1	1	2
2	1	1	2	2	2	2	2	2
2	2	1	1	2	2	1	1	2

2	1	2	2	2	2	2	2	2
2	1	2	2	2	2	2	2	2
2	2	2	2	2	2	1	2	2
2	1	1	1	2	2	2	2	2
2	2	2	2	2	2	1	2	1
2	2	1	2	2	2	1	2	1
2	1	2	2	2	2	1	2	2
1	1	2	2	2	2	2	2	2
2	2	1	1	2	2	2	2	2
1	2	2	2	2	1	1	1	2
2	1	2	2	2	2	1	1	2
1	2	1	2	2	1	2	2	2
2	1	2	2	2	2	2	2	2
2	1	2	2	2	2	1	1	2
2	2	1	1	2	2	2	2	2
1	2	2	2	2	2	2	2	2
2	2	1	2	1	2	1	2	2
2	2	2	2	1	2	2	2	2
2	1	2	2	2	2	2	2	2
2	2	1	2	2	2	2	2	2
2	1	2	2	2	2	1	2	2
1	2	2	2	2	2	1	2	2
2	2	1	1	1	2	2	2	2
2	2	1	2	1	2	1	2	1
2	2	1	2	2	2	2	2	2
2	2	2	2	2	2	2	2	2
2	2	1	2	2	2	2	2	2
2	1	2	2	2	2	2	2	2
2	2	1	2	1	2	2	2	2
2	2	1	2	1	2	1	2	2
2	2	1	2	1	2	1	2	1
2	1	2	2	2	2	1	1	2
2	2	2	2	2	2	2	2	2
2	2	2	2	2	2	2	2	2
2	2	2	2	2	2	1	2	2
1	1	1	2	2	2	2	2	2
2	2	2	2	2	2	2	2	2
2	1	1	1	1	2	2	2	2
2	1	2	2	2	2	2	2	2
2	1	2	2	1	2	1	1	2
2	2	2	1	2	2	1	1	2
1	2	1	2	2	2	1	2	1
1	2	1	2	2	2	1	2	1
1	1	1	2	2	2	2	2	2
2	1	2	2	2	2	1	1	2
2	1	1	1	1	2	2	2	2
2	2	2	2	2	2	2	2	2

2	1	1	1	1	2	2	2	2
2	2	2	2	2	2	1	1	2
1	1	2	2	2	2	2	2	2
2	1	2	2	2	2	2	2	2
2	1	2	2	2	2	2	2	2
2	2	1	2	2	2	1	1	2
1	2	2	2	1	2	2	2	2
2	2	1	1	1	2	2	2	2
2	2	1	2	1	2	2	2	2
2	2	2	2	2	2	1	1	2
2	2	2	2	2	2	2	2	2
2	2	1	2	2	2	1	2	2
2	2	1	1	1	2	2	2	2
2	2	1	2	2	2	1	2	2
1	1	2	2	2	2	1	1	2
2	1	1	2	2	2	1	2	2
2	2	1	2	2	2	2	2	2
2	1	1	2	2	2	2	2	2
2	2	2	1	2	2	1	1	2
1	1	1	2	2	2	1	1	2
2	2	1	2	2	2	2	2	2
2	2	2	2	2	2	1	1	2
1	2	2	1	2	2	2	2	2
2	2	1	2	2	2	2	2	2
1	1	1	2	2	2	2	2	2
1	2	1	2	2	2	1	2	2
2	2	1	2	2	2	1	2	1
2	2	1	2	2	2	1	2	1
2	1	2	2	2	2	1	2	2
2	2	2	2	2	2	2	2	2
2	2	1	2	1	2	1	2	1
2	1	1	1	2	2	2	2	2
2	2	1	2	2	2	1	2	2
2	2	1	2	2	2	1	2	1
2	2	1	2	1	2	2	2	2
2	2	2	2	2	2	2	2	2
2	2	1	2	2	2	1	2	2
2	1	1	2	2	2	2	2	2
2	1	1	1	2	2	1	1	2
2	2	1	2	2	2	1	2	1
2	1	2	1	2	2	1	1	2
2	2	1	2	2	2	1	2	2
2	2	2	2	2	1	1	2	2
2	2	1	2	2	2	2	2	2
2	2	1	2	2	2	2	2	2
2	2	2	2	2	2	2	2	2
2	2	1	2	2	2	1	2	2

2	2	1	2	2	2	2	2	2
1	2	1	2	2	2	2	2	2
2	1	1	2	2	2	1	1	2
2	1	2	2	2	2	2	2	2
2	2	1	2	2	2	1	1	2
2	1	2	2	1	2	2	2	2
2	2	1	1	2	2	2	2	2
2	2	1	2	1	2	1	1	2
2	2	2	2	2	2	1	1	2
2	1	1	1	2	2	1	2	2
2	1	2	2	2	2	2	2	2
2	1	1	1	2	2	2	2	2
2	1	1	2	2	2	2	2	2
2	2	2	2	2	2	1	2	2
2	2	1	2	1	2	1	2	2
2	2	1	1	2	2	2	2	2
2	2	1	1	2	2	1	1	2
2	2	1	1	2	2	2	2	2
2	1	1	2	1	2	1	2	1
2	2	1	2	1	2	1	2	2
2	1	1	2	2	2	2	2	2
2	2	1	2	1	2	1	1	2
2	1	2	2	2	2	2	2	2
2	2	1	2	2	2	1	2	2
2	2	2	1	1	2	1	2	1
1	2	1	1	2	2	2	2	2
2	2	1	1	2	2	2	2	2
2	2	2	2	2	2	1	2	2
2	2	1	2	1	2	1	1	2
2	2	1	2	1	2	2	2	2
2	2	1	2	2	2	1	2	1
2	2	1	1	1	2	2	2	2
2	2	1	2	2	2	1	2	1
2	2	2	2	2	2	2	2	2
2	2	1	2	2	2	1	2	1
2	2	2	1	2	2	1	2	1
2	2	1	2	2	2	1	2	2
2	2	1	2	2	2	1	2	2
2	1	1	2	2	2	1	2	1
2	1	1	1	2	2	2	2	2
2	1	1	1	1	2	2	2	2
2	2	1	2	2	2	2	2	2
2	2	2	1	1	2	2	2	2
2	2	1	1	1	2	2	2	2
2	2	1	2	2	2	1	2	2
2	1	1	2	2	2	1	2	2
2	1	2	1	2	2	2	2	2

2	2	2	2	2	2	2	2	2
2	2	2	2	2	2	2	2	2
2	2	2	2	2	2	2	2	2
2	2	2	1	2	2	2	2	2
2	2	1	1	1	2	2	2	2
2	2	1	2	2	2	2	2	2
2	2	2	1	2	2	2	2	2
2	2	2	2	1	2	2	2	2
1	2	1	2	2	2	1	2	2
2	2	2	2	2	2	2	2	2
2	2	2	2	2	2	2	2	2
2	2	1	1	1	2	1	2	2
2	2	2	2	2	2	2	2	2
2	2	1	2	1	2	2	2	2
2	2	2	2	2	2	1	2	2
2	2	2	2	2	2	1	2	2
2	2	1	1	1	2	2	2	2
2	2	1	2	2	2	2	2	2
2	2	1	1	2	2	2	2	2
2	2	1	1	1	2	2	2	2
2	2	1	2	2	2	1	2	1
2	2	1	2	2	2	2	2	2
2	2	2	2	2	2	2	2	2
2	2	2	2	2	2	2	2	2
2	2	1	1	2	2	2	2	2
1	2	2	2	2	2	2	2	2
2	2	2	1	2	2	2	2	2
2	2	2	1	2	2	2	2	2
2	2	1	2	2	2	2	2	2
2	2	2	1	2	2	2	2	2
2	2	1	2	2	2	2	2	2
2	2	1	1	2	2	2	2	2
2	2	2	2	2	2	2	2	2
2	2	1	2	2	2	1	2	2
2	2	2	1	2	2	2	2	2
2	2	2	1	2	2	2	2	2
2	2	2	2	2	2	1	2	2
2	2	1	2	2	2	1	2	2
2	2	1	2	2	2	1	2	2
2	2	1	1	2	2	1	2	2
2	2	1	2	1	2	2	2	2
2	2	1	1	2	2	2	2	2
2	2	1	2	1	2	1	2	1
2	2	1	2	1	2	1	2	1
2	2	1	2	2	2	2	2	2
2	2	1	2	1	2	1	2	1
2	2	1	2	1	2	2	2	2

2	2	1	2	2	2	1	2	1
2	2	2	2	2	2	2	2	2
1	1	1	2	1	1	1	1	2
2	2	1	2	2	2	1	1	2
2	2	2	2	2	2	1	2	2
2	2	1	2	1	2	1	2	1
2	1	1	2	2	2	2	2	2
2	2	1	1	2	2	1	2	1
2	2	1	2	2	2	2	2	2
2	2	2	1	2	2	2	2	2
2	2	1	2	2	2	2	2	2
2	1	1	1	2	2	2	2	2
2	2	2	2	2	2	1	1	1
2	2	1	2	2	2	1	2	1
2	2	1	2	2	2	1	2	1
2	2	2	2	2	2	1	1	2
1	2	2	1	2	2	2	2	2
2	2	2	2	1	2	2	2	2
2	2	2	2	2	2	1	2	2
2	1	1	2	1	2	1	1	2
2	2	1	2	2	2	2	2	2
2	2	2	2	2	2	2	2	2
2	2	2	2	2	2	2	2	2
1	1	2	2	2	1	2	2	2
2	2	1	2	2	2	1	2	2
2	2	1	2	2	2	1	2	2
2	2	2	2	2	2	1	2	2
2	2	1	2	2	2	2	2	2
2	2	1	1	1	2	2	2	2
1	2	1	2	2	2	1	2	1
1	1	1	1	1	2	2	2	2
2	1	2	2	2	2	2	2	2
2	1	1	2	2	2	2	2	2
2	1	1	2	2	2	2	2	2
2	2	2	2	2	2	2	2	2
2	2	2	1	2	2	2	2	2
2	1	2	1	2	2	2	2	2
2	1	2	1	2	2	2	2	2
2	1	1	2	2	2	1	2	2
2	1	1	2	2	2	1	2	2
1	2	1	2	2	2	2	2	2
2	2	1	1	1	2	2	2	2
2	2	2	2	2	2	2	2	2
2	2	2	1	2	2	1	2	2
2	2	2	1	2	2	2	2	2
2	2	1	2	2	2	2	2	2
2	2	1	1	2	2	2	2	2

2	1	2	2	2	2	2	2	2
1	1	2	2	2	1	2	2	2
1	1	2	2	2	1	2	2	2
1	1	2	2	2	1	2	2	2
2	2	1	2	2	2	1	2	1
2	2	2	1	2	2	2	2	2
2	2	2	2	2	2	2	2	2
2	2	1	2	1	2	2	2	2
2	1	2	1	2	2	2	2	2
2	2	2	1	2	2	2	2	2
2	2	2	1	2	2	2	2	2
2	2	1	2	2	2	2	2	2
2	2	2	1	2	2	2	2	2
2	2	2	2	1	1	2	2	2
2	2	2	2	2	2	1	1	2
2	2	2	2	2	2	2	2	2
2	1	1	2	2	2	2	2	2
2	1	1	2	2	2	2	2	2
2	1	1	2	2	2	2	2	2
2	2	2	1	2	2	1	2	2
2	2	2	2	2	2	2	2	2
2	2	2	2	2	2	2	2	2
2	2	2	2	2	2	2	2	2
2	2	2	1	2	2	2	2	2
2	2	1	2	2	2	2	2	2
2	2	2	2	2	2	2	2	2
2	2	1	2	1	2	2	2	2
2	1	1	2	1	2	1	1	2
1	1	1	2	2	2	2	2	2
2	2	1	1	2	2	2	2	2
2	2	1	1	2	2	2	2	2
2	2	1	2	1	2	1	2	2
2	2	2	2	2	2	2	2	2
2	2	2	2	2	2	2	2	2
2	2	2	2	2	2	2	2	2
2	2	2	2	2	2	2	2	2
2	2	1	1	2	2	1	2	2
2	2	1	2	2	2	2	2	2
2	2	1	2	2	2	1	2	1

Neurosurgery	Other surgery	CVC	CVC removal (within 48h)	Other devices	Reanl Replacement Therapy	Any invasive procedures within	Parenteral Nutrition	Corticosteroid
2	2	1	2	1	2	2	1	2
2	2	2	2	1	2	2	1	2
1	2	1	2	1	2	2	1	1
2	2	1	2	1	2	1	1	1
2	2	1	2	1	2	2	1	2
2	2	1	2	2	2	2	2	2
2	2	1	1	1	2	2	1	2
2	2	1	2	1	2	2	1	1
2	2	1	2	1	2	2	1	2
2	2	1	2	1	2	2	1	2
2	2	1	2	1	2	2	1	1
2	2	1	2	1	2	2	1	2
2	2	1	1	1	1	1	2	1
2	2	1	2	1	2	1	2	2
1	2	2	2	1	2	2	1	1
2	2	1	2	1	2	2	1	2
1	2	1	2	1	2	2	1	1
2	2	1	2	1	2	2	1	2
2	2	1	2	1	2	2	1	2
2	2	1	2	2	2	2	1	2
1	2	1	2	1	2	2	1	2
1	2	1	2	1	2	2	2	1
2	2	1	2	1	2	1	1	2
2	2	1	2	2	2	1	2	2
2	2	1	2	2	2	2	2	2
2	1	2	2	1	2	2	2	2
2	2	1	2	1	1	1	1	2
2	2	1	2	1	2	2	1	1
2	2	1	2	1	2	2	1	1
2	2	1	2	1	2	2	2	2
2	2	1	2	2	2	2	1	1
2	2	2	2	1	2	2	1	2
2	2	1	1	1	2	2	1	1
2	2	1	2	1	2	1	2	2
2	2	1	2	1	2	2	1	2
2	2	1	2	1	2	1	2	2
2	2	1	2	1	2	2	1	2
2	2	1	1	1	2	2	1	2
2	2	1	1	1	1	2	1	1
2	1	1	1	1	2	2	2	2
2	2	1	2	1	2	2	1	2
2	2	1	2	2	2	2	1	2
2	2	1	2	1	2	2	2	1

2	2	2	2	2	2	2	2	1
2	2	1	2	1	2	1	2	2
2	2	1	2	2	2	2	1	2
2	2	1	2	1	2	2	2	2
2	2	1	2	1	2	1	1	2
2	2	1	1	1	2	1	1	2
2	2	1	2	1	2	2	2	2
1	2	1	2	1	2	2	1	2
2	2	1	1	1	2	1	1	1
2	2	1	2	1	1	2	2	2
2	2	1	2	1	2	1	2	1
2	2	1	2	1	2	2	1	2
2	2	1	2	1	2	1	1	1
2	2	1	2	1	2	1	1	1
2	2	1	2	1	2	2	1	2
2	2	1	2	2	2	2	1	2
2	2	1	2	1	2	1	1	2
2	1	1	2	1	2	2	2	2
2	2	1	1	1	2	1	1	2
2	2	2	2	1	2	2	2	2
2	2	1	2	1	2	1	2	2
2	2	1	1	2	2	2	2	2
2	2	1	1	1	2	2	2	2
2	2	1	2	1	2	2	1	2
2	2	1	2	1	2	1	1	2
2	2	1	2	1	2	2	1	2
2	2	1	2	1	2	1	1	2
1	2	1	2	1	2	1	1	2
2	2	1	2	1	2	2	1	2
2	2	1	2	1	2	1	1	2
2	2	1	1	1	2	2	1	2
2	2	1	1	1	2	2	1	2
2	2	2	2	1	2	2	1	2
2	2	2	2	1	2	1	2	2
2	2	1	2	1	2	2	1	2
2	2	1	2	1	2	1	2	1
2	2	1	2	1	2	2	1	1
2	2	1	2	1	2	2	1	2
2	2	1	1	1	1	1	1	2
2	2	1	1	1	1	2	1	2
2	1	1	2	1	2	2	1	2
2	2	2	2	1	2	2	2	2
2	2	1	2	1	2	2	1	2
2	2	1	2	2	2	2	1	2
2	2	1	2	2	2	2	1	2
2	2	1	2	1	2	2	2	2
2	2	1	2	1	2	2	1	1

1	2	1	2	1	2	2	1	2
2	2	1	2	1	2	2	2	1
1	2	1	2	1	2	1	2	2
2	2	1	2	1	2	2	1	2
2	2	1	1	1	2	1	1	2
2	2	1	2	1	1	1	1	2
2	2	1	2	1	2	2	1	2
2	1	1	1	1	2	2	2	1
1	2	1	2	1	2	1	1	2
2	2	1	2	1	2	2	2	2
2	2	1	2	1	2	2	2	2
2	2	2	2	2	2	2	2	2
1	2	1	2	1	2	2	2	2
2	1	2	2	1	2	2	2	2
2	2	1	2	1	2	2	1	2
2	2	1	2	1	2	2	2	2
2	2	1	2	1	2	2	1	2
2	2	1	2	1	2	2	2	1
2	2	1	2	1	2	2	1	2
2	2	1	1	1	2	2	1	2
2	2	1	2	1	2	2	1	2
2	2	1	1	1	2	1	1	2
2	2	1	2	1	2	1	1	1
2	1	2	2	1	2	2	2	2
2	1	1	2	1	2	1	2	1
2	2	2	2	2	2	2	1	1
2	2	2	2	1	1	2	1	2
2	2	1	2	1	2	2	1	2
2	2	1	2	1	2	1	2	1
2	1	1	2	1	2	2	1	2
2	2	2	2	1	2	2	1	2
2	2	1	1	2	2	2	1	2
2	2	1	1	2	2	2	1	2
2	2	1	2	1	2	2	2	1
2	2	1	1	1	1	2	1	2
2	2	1	2	1	2	1	1	2
2	2	1	2	1	1	2	2	2
2	2	2	2	1	2	2	2	2
2	2	1	2	1	2	1	1	2
2	2	1	2	1	2	1	1	1
2	2	2	2	1	2	2	1	2
2	2	1	2	1	2	1	1	2
2	2	1	2	1	2	2	2	2
2	2	2	2	1	2	1	1	2
2	2	1	2	1	2	1	1	2
2	1	1	1	1	2	2	1	2
2	1	1	2	1	1	2	2	1

2	2	1	2	1	2	2	1	2
2	2	1	2	2	2	2	2	2
2	1	2	2	1	2	1	2	2
2	2	1	1	1	2	1	1	2
2	1	1	2	1	1	2	2	1
2	2	1	2	1	2	2	1	2
2	2	1	2	1	2	1	1	2
2	1	1	2	2	2	2	1	1
2	2	1	2	1	2	2	1	2
2	2	2	2	2	2	1	1	2
1	2	1	2	1	2	2	2	2
1	2	1	1	1	2	2	2	2
2	2	1	2	1	1	1	1	2
2	2	1	2	1	2	2	1	2
2	2	2	2	2	2	1	1	2
1	2	1	2	1	2	2	2	2
2	2	1	2	1	2	2	1	2
2	2	1	2	1	2	2	1	2
1	2	1	2	1	2	2	2	2
2	1	1	2	1	2	2	1	1
1	2	1	2	1	2	2	2	2
2	2	2	2	1	2	2	1	1
2	2	1	1	1	2	2	1	2
2	1	1	2	1	1	2	2	1
2	2	1	2	1	2	2	2	2
2	2	2	2	1	2	2	1	2
2	2	1	1	1	2	2	1	2
2	2	1	1	1	2	2	1	2
2	2	1	2	1	2	1	1	2
2	2	1	2	1	2	2	1	2
2	2	1	1	1	1	2	2	2
2	2	1	1	1	2	2	2	2
2	2	1	1	1	2	2	1	1
2	2	1	2	1	2	2	2	2
2	1	1	1	1	2	2	2	2
2	1	1	2	1	2	1	2	2
2	2	1	1	1	2	1	1	2
2	2	1	2	1	2	1	2	2
2	1	2	2	2	1	2	2	1
1	2	1	2	1	1	2	1	2
2	2	1	2	1	2	2	2	2
2	2	1	2	1	2	2	2	2
2	2	1	2	1	1	1	1	2
2	2	1	2	1	2	1	2	2
2	2	1	2	1	2	2	1	1
2	2	1	1	1	2	2	1	2
2	2	1	2	1	1	1	1	1

2	2	1	1	1	2	2	1	2
2	2	1	2	1	2	2	2	1
2	2	1	2	1	2	1	1	2
2	2	1	1	1	2	1	1	2
2	2	1	2	1	2	2	1	1
2	2	1	2	1	2	1	1	2
2	2	1	1	1	2	2	1	1
2	1	1	2	1	2	2	1	2
2	2	1	2	1	2	2	2	2
2	2	1	2	1	1	1	1	1
2	2	1	1	1	1	2	1	2
2	2	1	2	1	2	1	2	2
2	2	1	2	1	1	1	1	2
2	2	1	2	1	2	2	1	2
2	1	1	2	1	2	2	1	2
2	2	1	2	1	2	2	2	1
2	2	1	2	1	2	2	2	2
2	2	1	1	1	2	2	1	2
1	2	1	2	1	2	1	2	1
2	2	1	1	1	2	2	2	2
2	2	1	2	1	2	2	2	2
2	2	1	2	1	2	2	1	1
2	2	1	2	1	2	2	1	2
2	2	1	2	1	2	2	1	1
1	2	1	2	1	2	2	2	2
2	2	1	2	1	2	2	2	2
2	2	1	1	1	2	2	1	2
1	1	1	2	1	2	1	1	2
2	2	1	2	1	2	2	1	2
2	2	1	2	1	2	2	2	2
2	2	1	2	1	2	1	1	2
2	2	1	2	1	2	1	1	2
2	1	1	2	1	2	2	2	1
2	2	1	2	1	2	1	2	2
2	1	2	2	1	2	1	2	2
2	2	1	2	1	2	2	1	2
1	2	1	2	1	2	1	1	2
2	1	1	2	1	2	1	2	2
1	2	1	2	1	2	2	1	2
2	2	1	2	1	2	1	1	1
2	2	1	2	2	2	2	2	2
2	2	1	2	1	2	1	1	2
2	2	1	1	2	2	2	1	2
2	2	1	2	1	2	1	2	2
2	2	1	2	1	2	2	2	1
2	2	1	2	2	2	1	2	1
2	2	1	2	1	1	1	1	1

2	2	1	1	1	2	2	1	1
2	2	1	2	1	2	1	1	1
1	2	1	1	1	2	2	1	2
2	2	1	2	1	2	2	1	2
2	2	1	1	1	2	2	2	2
2	2	1	2	1	1	1	1	2
2	1	1	2	1	2	2	1	2
2	2	1	2	1	2	2	1	2
2	2	1	2	1	2	2	1	1
2	2	1	2	1	2	2	1	1
2	2	1	1	1	2	2	1	2
2	2	2	2	1	2	2	2	1
2	2	1	2	1	2	2	1	2
2	2	1	1	1	2	2	1	2
2	2	1	2	1	2	2	1	1
2	2	1	2	1	2	1	2	1
2	1	1	1	1	2	2	2	2
2	2	1	2	1	2	2	1	2
2	2	1	2	1	2	1	1	1
2	2	2	2	2	2	2	2	2
2	1	1	2	1	2	2	2	2
2	1	1	2	1	2	2	2	2
2	2	1	2	1	2	2	2	2
2	2	1	2	1	2	1	1	2
2	2	1	2	1	2	2	1	2
2	2	1	2	1	2	2	1	1
2	2	1	2	2	2	2	2	1
2	2	1	2	1	2	2	1	2
2	2	1	1	1	2	2	1	1
2	1	1	2	1	2	2	1	2
1	2	1	2	1	2	2	1	1
2	2	1	2	1	2	1	1	2
2	2	2	2	1	2	1	2	2
2	2	1	2	1	2	2	1	2
2	1	2	2	1	2	2	2	2
2	2	1	2	1	2	1	1	1
2	2	1	2	1	2	2	2	1
2	2	1	2	1	2	2	1	1
2	2	1	2	2	2	2	1	2
2	2	1	2	1	2	2	2	2
2	2	1	2	1	1	1	1	2
2	2	1	2	1	2	2	2	2
2	2	1	2	1	2	1	1	2
2	2	1	2	1	2	1	2	2
2	2	1	2	1	2	1	1	2
2	2	1	2	1	2	2	1	2
2	2	2	2	2	2	1	1	1

2	2	1	2	1	2	2	1	1
2	2	1	2	2	2	2	2	2
2	2	1	1	1	2	2	1	2
2	2	2	2	1	2	2	1	2
2	2	2	2	2	2	2	1	2
2	2	1	2	1	2	2	1	1
2	2	1	2	1	2	2	2	1
2	2	2	2	2	2	1	2	2
2	2	1	2	2	2	2	2	1
2	2	1	1	1	2	1	1	2
2	2	2	1	1	2	2	1	2
1	2	1	1	1	2	1	2	1
2	2	2	2	1	2	1	1	2
2	1	1	2	1	2	2	2	2
2	2	1	2	1	1	2	2	2
2	1	1	1	1	2	2	1	1
2	2	1	1	1	2	2	1	1
2	2	1	2	1	2	2	1	2
2	2	1	2	1	2	1	1	2
2	2	1	2	1	2	1	2	2
2	2	1	2	1	2	2	1	1
2	2	1	1	1	2	2	1	2
2	2	1	2	1	2	2	1	2
2	2	1	2	1	2	2	1	1
2	2	1	2	1	2	2	1	1
2	1	1	2	1	2	1	1	2
2	2	1	1	1	1	2	1	1
2	2	1	2	1	2	2	1	1
2	1	2	2	1	2	2	1	2
2	2	1	2	2	2	2	1	2
2	2	1	2	1	1	1	1	2
2	2	1	2	1	2	2	1	2
2	1	1	2	1	2	2	1	1
2	2	1	2	1	2	1	1	2
2	2	1	2	1	2	2	2	1
2	2	2	2	1	2	2	1	2
2	1	1	2	1	2	1	1	2
2	2	2	2	1	2	1	1	1
2	2	1	1	1	2	2	1	2
2	2	1	2	1	2	1	1	2
1	2	1	1	1	2	2	1	2
1	2	1	2	1	2	2	2	1
2	1	2	2	1	2	1	2	2
2	2	1	2	1	2	2	1	1
2	2	2	2	1	2	1	1	1
2	2	1	2	1	2	2	1	1
2	1	1	2	1	2	2	1	2

2	2	1	2	1	2	1	1	2
2	2	2	2	1	2	2	1	2
2	2	1	2	1	2	2	1	2
2	2	1	1	1	2	1	1	1
2	2	1	1	1	2	2	1	2
2	2	1	2	2	2	2	1	1
2	2	1	2	1	2	2	2	2
2	2	1	2	2	2	2	2	1
2	2	2	2	1	2	1	1	2
2	1	1	2	1	1	2	1	2
2	2	1	2	1	2	1	1	1
2	2	1	1	1	2	1	1	2
2	2	2	2	1	2	2	1	1
1	2	1	1	1	2	2	2	1
2	1	1	2	1	1	2	2	1
2	2	1	2	1	2	2	2	2
2	2	1	2	1	2	2	1	2
2	2	1	2	1	2	2	1	2
2	2	1	2	1	1	1	1	1
2	1	1	1	1	2	2	2	1
2	2	1	2	1	2	2	1	1
2	2	1	2	1	2	2	1	1
2	2	1	2	1	2	2	1	2
2	1	1	1	1	2	2	1	2
2	2	1	2	1	2	1	2	2
2	2	1	2	1	2	2	1	2
2	2	1	2	1	2	2	1	2
1	2	1	2	1	2	1	1	2
2	2	1	2	1	2	2	1	1
2	2	1	2	1	2	1	1	2
2	1	1	2	1	2	1	1	2
2	2	1	2	1	2	1	2	2
2	2	1	1	1	2	2	2	2
2	2	1	2	2	2	2	1	2
2	2	1	1	1	1	2	1	2
2	2	1	2	1	1	1	1	2
1	1	1	2	1	2	1	2	2
1	1	1	2	1	2	1	2	2
2	2	1	2	1	1	2	1	1
2	2	1	2	1	2	2	1	1
2	2	1	1	1	2	2	1	2
2	2	1	1	1	2	2	1	1
2	2	1	2	1	2	2	1	1
2	2	1	2	1	2	2	2	2
2	1	2	2	1	2	2	1	1
2	1	2	2	1	2	2	2	1
2	2	1	2	1	2	2	2	2

2	2	1	2	1	2	2	1	1
2	2	1	1	1	2	2	1	1
2	2	1	2	1	2	2	1	1
2	2	1	2	1	2	1	1	2
2	2	1	1	1	2	2	2	2
2	2	1	2	1	2	1	1	1
2	2	1	2	1	2	2	2	1
2	2	1	2	1	2	2	2	1
2	1	1	2	1	2	2	2	2
2	2	1	2	1	2	2	1	1
2	2	2	2	2	2	1	2	2
2	1	1	1	1	2	1	2	2
2	2	1	2	1	2	1	1	2
2	2	1	2	1	2	1	1	1
1	2	1	2	1	2	2	1	2
2	1	1	2	1	2	2	2	2
2	2	2	2	1	2	1	2	1
2	2	1	1	1	2	2	1	2
2	2	1	2	1	2	1	2	2
2	2	1	2	1	2	1	1	2
2	1	1	2	1	1	1	1	2
2	2	1	2	2	2	2	2	2
2	2	2	2	1	2	1	1	2
2	2	1	2	1	2	1	1	2
2	2	2	2	1	2	1	1	1
2	2	2	2	2	2	2	2	2
2	2	1	2	1	2	2	1	2
2	2	1	2	1	2	2	1	2
2	2	1	1	1	2	2	1	2
2	2	1	2	1	2	2	1	2
2	2	1	2	2	2	2	1	1
2	2	1	2	1	2	2	1	1
2	2	1	2	1	2	2	2	1
1	2	1	2	1	2	2	1	1
2	2	1	2	1	2	1	2	1
2	2	1	2	1	2	1	2	1
2	1	1	2	2	2	2	2	1
2	1	1	1	1	1	1	1	2
1	2	1	2	1	2	2	2	2
1	2	1	1	1	2	1	2	1
1	2	1	2	1	2	2	2	2
2	2	1	2	1	2	1	1	2
2	2	1	1	1	2	1	2	2
2	1	1	1	1	2	2	2	2
2	2	1	2	1	1	1	1	1
2	2	1	1	1	1	2	1	2
2	2	2	2	2	2	2	2	2

2	2	1	1	1	2	2	1	2
2	2	1	2	2	2	2	2	2
2	2	1	2	1	2	2	2	1
2	2	1	2	1	2	1	1	2
2	1	1	2	1	2	2	2	2
2	2	1	1	2	1	2	2	2
2	2	1	2	1	2	2	1	2
2	2	1	2	1	2	2	2	2
2	2	1	1	1	2	2	2	2
2	2	1	2	2	2	1	2	2
2	2	1	1	1	1	2	1	2
2	2	1	2	2	2	2	1	1
2	2	1	1	1	2	2	2	2
2	2	1	2	1	1	2	1	2
2	2	1	2	1	1	1	1	2
2	2	1	1	1	2	2	1	2
2	2	1	2	1	2	1	2	2
2	2	1	2	2	1	2	2	2
2	1	1	2	2	2	2	2	2
2	2	1	2	1	2	2	1	1
2	2	1	1	2	2	2	2	2
2	2	1	1	1	2	2	1	1
2	2	1	2	1	2	2	1	2
2	2	2	2	1	2	2	2	2
1	2	1	1	1	2	2	2	1
1	2	1	2	1	2	2	2	2
1	2	1	2	1	2	2	2	2
2	2	1	1	1	2	2	1	1
2	2	1	2	1	2	2	1	2
2	2	1	1	1	2	2	1	2
2	2	1	2	1	2	2	1	1
2	2	1	1	1	2	2	1	2
2	2	1	2	1	2	2	1	2
2	2	1	2	1	2	2	1	2
2	2	1	1	1	2	2	2	2
2	2	1	2	1	1	2	2	1
2	2	1	2	1	2	2	1	2
2	2	1	1	1	2	2	1	2
2	1	1	2	2	2	2	1	2
2	1	1	2	2	2	2	1	2
2	2	1	1	1	2	1	1	1
2	2	1	2	1	2	2	2	1
2	2	1	1	1	2	2	1	2
1	2	1	2	1	2	2	2	2
2	2	1	2	1	2	2	2	1
2	2	1	2	2	2	2	1	2
2	2	1	2	1	2	2	1	2

2	2	1	2	1	2	2	1	2
2	2	1	1	1	2	2	2	2
2	2	1	1	1	2	2	2	2
2	2	1	1	1	2	2	2	2
2	2	1	1	1	2	2	2	2
2	2	1	1	1	2	2	1	2
2	2	1	2	1	2	2	2	1
2	2	1	2	1	2	2	2	2
2	2	1	1	1	2	2	1	2
2	2	2	2	2	2	2	1	1
2	2	1	2	1	2	1	2	2
2	2	2	2	2	2	2	2	2
2	2	1	2	1	2	1	2	2
2	2	2	2	2	2	2	2	2
2	2	1	2	2	2	2	1	2
2	2	1	2	1	2	1	2	2
2	2	1	2	2	2	1	1	1
2	2	1	2	2	2	1	1	1
2	2	1	1	1	2	2	2	2
1	2	1	2	1	2	2	1	1
2	2	1	2	2	2	2	1	2
2	2	1	1	2	2	2	2	2
2	2	1	2	2	2	2	2	2
2	2	1	2	1	2	1	1	2
2	2	1	2	1	2	2	1	2
2	2	1	2	2	2	2	2	2
2	2	2	2	1	2	1	1	1
2	2	1	2	2	2	1	1	2
2	2	1	2	2	2	2	1	2
2	2	1	2	1				
2	2	1	2	1	2	1	1	2
2	1	1	2	1	2	2	2	2
2	2	1	2	1	2	2	2	2
2	2	1	2	1	2	2	1	2
2	2	1	2	2	2	2	1	1
2	2	1	2	1	2	2	2	2
2	1	2	2	1	2	2	2	2
2	2	1	2	1	2	2	1	1
2	2	1	2	1	2	2	2	2

immunosoppressive therapies	Neutropenia	Acute diseases at the time of	Pneumonia	Intestinal perforation	Septic Shock	Deep Venous Thrombosis	Bleeding	Acute kidney failure
2	2	2	2	2	2	2	2	2
2	2	1	1	2	2	2	2	2
2	2	1	2	2	2	2	2	2
2	2	1	1	2	2	2	2	2
2	2	2	2	2	2	2	2	2
2	2	1	2	2	2	1	2	2
2	2	2	2	2	2	2	2	2
2	2	2	2	2	2	2	2	2
2	2	1	1	2	2	2	2	2
2	2	1	1	2	2	2	2	2
2	2	1	1	2	2	2	2	2
2	2	2	2	2	2	2	2	2
1	2	2	2	2	2	2	2	2
2	2	1	1	2	1	2	2	2
2	2	2	2	2	2	2	2	2
2	2	1	1	2	2	2	2	2
2	2	1	2	2	2	2	1	2
2	2	2	2	2	2	2	2	2
2	2	1	2	2	2	2	2	2
1	1	2	2	2	2	2	2	2
2	2	1	1	2	1	2	2	2
2	2	2	2	2	2	2	2	2
2	2	1	2	2	2	2	2	2
2	2	1	2	2	2	2	2	2
2	2	1	2	2	2	2	2	2
1	2	2	2	2	2	2	2	2
2	2	1	1	2	1	2	2	1
2	2	1	1	2	2	2	2	2
2	2	1	2	2	2	2	2	2
2	2	1	1	2	2	2	2	2
2	2	1	1	2	2	2	2	2
2	2	1	2	2	2	2	1	2
2	2	2	2	2	2	2	2	2
2	2	1	2	2	2	2	1	2
2	2	1	2	2	2	2	1	2
2	2	1	1	2	2	2	2	2
2	2	1	2	2	2	2	1	2
2	2	1	1	2	2	2	1	2
1	2	2	2	2	2	2	2	2
2	2	2	2	2	2	2	2	2
2	2	1	1	2	2	2	2	2
1	2	1	2	2	2	2	2	2
2	2	1	2	2	1	2	2	2

1	2	2	2	2	2	2	2	2
2	2	1	1	2	2	2	2	2
2	2	2	2	2	2	2	2	2
2	2	1	2	2	2	2	2	2
2	2	1	1	2	2	2	2	2
2	2	1	1	2	2	2	2	2
2	2	2	2	2	2	2	2	2
2	2	1	2	2	2	2	1	2
2	2	1	2	1	2	2	1	2
2	2	1	2	2	2	2	2	2
2	2	1	1	2	2	2	2	2
2	2	1	1	1	2	2	2	2
2	2	2	2	2	2	2	2	2
1	2	2	2	2	2	2	2	2
2	2	2	2	2	2	2	2	2
2	2	1	1	2	2	2	2	2
2	2	1	1	2	1	2	2	2
2	2	1	2	2	2	2	2	2
2	2	1	1	2	2	2	2	2
2	2	1	2	2	2	2	2	2
2	2	2	2	2	2	2	2	2
2	2	1	2	2	1	2	2	2
2	2	1	1	2	2	2	2	2
2	2	1	2	1	2	1	2	1
2	2	1	1	2	1	2	2	2
2	2	2	2	2	2	2	2	2
2	2	1	2	2	2	2	2	2
2	2	1	2	2	2	2	2	2
1	2	2	2	2	2	2	2	2
2	2	2	2	2	2	2	2	2
2	2	1	2	2	2	2	2	2
2	2	1	2	2	2	2	2	2
2	2	1	2	2	2	2	2	2
1	2	1	2	2	2	2	2	1
2	2	2	2	2	2	2	2	2
2	2	1	1	2	2	2	2	2
1	2	2	2	2	2	2	2	2
2	2	2	2	2	2	2	2	2
2	2	1	2	2	1	2	2	2
2	2	1	1	2	2	2	2	1
2	2	1	1	2	2	2	2	2
2	2	1	2	2	2	2	2	1
2	2	1	2	2	2	2	1	2
2	2	1	2	2	2	2	1	2
1	2	1	1	2	2	2	2	2
2	2	1	2	2	2	2	2	2
2	2	1	1	2	2	2	2	2

2	2	1	1	2	2	2	2	2
2	2	1	1	2	2	2	2	2
2	2	1	2	2	2	2	2	2
2	2	1	2	2	2	2	2	1
2	2	1	2	1	2	2	2	2
2	2	1	2	2	2	2	2	2
2	2	1	2	2	2	2	2	1
2	2	1	2	2	2	2	2	2
2	2	1	1	2	2	2	2	2
2	2	1	2	2	2	2	2	1
2	2	1	2	2	2	2	2	2
2	2	2	2	2	2	2	2	2
2	2	1	2	2	2	2	2	2
2	2	2	2	2	2	2	2	2
2	2	2	2	2	2	2	2	2
2	2	2	2	2	2	2	2	2
2	2	2	2	2	2	2	2	2
1	2	2	2	2	2	2	2	2
1	2	1	2	2	2	2	2	1
2	2	1	1	2	2	2	2	2
2	2	2	1	2	2	2	2	2
2	2	1	2	1	2	2	2	2
2	2	1	2	2	2	2	2	2
2	2	1	2	2	2	2	2	2
1	2	1	2	2	2	2	2	2
2	1	1	2	2	2	2	2	2
1	2	2	2	2	2	2	2	2
2	2	1	1	2	2	2	2	2
2	2	1	1	2	2	2	1	2
2	2	2	2	2	2	2	2	2
2	2	1	2	2	2	2	2	2
2	2	1	2	2	2	2	2	2
2	2	1	2	2	2	2	2	2
2	2	1	1	2	1	2	2	2
2	2	1	2	2	2	2	2	1
2	2	1	2	2	1	2	2	2
2	2	1	1	2	2	2	1	1
2	2	1	2	2	2	2	2	2
1	2	1	1	2	2	2	2	2
2	1	2	2	2	2	2	2	2
2	2	1	1	2	2	2	2	2
2	2	1	2	2	1	2	1	2
2	1	2	2	2	2	2	2	2
2	2	2	2	2	2	2	2	2
2	2	1	2	2	2	2	2	2
1	2	1	2	2	2	2	2	2
2	2	1	2	2	2	2	2	2

2	2	2	1	2	2	2	2	2
1	1	1	2	2	2	2	1	2
2	2	2	2	2	2	2	2	2
2	2	2	2	2	2	2	2	2
2	2	1	2	2	2	2	2	2
2	2	1	2	2	2	2	2	2
2	2	1	1	2	2	2	2	2
2	2	1	2	2	1	2	2	2
2	2	2	2	2	2	2	2	2
1	2	2	2	2	2	2	2	2
2	2	1	1	2	2	2	2	2
1	2	1	2	2	2	2	1	2
2	2	1	2	2	2	2	1	2
2	2	1	2	2	2	2	1	2
2	2	2	2	2	2	2	2	2
2	2	1	2	2	1	2	2	2
2	2	1	1	2	1	2	2	2
2	2	1	2	2	1	2	2	2
2	2	1	2	2	1	2	2	2
2	2	1	1	2	2	2	2	2
2	2	1	2	2	1	2	2	2
2	2	1	1	2	2	2	2	2
2	2	1	2	2	2	1	2	2
2	2	1	2	2	2	2	2	2
2	2	1	2	2	2	2	2	2
2	2	1	1	2	1	1	2	2
2	2	1	1	2	2	2	1	2
2	2	1	1	2	2	2	1	2
2	2	1	2	2	1	2	2	2
2	2	1	1	2	2	2	2	2
2	2	1	2	2	2	2	2	2
2	2	1	2	2	2	2	2	2
2	2	2	2	2	2	2	2	2
2	2	2	2	2	2	2	2	2
2	2	1	2	2	2	2	2	2
2	2	2	2	2	2	2	2	2
2	2	1	2	2	2	2	2	2
2	2	2	2	2	2	2	2	2
1	2	2	2	2	2	2	2	2
2	2	1	2	2	2	2	2	2
2	2	1	1	2	1	2	2	1
2	2	1	1	2	2	2	1	2
2	2	1	2	2	1	2	2	2
2	2	2	2	2	2	2	2	2
2	2	2	2	2	2	2	2	2
2	2	2	2	2	2	2	2	2
2	2	1	2	2	2	2	2	1

2	2	2	2	2	2	2	2	2
2	2	1	2	2	1	2	2	2
2	2	1	1	2	1	2	2	2
2	2	1	2	2	2	2	2	2
2	2	1	1	2	2	2	2	2
2	2	1	2	2	2	2	2	2
2	2	1	1	2	2	2	2	2
2	2	1	2	2	2	2	2	2
2	2	2	2	2	2	2	2	2
2	2	1	1	2	2	2	2	1
2	2	1	2	2	2	2	1	2
2	2	2	2	2	2	2	2	2
2	2	1	2	2	2	2	2	1
2	2	1	1	2	2	2	2	2
2	2	1	2	2	2	2	2	2
2	2	1	1	2	1	2	2	2
2	2	2	1	2	2	2	2	2
2	2	1	2	2	2	2	2	2
2	2	2	2	2	2	2	2	2
2	2	1	2	2	2	2	2	2
2	2	1	2	2	2	2	2	1
2	2	1	1	2	1	2	2	2
1	2	1	2	1	2	1	2	2
2	2	2	1	2	2	2	2	2
2	2	1	2	2	2	2	1	2
2	2	1	1	2	2	2	2	2
2	2	1	2	2	2	2	2	1
2	2	1	2	2	2	2	1	2
2	1	1	2	2	2	1	2	2
2	2	1	1	2	1	2	2	2
2	2	1	2	2	2	2	2	2
2	2	1	1	2	1	2	2	2
2	2	1	1	2	2	2	2	2
2	2	2	2	2	2	2	2	2
2	2	1	2	2	2	2	2	2
2	2	1	2	2	2	2	2	2
2	2	2	2	2	2	2	2	2
2	2	1	2	2	2	2	1	2
2	2	1	2	2	2	2	2	2
2	2	2	2	2	2	2	2	2
2	2	2	2	2	2	2	2	2
2	2	1	2	1	2	2	2	2
2	2	2	2	2	2	2	2	2
2	2	1	1	2	2	2	1	2
1	2	1	1	2	2	2	1	2
2	2	1	1	2	2	2	2	2
2	2	1	1	2	1	2	2	1

2	2	2	2	2	2	2	2	2
2	2	2	2	2	2	2	2	2
2	2	1	1	2	2	2	2	2
2	2	1	2	2	1	2	2	2
2	2	1	1	2	2	2	2	2
2	2	1	2	2	2	2	2	2
2	2	2	2	2	2	2	2	2
2	2	2	2	2	2	2	2	2
2	1	1	1	2	1	2	2	2
1	2	2	2	2	2	2	2	2
2	2	1	1	2	2	2	1	2
2	2	2	2	2	2	2	2	2
2	2	1	2	2	2	2	2	2
2	2	1	1	2	2	2	1	2
2	2	1	2	2	1	2	2	2
2	2	1	2	2	1	2	2	2
2	2	1	1	2	2	2	2	2
2	2	1	1	2	1	2	2	2
2	2	1	1	2	2	2	2	2
2	2	1	1	2	2	2	2	2
2	2	2	2	2	2	2	2	2
2	2	1	1	2	2	2	2	1
2	2	1	1	2	2	2	2	1
2	2	1	2	2	2	2	2	2
2	2	1	1	2	2	2	2	2
1	2	1	1	2	2	1	2	2
1	2	1	2	2	2	2	2	2
2	2	2	2	2	2	2	2	2
2	2	1	1	2	2	2	2	2
2	2	1	2	2	1	2	2	2
2	2	1	2	2	2	2	1	2
2	2	1	2	2	2	2	2	2
2	2	1	2	2	2	1	2	2
1	2	2	2	2	2	2	2	2
2	2	1	1	2	2	2	2	2
1	2	1	1	2	2	2	2	2
2	2	1	1	2	2	1	2	2
2	2	1	2	2	2	2	2	2
1	2	2	2	2	2	2	2	2
2	2	1	1	2	1	2	2	2
2	2	1	2	1	2	2	1	2
2	2	2	2	2	2	2	2	2
2	2	1	2	2	2	2	2	2
2	2	2	2	2	2	2	2	2
2	2	1	2	2	2	2	2	2
2	2	1	2	2	1	2	2	2
2	2	1	1	2	2	2	2	2

2	2	1	2	2	1	2	2	2
2	2	2	2	2	2	2	2	2
2	2	2	2	2	2	2	2	2
2	2	1	2	2	2	2	2	1
2	2	2	2	2	2	2	2	2
2	2	1	1	2	1	1	2	2
2	2	2	2	2	2	2	2	2
2	2	2	2	2	2	2	2	2
2	2	2	2	2	2	2	2	2
2	2	1	2	2	2	2	1	2
2	2	1	1	2	2	2	2	2
2	2	1	1	2	2	2	1	2
2	2	1	2	2	1	2	2	2
2	2	2	2	2	2	2	2	2
2	2	2	1	2	2	2	2	2
1	2	2	2	2	2	2	2	2
1	2	2	2	2	2	2	2	2
2	2	1	2	2	2	2	2	2
2	2	2	2	2	2	2	2	2
2	2	2	2	2	2	2	2	2
1	1	1	2	2	1	2	2	2
2	2	1	2	1	2	2	2	2
2	2	1	2	2	2	2	2	2
1	2	1	2	2	2	2	2	2
2	2	1	2	2	2	1	1	2
2	2	1	2	2	2	2	2	2
2	2	1	2	2	2	2	2	2
2	2	1	2	2	2	2	2	2
2	2	2	2	2	2	2	2	2
2	2	1	1	2	2	1	2	2
2	2	1	2	2	2	2	2	2
2	2	1	2	2	2	2	2	1
2	2	1	2	2	2	2	2	2
2	2	1	2	2	2	2	2	2
2	2	1	1	2	1	2	2	1
2	2	1	1	2	2	2	2	2
2	2	1	2	2	1	2	2	2
2	2	1	2	2	2	2	2	2
2	2	1	1	2	2	2	2	2
2	2	1	1	2	1	2	2	2
2	2	1	2	2	2	2	2	2
2	2	2	2	2	2	2	2	2
1	2	1	2	2	2	2	2	1
2	2	1	1	2	1	2	2	2
2	2	1	1	2	1	2	2	2
2	2	2	2	2	2	2	2	2
2	2	1	2	2	2	2	2	2

2	2	1	1	2	1	1	2	1
2	2	1	1	2	1	2	2	2
2	2	1	1	2	2	2	2	2
2	2	1	2	2	2	2	2	2
2	2	2	2	2	2	2	2	2
2	2	2	2	2	2	2	2	2
2	2	1	1	2	1	2	2	2
1	2	2	2	2	2	2	2	2
2	2	1	2	2	2	2	1	2
2	2	1	1	2	1	2	2	1
2	2	1	2	2	1	2	2	2
2	2	1	2	2	1	2	2	2
2	2	1	2	2	2	2	2	2
2	2	1	2	2	2	2	2	2
1	2	1	2	2	2	2	2	2
1	2	1	1	2	2	2	2	2
2	2	1	1	2	2	2	2	2
2	2	1	2	2	2	2	2	2
2	2	1	2	2	2	2	2	1
2	2	1	2	1	2	2	2	1
2	2	1	1	2	2	2	2	2
1	2	1	1	2	2	2	2	1
2	2	1	1	2	2	2	1	2
2	2	1	1	2	2	2	2	2
2	2	1	2	2	2	2	1	2
2	2	1	2	2	2	2	2	2
2	2	1	2	2	2	2	2	2
2	2	1	1	2	2	2	1	2
2	2	1	2	2	1	2	2	2
2	2	1	2	2	2	2	2	2
2	2	2	2	2	2	2	2	2
2	2	1	2	2	1	2	2	2
2	2	1	2	2	2	2	2	2
2	2	2	2	2	2	2	2	2
2	2	1	2	2	2	2	2	2
2	2	1	1	1	2	2	1	1
2	2	1	2	2	2	2	2	2
2	2	1	2	2	2	2	2	2
2	2	1	2	2	2	2	2	2
2	2	1	1	2	2	2	2	2
2	2	1	2	2	2	2	1	2
2	2	1	1	2	2	2	2	2
2	2	1	1	2	2	2	2	2
2	2	1	1	2	1	2	2	2
2	2	1	2	2	2	2	2	2
2	2	1	2	2	2	2	2	2
2	2	1	2	2	2	2	2	2

2	1	1	2	2	2	2	2	2
2	2	1	2	2	2	2	2	2
2	2	1	2	2	2	2	2	2
2	2	1	1	2	2	2	2	1
2	2	2	2	2	2	2	2	2
2	2	1	2	2	2	2	2	2
2	2	1	1	2	2	2	2	2
2	2	1	2	1	1	2	2	1
2	2	1	1	2	2	2	1	2
2	2	1	2	2	2	2	2	2
2	2	1	1	2	2	2	2	2
2	2	1	1	2	2	2	2	1
2	2	1	1	2	1	2	2	2
2	2	1	2	2	1	2	2	1
2	2	1	2	2	2	2	2	2
2	2	1	2	2	2	2	1	2
2	2	1	1	2	2	2	2	1
2	2	1	2	2	2	2	2	2
2	2	1	1	2	2	2	1	2
2	2	1	1	2	1	2	1	1
2	2	1	1	2	1	2	2	1
2	2	1	2	2	2	2	2	2
2	2	1	2	2	2	1	2	2
2	2	1	1	2	1	2	2	2
2	2	1	1	2	1	2	2	2
2	2	2	2	2	2	2	2	2
2	2	2	2	2	2	2	2	2
2	2	1	2	2	2	2	2	2
2	2	1	2	2	2	2	2	2
1	2	1	1	2	2	2	2	2
2	2	1	2	2	2	2	2	2
2	2	1	2	2	2	2	2	2
2	1	1	2	2	2	2	2	2
2	2	2	2	2	2	2	2	2
2	2	1	2	2	2	2	1	2
2	2	1	2	2	2	2	1	2
2	2	1	2	2	2	2	2	2
2	2	1	2	2	2	2	1	1
2	2	1	2	2	2	2	2	2
2	2	1	2	2	2	2	2	2
2	2	1	2	2	2	2	1	1
2	1	1	2	2	2	2	2	2
2	2	2	2	2	2	2	2	2
2	2	2	2	2	2	2	2	2
2	2	1	2	2	2	2	2	1
2	2	1	1	2	2	1	2	2
2	2	1	2	2	2	2	2	2

2	2	2	2	2	2	2	2	2
2	1	1	1	2	2	2	2	2
1	2	2	2	2	2	2	2	2
2	2	1	2	1	2	2	2	2
2	2	2	2	2	2	2	2	2
2	2	1	2	2	2	1	2	2
2	2	1	2	1	2	2	2	2
2	2	1	1	2	2	2	1	2
2	2	1	2	2	2	1	2	2
2	2	1	1	2	1	2	2	2
2	2	1	1	1	2	2	2	2
2	2	2	2	2	2	2	2	2
2	2	1	2	2	2	2	1	2
2	2	1	1	2	2	1	2	1
2	2	1	2	2	2	2	1	2
2	2	1	2	1	2	2	2	2
2	2	1	1	2	2	2	1	2
2	2	2	2	2	2	2	2	2
2	2	2	2	2	2	2	2	2
2	2	1	1	2	2	2	2	2
2	2	1	2	2	2	2	2	2
1	1	2	2	2	2	2	2	2
1	1	1	1	2	1	2	2	2
2	2	1	2	2	2	1	2	2
2	2	1	1	2	2	2	1	2
2	2	1	2	2	2	2	2	2
2	2	1	1	2	2	2	2	2
1	2	1	1	2	2	2	2	2
2	2	1	1	2	2	2	2	2
2	2	1	2	2	2	2	2	2
2	2	1	1	2	1	2	2	2
2	2	1	2	2	2	2	2	2
2	2	1	1	2	2	2	2	2
2	2	1	2	1	2	2	1	2
2	2	1	1	2	2	2	2	2
2	2	1	1	2	1	2	2	2
2	2	1	2	2	2	2	2	2
2	2	1	2	2	2	2	2	2
2	2	1	2	2	2	1	2	2
2	2	1	2	2	2	1	2	2
2	2	1	2	2	2	2	2	2
2	2	1	1	2	2	1	2	2
2	2	1	1	2	2	2	2	2
2	2	1	2	2	1	2	2	2
2	2	1	2	2	1	2	2	2
2	2	1	1	2	2	2	2	2
2	2	1	2	2	2	2	1	2

2	2	2	2	2	2	2	2	2
2	2	1	2	2	2	1	2	2
2	2	1	2	2	2	1	2	2
2	2	1	2	2	2	1	2	2
2	2	1	2	2	2	2	2	2
2	2	1	2	2	2	2	2	2
2	2	1	1	2	2	2	2	2
2	2	1	1	2	2	2	2	2
2	2	1	2	2	2	2	2	2
2	2	1	1	2	2	2	2	2
2	2	1	1	2	1	2	2	2
2	2	1	2	2	2	2	2	2
2	2	1	1	2	2	2	1	2
2	2	1	2	2	2	2	2	2
2	2	1	1	1	2	2	2	2
2	2	1	2	2	2	2	2	2
1	2	1	2	2	2	2	1	2
1	2	1	2	2	2	2	1	2
2	2	1	1	2	2	2	2	1
2	2	1	1	2	1	2	2	2
2	2	1	1	2	2	2	2	1
2	2	1	2	2	2	2	2	2
2	2	1	2	2	2	2	2	2
2	2	1	2	2	1	2	2	2
2	2	1	1	2	2	2	2	2
2	2	1	2	2	1	2	1	1
2	2	1	2	2	1	2	2	2
2	2	1	2	2	2	2	2	2
2	2	1	2	2	1	2	2	2
2	2	1	2	2	1	2	2	1
1	2	1	1	2	2	2	2	2
1	2	1	1	2	2	2	2	2
2	2	1	1	2	1	2	2	1
1	2	1	2	2	2	1	2	2
1	2	1	2	2	2	2	1	2
2	2	1	2	1	2	2	2	2
2	2	2	2	2	2	2	2	2
2	2	1	1	2	2	2	2	2

Trauma	Puolmonary embolism	Stroke	Acute Coronaric Syndrome	Decubitus ulcers	Use of betalactams at the time of	Other antibiotics	Use of Fluoroquinolones	total number of antibiotics before the
2	2	2	2	2	2	2	2	0
2	2	2	2	2	1	2	1	6
2	2	2	2	2	1	1	1	8
2	1	1	2	2	1	1	2	3
2	2	2	2	2	2	2	2	0
2	2	2	2	2	2	1	2	2
2	2	2	2	2	2	2	2	0
2	2	2	2	2	1	1	2	3
2	1	2	2	2	1	1	1	4
2	1	2	2	2	1	1	1	4
2	2	2	2	2	1	1	1	9
2	2	2	2	2	2	2	2	0
2	2	2	2	2	1	1	2	2
2	2	2	2	2	1	1	2	2
2	2	2	2	2	1	1	2	2
2	2	2	2	1	1	1	2	3
2	2	2	2	2	1	1	1	3
2	2	2	2	2	1	1	2	5
2	2	1	2	2	1	1	2	4
2	2	2	2	2	1	2	1	3
2	2	2	2	2	2	1	2	1
2	2	2	2	2	1	2	1	2
2	1	2	2	2	1	1	2	5
2	2	2	2	2	1	1	1	6
2	2	2	2	2	1	1	2	5
2	2	2	2	2	1	2	2	1
2	2	2	1	2	1	1	1	6
2	1	2	2	2	1	1	2	3
2	2	2	1	1	1	1	2	5
2	2	2	2	2	2	2	2	0
2	2	2	2	2	1	1	2	2
2	2	2	2	2	1	1	2	3
2	2	2	2	2	2	1	1	6
2	2	2	2	2	2	2	2	0
2	2	2	2	2	1	1	1	7
2	2	2	2	2	2	2	2	0
2	2	2	2	2	1	1	2	4
2	2	2	2	2	2	1	1	2
2	2	2	2	2	1	1	1	8
2	2	2	2	2	1	1	2	4
2	2	2	2	2	1	1	2	6
2	2	2	2	2	2	2	2	0
2	2	2	2	2	1	1	2	2

2	2	2	2	2	1	2	1	2
2	2	2	2	2	1	1	2	3
2	2	2	2	2	1	1	1	7
1	2	2	2	1	1	2	1	2
2	2	2	2	1	1	1	2	4
2	2	2	2	2	1	1	1	7
2	2	2	2	2	1	1	2	3
2	2	2	2	2	1	1	1	7
2	2	1	2	2	1	1	1	3
2	2	2	2	2	1	1	2	6
2	2	2	2	1	1	1	1	7
2	1	2	2	2	1	1	1	6
2	2	2	2	2	1	1	2	3
2	2	2	2	2	1	1	2	3
2	2	2	2	2	2	2	2	0
2	2	2	2	2	1	1	2	5
2	2	2	2	1	1	2	1	8
1	2	2	2	1	1	1	2	4
2	2	2	2	2	2	1	2	1
2	2	1	2	2	1	2	1	3
2	2	2	2	2	1	1	1	6
2	2	2	2	2	1	1	2	2
2	2	2	2	2	2	2	2	0
2	2	2	2	2	1	1	1	4
2	2	2	2	1	1	1	1	12
2	2	2	2	2	2	2	2	0
2	2	2	1	2	1	2	1	2
1	2	2	2	2	1	1	1	6
2	2	2	2	2	2	1	1	3
2	2	2	2	2	1	1	2	4
2	2	2	2	2	2	2	2	0
2	2	2	2	2	1	1	2	3
2	2	2	2	2	2	2	2	0
2	2	2	2	2	2	1	2	2
2	2	2	2	2	2	2	2	0
2	1	2	2	2	1	1	2	2
2	2	2	2	2	1	1	1	3
2	2	2	2	2	1	2	2	1
2	2	2	1	2	1	1	1	3
2	2	2	2	2	1	1	1	5
1	2	2	2	2	2	2	2	3
2	2	2	2	2	1	2	1	2
2	2	2	2	2	1	1	1	5
2	2	2	2	2	2	2	1	1
2	2	2	2	2	1	1	2	6
2	2	2	2	1	1	1	1	3
2	2	2	2	2	1	1	2	6

2	2	2	2	2	1	1	2	2
2	2	2	2	2	2	2	2	0
1	2	2	2	2	2	2	2	1
2	2	2	2	2	1	1	1	8
2	2	2	2	2	1	1	1	3
2	2	2	2	2	1	2	1	3
1	2	2	2	2	2	1	2	4
1	2	2	2	2	1	1	2	3
1	2	2	2	2	1	2	2	1
2	2	2	2	2	1	1	1	3
2	2	2	2	1	1	1	2	4
2	2	2	2	2	2	1	2	3
2	2	2	2	2	1	2	2	1
2	2	2	2	2	2	2	2	0
2	2	2	2	2	1	1	2	2
2	2	2	2	2	2	2	2	0
2	2	2	2	2	1	1	2	2
2	2	2	2	2	1	1	1	5
2	2	2	2	2	1	1	2	5
2	1	2	2	2	2	1	1	2
2	2	2	2	2	1	1	1	7
2	2	2	2	2	1	1	2	4
2	2	2	2	2	1	1	1	4
1	2	2	2	2	2	2	2	0
2	2	2	2	2	2	1	2	3
2	2	2	2	2	2	1	1	2
2	2	2	2	2	1	1	1	3
2	2	2	2	2	1	1	1	4
2	2	2	2	2	1	2	1	3
2	2	2	2	2	1	1	2	2
2	2	2	2	1	1	1	2	2
2	2	2	2	2	1	1	1	6
2	2	2	2	2	1	1	1	6
2	2	2	2	2	1	1	2	2
2	2	2	1	2	1	1	1	3
2	2	2	2	2	1	1	1	6
2	2	2	2	2	1	1	1	9
2	2	2	2	2	2	2	2	0
2	2	2	2	2	2	2	2	0
2	2	2	2	2	2	2	2	0
2	2	2	2	2	2	2	2	0
2	2	2	2	2	1	1	1	3
2	2	2	2	2	1	2	2	1
2	2	2	2	2	2	2	2	0
2	2	2	2	2	1	1	1	3
2	2	2	2	2	1	1	1	5
2	2	2	1	1	1	1	1	7

1	2	2	2	2	1	1	1	3
2	2	2	2	2	1	1	1	5
2	2	2	2	2	1	1	1	4
2	2	2	2	2	1	1	2	2
2	2	2	1	1	1	1	1	7
2	2	2	2	2	2	1	1	4
2	2	2	2	2	2	1	1	2
2	2	2	2	2	1	1	2	2
2	2	2	2	2	1	1	2	3
2	2	2	2	2	2	2	2	0
2	2	2	2	2	1	1	1	7
2	2	1	2	2	1	2	2	3
2	2	2	2	2	1	2	1	4
1	2	2	2	2	2	2	2	0
2	2	2	2	2	1	1	2	2
2	2	2	2	2	2	2	1	1
2	2	2	2	2	1	1	1	6
2	2	2	2	2	2	1	1	6
2	2	2	2	2	2	2	1	1
2	1	2	2	2	1	1	1	6
2	2	2	2	2	2	2	1	1
2	2	2	2	1	1	1	2	4
2	2	2	2	2	2	2	2	0
2	2	2	1	1	1	1	1	7
2	2	2	2	2	1	1	2	3
2	2	2	2	2	2	1	2	1
2	2	2	2	2	1	1	1	10
2	2	2	2	2	1	1	1	10
1	2	2	2	1	1	1	2	2
1	2	2	2	1	2	2	2	0
2	2	2	2	2	1	2	1	2
2	2	2	2	2	2	2	1	1
2	2	2	2	2	1	1	2	2
2	2	2	2	2	1	2	2	1
1	2	2	2	2	1	1	2	5
2	2	2	2	2	2	2	1	1
2	2	2	2	2	1	1	1	3
2	2	2	2	2	2	2	1	1
2	2	2	2	2	1	2	2	1
2	2	2	1	2	1	1	2	3
2	2	2	1	2	1	1	2	2
1	2	2	2	2	1	1	1	3
2	2	2	2	2	1	1	1	8
2	2	2	2	2	2	2	2	0
2	2	2	2	2	1	2	2	1
2	2	2	2	2	1	1	1	9
2	2	2	2	1	1	2	2	1

2	2	2	2	2	2	2	2	0
2	2	2	2	2	1	1	2	4
2	2	2	2	2	1	1	1	4
2	2	2	1	2	1	1	1	8
2	2	2	2	1	1	1	2	8
2	2	2	2	2	1	1	1	4
2	2	2	2	2	1	1	2	2
2	2	2	2	1	1	1	2	3
2	2	2	2	2	2	1	2	1
2	2	2	2	2	1	1	1	6
2	2	2	2	2	1	2	2	1
2	2	2	2	2	2	2	2	1
2	2	2	2	2	1	2	2	2
1	2	2	2	2	2	2	1	1
2	2	2	2	2	2	2	2	0
2	2	2	2	1	1	1	1	8
2	2	1	2	2	1	1	2	4
1	2	2	2	1	1	1	1	8
2	2	2	2	2	1	2	2	3
2	2	2	2	1	2	2	2	0
2	2	2	2	2	1	1	2	4
2	2	2	2	2	1	1	1	3
2	2	2	2	2	1	2	2	1
2	2	2	2	2	1	1	2	2
2	2	2	2	2	1	1	2	4
2	2	2	2	2	1	1	2	5
2	2	2	2	2	1	2	1	3
1	2	2	2	2	1	1	1	3
2	2	2	2	1	1	1	2	2
2	2	2	2	1	1	1	2	3
2	2	2	2	1	1	1	1	6
2	2	2	2	2	1	1	2	4
1	1	2	2	2	1	1	1	7
2	2	2	2	2	2	2	1	2
2	1	2	2	2	1	2	2	5
2	1	2	2	2	2	2	2	0
2	2	2	2	2	1	1	1	4
1	2	2	2	2	1	1	1	4
2	2	2	2	2	2	2	2	0
2	2	2	2	2	1	1	1	3
2	2	2	2	2	2	2	2	0
2	2	2	2	2	1	1	2	3
2	2	2	2	2	2	2	1	1
2	2	2	2	1	2	1	2	1
2	2	2	2	2	2	1	2	2
2	2	2	2	2	1	1	1	7
2	2	2	1	2	2	1	1	2

2	2	2	2	2	1	1	2	4
2	2	2	2	2	1	1	1	3
2	2	2	2	2	1	1	1	3
2	2	2	2	2	1	1	1	3
2	2	2	2	2	1	1	1	6
2	2	2	2	2	2	1	1	2
2	2	2	2	2	1	1	2	4
2	2	2	2	2	2	2	1	1
2	2	2	2	2	1	1	2	3
2	2	2	2	2	1	1	1	3
2	2	2	2	2	1	1	1	5
2	2	2	2	2	2	1	2	2
2	1	2	2	2	1	1	2	2
2	2	2	2	2	1	1	1	5
2	2	2	2	2	1	1	1	9
2	1	2	2	2	1	1	2	2
1	2	2	2	2	1	1	1	6
2	2	2	2	2	1	1	2	4
2	2	2	2	2	2	2	2	0
2	2	2	2	2	1	1	1	3
2	2	2	2	2	2	2	2	0
2	2	2	2	1	1	1	2	4
2	2	2	2	2	1	2	2	1
2	2	2	1	2	1	1	2	4
2	2	2	2	2	2	2	2	0
2	2	2	2	2	1	1	1	5
2	2	2	1	2	2	2	1	1
2	2	2	2	2	1	1	2	2
1	2	2	2	2	1	1	1	4
2	2	2	2	2	1	1	2	3
2	2	2	2	2	1	1	2	4
2	2	2	2	2	1	1	2	2
2	2	2	2	2	2	2	1	1
2	2	2	2	2	2	2	2	0
2	2	2	2	2	1	1	2	5
2	2	2	2	2	1	2	1	2
2	2	2	2	2	1	1	1	4
2	2	1	2	2	1	1	2	3
2	2	2	2	2	2	2	2	0
2	2	2	2	2	1	1	1	3
2	2	2	2	2	1	1	1	7
2	2	2	2	2	2	2	2	0
2	2	2	2	2	1	1	2	4
2	2	2	2	2	1	1	2	2
2	2	2	2	2	2	1	1	2
2	2	2	2	2	1	1	1	5
2	2	2	1	2	1	1	2	3

2	2	2	2	2	1	1	2	5
2	2	2	2	2	1	2	1	2
2	2	2	2	2	1	2	2	2
2	2	2	2	1	1	2	2	1
2	2	2	2	2	1	2	2	1
2	2	1	2	2	1	1	1	3
2	2	2	2	2	1	1	2	2
2	2	2	2	2	1	1	2	2
2	2	2	2	2	1	1	2	2
1	2	2	2	2	1	1	1	8
2	2	2	2	2	2	2	2	0
2	2	2	2	2	1	1	2	4
2	2	2	2	2	1	1	2	2
2	2	2	2	2	1	1	1	3
2	2	2	2	2	1	2	2	1
2	2	2	2	2	1	1	1	3
2	2	2	2	2	1	1	2	3
2	2	2	2	1	1	1	2	2
2	2	2	2	2	1	1	1	3
2	2	2	2	2	1	1	2	4
2	2	2	2	2	1	1	1	3
2	2	2	2	2	1	1	2	2
2	2	2	2	1	1	1	1	5
2	2	2	2	2	2	2	2	0
2	2	2	2	2	1	1	2	3
2	2	2	2	2	1	1	1	5
2	2	2	2	1	1	1	1	7
2	2	2	2	1	1	1	1	8
2	2	2	2	2	1	2	1	3
2	2	2	2	2	1	2	1	4
2	2	2	2	2	1	1	2	2
2	2	2	2	2	1	1	2	2
2	2	2	2	2	2	2	1	1
2	2	2	2	2	1	1	1	10
2	2	2	2	2	1	1	2	4
2	2	2	2	2	2	2	2	0
2	2	2	2	2	1	1	1	3
2	2	2	2	2	1	1	2	2
2	2	2	2	2	1	1	2	2
2	2	2	2	2	1	1	2	2
2	2	2	2	2	1	1	2	5
2	2	2	2	2	1	1	1	5
2	2	2	2	2	1	1	2	2
2	2	2	2	2	1	2	2	2
2	2	2	2	2	1	1	2	2
2	2	2	2	2	1	2	2	2
2	2	2	2	2	1	1	1	4

2	2	2	2	1	1	1	2	3
2	2	2	2	2	1	2	1	4
2	2	2	2	2	2	2	2	0
2	2	2	2	2	1	1	1	3
2	2	2	2	2	2	2	1	1
2	2	2	2	2	2	2	2	1
2	2	2	2	2	1	1	2	2
2	2	2	2	2	2	2	2	0
2	2	2	2	2	1	1	2	2
2	2	2	2	2	1	1	2	4
2	2	2	2	1	1	1	2	5
2	2	2	2	2	2	2	2	0
2	2	2	1	2	2	2	1	2
1	2	2	2	2	1	1	1	7
2	2	2	2	2	1	1	1	3
2	2	2	2	2	1	1	2	2
2	2	2	2	2	1	1	1	3
2	2	2	2	2	1	1	2	4
2	2	2	2	2	1	1	1	4
2	2	2	2	2	1	1	1	5
2	2	2	2	2	1	2	1	2
2	2	2	2	2	2	1	2	1
2	2	2	2	2	1	1	2	4
2	2	2	2	2	1	2	2	2
2	2	2	2	2	2	2	1	1
2	2	2	2	2	1	1	1	3
2	2	2	2	2	1	1	2	3
1	2	2	2	2	1	1	1	3
2	2	2	2	2	1	1	2	4
2	2	2	2	2	1	1	2	4
2	2	2	2	2	1	1	2	3
2	2	2	2	2	1	1	1	10
2	2	2	1	1	1	1	1	4
2	2	2	2	2	2	2	1	1
2	2	2	2	2	1	1	1	5
2	2	2	2	2	1	1	1	6
2	2	2	2	2	1	1	2	4
2	2	2	2	2	1	1	2	4
2	2	2	1	2	1	1	2	4
2	2	2	1	1	1	2	1	0
2	2	2	2	1	1	1	2	4
2	2	2	1	2	1	1	1	6
2	2	2	2	1	1	1	2	3
2	2	2	2	2	2	1	2	3
2	2	2	2	2	1	1	2	4
2	2	2	2	2	1	1	1	4
2	2	2	2	2	1	2	2	2

2	2	2	2	2	1	1	1	7
2	2	2	2	2	1	1	2	3
2	2	2	2	2	1	1	2	3
2	2	2	2	1	1	1	2	4
2	2	2	2	2	1	2	2	1
1	2	2	2	2	1	1	1	6
2	2	2	2	1	1	1	2	4
2	2	2	2	2	1	1	2	3
2	2	2	2	1	1	1	2	2
2	2	2	2	1	1	1	2	5
2	2	2	2	2	1	1	2	2
2	2	2	2	2	1	1	2	2
2	2	2	2	1	1	1	2	2
2	2	2	2	2	1	1	2	2
2	2	2	2	2	1	1	1	3
1	2	2	2	2	1	1	1	3
2	2	2	1	1	1	1	2	2
2	1	2	2	2	1	1	2	2
2	2	2	2	2	2	2	2	0
2	2	2	2	2	1	2	1	2
2	2	2	2	2	1	1	2	2
1	2	2	2	2	1	1	1	3
2	2	2	2	2	1	1	2	2
1	2	2	2	2	1	1	2	3
2	2	2	2	2	1	1	1	9
2	2	2	2	2	1	1	2	2
2	2	2	2	2	1	2	2	1
2	2	2	2	1	1	1	2	3
2	2	2	2	2	1	1	2	3
2	2	2	2	1	1	2	2	2
2	2	2	2	2	1	2	2	1
2	1	2	2	2	1	2	2	1
1	2	2	2	2	1	1	2	5
2	2	2	2	2	1	1	2	2
2	2	2	2	1	1	1	2	2
2	2	2	2	1	1	1	2	2
1	2	2	2	2	1	2	1	2
1	2	2	2	2	1	1	2	4
1	2	2	2	2	1	2	2	1
2	2	2	2	1	1	1	1	8
2	2	2	2	2	1	1	2	2
2	2	2	2	2	1	1	2	4
2	2	2	2	2	1	1	1	3
2	2	2	2	2	2	2	1	1
2	2	2	2	1	1	1	1	7
2	2	2	2	2	1	2	1	4
2	2	1	2	2	1	1	2	3

2	2	2	2	2	2	2	2	0
2	2	2	2	2	2	2	2	no
2	2	2	2	2	1	1	2	3
2	1	2	2	2	1	1	2	3
2	2	2	2	2	1	1	2	3
2	1	2	2	2	1	1	1	6
2	2	2	2	2	2	2	2	no
2	2	1	2	2	1	1	2	4
2	2	2	2	2	1	2	2	2
2	2	2	2	2	1	1	2	2
2	2	2	2	2	1	2	1	2
2	2	2	2	2	2	1	1	2
2	2	2	2	2	1	1	1	4
2	2	2	2	2	1	1	1	7
2	2	2	2	2	1	1	2	4
2	2	2	2	2	1	1	2	2
2	2	2	2	2	1	1	1	5
2	2	2	2	2	2	2	2	0
2	2	2	2	2	1	2	2	1
2	2	2	2	2	1	2	2	3
2	2	2	2	2	1	1	1	5
2	2	2	2	2	1	1	2	3
2	2	2	2	2	1	1	2	2
2	2	2	2	2	1	1	1	5
2	2	2	2	2	1	1	2	2
1	2	2	2	2	1	2	2	2
2	2	2	2	2	1	1	1	5
2	2	2	2	1	1	1	1	5
1	1	2	2	2	1	1	1	6
2	2	2	2	2	1	1	2	3
2	2	2	2	2	1	1	2	3
2	2	2	2	2	1	1	2	3
2	2	2	2	2	1	2	2	2
2	2	2	2	2	1	1	1	4
2	2	2	2	2	1	1	2	7
2	2	2	2	2	1	1	1	6
2	2	2	2	2	1	1	2	2
2	2	2	2	2	1	1	2	2
2	2	2	2	1	1	1	2	4
2	2	2	2	1	1	1	2	4
2	2	1	2	2	1	1	2	4
2	2	1	2	2	1	1	2	4
2	2	1	2	2	1	1	2	6
2	1	2	2	2	1	1	2	4
2	2	2	2	2	1	1	2	3
2	2	1	2	2	2	2	2	0
2	2	2	2	2	1	1	1	3

2	2	2	2	2	2	2	2	0
2	2	2	2	2	1	1	2	4
2	2	2	2	2	1	1	2	4
2	2	2	2	2	1	1	2	
2	2	2	2	2	1	1	1	5
2	2	2	2	1	1	1	2	5
2	2	2	2	2	1	2	1	2
2	2	2	2	2	1	1	2	2
2	2	2	2	2	1	1	2	3
2	2	2	2	2	1	1	2	3
2	2	2	2	2	1	1	2	5
2	2	2	2	2	1	2	2	3
2	2	2	2	1	1	1	2	2
2	2	1	2	2	1	1	2	2
2	1	2	2	2	1	1	2	3
2	2	2	2	2	1	2	2	1
2	2	2	2	2	1	1	2	3
2	2	2	2	2	1	1	2	4
2	2	2	1	2	2	2	2	0
2	2	2	2	2	1	1	2	4
2	2	2	2	2	1	1	2	3
2	2	2	2	2	2	2	1	1
2	2	2	2	2	2	2	1	1
2	1	2	2	2	1	1	2	3
2	2	2	2	2	1	2	2	1
2	2	2	2	2	1	1	1	3
2	2	2	2	2	1	1	2	4
2	2	2	2	2	1	1	2	3
2	2	2	2	1	1	1	2	4
2	2	2	2	2	2	1	2	3
2	2	2	2	2	1	1	2	4
2	2	2	2	2	1	1	1	4
2	2	2	2	2	1	1	2	3
2	2	2	2	2	1	2	2	2
2	2	2	2	2	1	1	2	2
2	2	2	2	2	2	1	2	4
2	2	2	2	2	2	2	2	0
2	2	2	2	2	1	1	2	3

Candida species	BSI CVC related	Duration of Candidemia	Main antifungal therapy	**Appropiateness of main antifungal**	Antifugal/s administered before	Azoles	Echinocandins	Contemporary positive blood
2	2	1	4	**2**	2	2	2	2
2	2	10	2	**1**	2	2	2	1
2	2	24	1	**1**	2	2	2	2
4	1	18	1	**2**	2	2	2	2
1	1	1	2	**1**	2	2	2	2
5	2	4	2	**2**	2	2	2	2
4	1	17	1	**2**	2	2	1	1
5	1	1	1	**1**	1	1	1	1
2	1	1	1	**1**	2	2	2	2
2	1	1	4	**2**	2	2	2	2
4	2	1	4	**2**	1	1	2	2
1	2	1	4	**2**	2	2	2	2
1	2	2	2	**2**	2	2	2	2
1	1	1	4	**2**	2	2	2	2
3	2	1	2	**1**	2	2	2	2
2	2	7	2	**1**	2	2	2	1
2	1	35	2	**1**	2	2	2	1
1	1	1	4	**2**	2	2	2	1
1	1	1	4	**2**	2	2	2	1
1	2	1	4	**2**	2	2	2	2
3	1	1	1	**1**	2	2	2	2
1	2	1	4	**2**	2	2	2	1
1	2	1	2	**1**	2	2	2	1
1	1	6	1	**2**	2	2	2	1
3	2	3	1	**1**	2	2	2	1
1	2	1	2	**2**	2	2	2	2
1	1	2	2	**2**	2	2	2	2
1	1	7	1	**1**	2	2	2	1
2	1	9	2	**2**	2	2	2	1
1	2	1	2	**1**	2	2	2	1
1	1	2	2	**1**	2	2	2	2
1	2	1	4	**2**	2	2	2	2
2	1	1	1	**1**	2	2	2	1
1	2	1	4	**2**	2	2	2	1
1	2	1	4	**2**	2	2	2	1
1	1	1	4	**2**	2	2	2	2
1	1	6	2	**1**	2	2	2	2
1	1	1	1	**1**	2	2	2	2
1	1	8	1	**1**	2	2	2	1
2	2	2	2	**1**	2	2	2	2
1	2	1	2	**2**	2	2	2	2
2	1	10	4	**2**	2	2	2	2
1	1	1	2	**1**	2	2	2	1

2	2	1	4	**2**	2	2	2	2
1	1	1	2	**1**	2	2	2	1
1	2	1	2	**1**	2	2	2	2
1	1	2	2	**1**	2	2	2	2
3	2	1	2	**1**	2	2	2	1
3	1	83	1	**1**	1	2	1	2
4	2	1	4	**2**	2	2	2	1
2	2	16	3	**1**	2	2	2	2
2	1	1	4	**2**	1	2	1	1
2	2	10	1	**1**	2	2	2	1
1	1	1	2	**1**	2	2	2	2
4	1	1	2	**1**	2	2	2	1
4	2	1	4	**2**	2	2	2	2
1	2	2	4	**2**	2	2	2	2
5	1	1	4	**2**	2	2	2	2
2	2	1	2	**1**	2	2	2	2
1	1	7	1	**2**	2	2	2	2
5	1	1	2	**1**	1	1	2	2
3	1	2	3	**1**	2	2	2	1
1	2	9	4	**2**	2	2	2	2
4	1	19	1	**2**	2	2	2	2
2	1	1	4	**2**	2	2	2	1
3	1	1	4	**2**	2	2	2	1
2	1	1	2	**1**	1	1	2	2
2	1	7	1	**1**	1	2	1	1
2	1	7	2	**1**	2	2	2	2
2	1	3	1	**1**	2	2	2	2
1	2	7	2	**1**	2	2	2	2
1	1	9	1	**1**	2	2	2	2
4	1	10	1	**1**	2	2	2	1
1	1	11	4	**2**	2	2	2	2
1	1	12	1	**2**	2	2	2	2
1	2	1	1	**2**	2	2	2	2
1	2	1	1	**1**	2	2	2	2
1	1	1	2	**1**	2	2	2	2
1	2	1	1	**1**	2	2	2	2
2	1	8	2	**2**	2	2	2	1
1	1	9	4	**2**	2	2	2	2
1	2	7	2	**2**	2	2	2	1
1	1	4	2	**1**	2	2	2	2
1	1	1	2	**1**	2	2	2	2
1	2	1	4	**2**	2	2	2	2
1	1	26	2	**1**	1	1	2	1
2	1	38	2	**2**	2	2	2	1
1	1	1	2	**1**	2	2	2	1
2	1	4	2	**1**	2	2	2	2
1	1	1	2	**1**	2	2	2	2

1	1	1	1	**1**	2	2	2	2
3	2	1	4	**2**	2	2	2	2
2	2	1	4	**2**	2	2	2	1
4	1	1	1	**1**	1	1	1	2
2	2	1	4	**2**	2	2	2	2
1	2	3	1	**1**	2	2	2	1
4	1	1	2	**2**	2	2	2	2
2	1	1	2	**1**	2	2	2	2
1	1	1	1	**1**	2	2	2	2
3	1	1	4	**2**	2	2	2	2
2	2	7	2	**2**	2	2	2	2
1	2	21	3	**1**	2	2	2	2
1	1	2	4	**2**	2	2	2	2
2	2	1	4	**2**	2	2	2	2
1	2	1	2	**1**	2	2	2	1
1	1	2	1	**1**	2	2	2	2
4	2	1	2	**1**	2	2	2	1
4	2	1	2	**1**	2	2	2	1
2	1	1	1	**1**	2	2	2	1
1	1	17	1	**2**	2	2	2	2
1	1	1	2	**1**	1	1	2	2
1	2	1	2	**1**	2	2	2	1
1	1	1	2	**2**	2	2	2	2
2	2	1	4	**2**	2	2	2	2
1	1	7	1	**1**	2	2	2	1
1	2	1	4	**2**	2	2	2	2
2	2	1	2	**1**	2	2	2	2
1	1	1	2	**1**	2	2	2	2
2	1	1	2	**1**	2	2	2	2
3	1	1	1	**1**	2	2	2	2
5	2	12	2	**1**	2	2	2	2
3	1	5	1	**1**	2	2	2	2
1	1	1	1	**1**	2	2	2	2
5	2	1	1	**2**	2	2	2	2
1	1	1	4	**2**	2	2	2	2
1	2	10	2	**1**	2	2	2	2
1	1	7	1	**1**	2	2	2	2
4	2	1	4	**2**	2	2	2	2
1	2	19	2	**1**	1	1	2	1
2	1	1	4	**2**	2	2	2	1
2	2	29	1	**1**	1	2	1	2
5	1	1	2	**1**	2	2	2	1
2	2	14	1	**1**	2	2	2	1
2	2	53	2	**1**	2	2	2	2
1	2	1	4	**2**	2	2	2	1
1	1	5	1	**1**	2	2	2	2
4	1	1	1	**2**	2	2	2	2

2	1	7	2	**2**	2	2	2	1
5	1	7	1	**2**	2	2	2	2
1	2	1	2	**1**	2	2	2	1
1	2	1	4	**2**	2	2	2	1
4	1	1	1	**1**	1	2	1	1
1	1	5	2	**1**	2	2	2	2
1	2	1	2	**1**	2	2	2	2
3	1	1	1	**1**	2	2	2	1
3	1	12	4	**2**	2	2	2	1
1	2	1	4	**2**	2	2	2	2
3	2	1	4	**2**	2	2	2	2
2	1	1	2	**1**	2	2	2	2
4	2	1	4	**2**	2	2	2	2
2	1	1	2	**1**	2	2	2	2
1	2	1	4	**2**	2	2	2	2
3	1	1	4	**2**	2	2	2	1
4	2	1	1	**1**	2	2	2	2
2	1	5	4	**2**	2	2	2	1
2	1	1	4	**2**	2	2	2	1
1	2	3	1	**2**	1	1	2	1
4	1	1	4	**2**	2	2	2	1
1	2	1	2	**2**	2	2	2	1
2	1	2	2	**1**	2	2	2	2
2	1	1	1	**1**	1	2	1	1
2	1	6	1	**1**	2	2	2	2
3	2	1	2	**2**	2	2	2	1
1	2	1	2	**1**	1	1	1	1
1	1	1	2	**1**	1	1	1	1
1	1	5	1	**2**	2	2	2	1
1	1	2	2	**1**	2	2	2	1
3	1	3	2	**1**	2	2	2	2
1	2	1	2	**1**	2	2	2	2
3	2	1	2	**1**	1	1	2	2
4	2	1	1	**1**	1	2	1	1
1	1	1	2	**1**	2	2	2	2
2	2	7	2	**1**	2	2	2	2
2	1	6	2	**1**	2	2	2	2
5	1	2	1	**1**	1	2	1	2
1	2	1	4	**2**	2	2	2	1
2	1	13	1	**1**	2	2	2	2
1	1	1	2	**1**	2	2	2	2
1	1	1	4	**2**	2	2	2	2
2	2	1	2	**1**	1	1	2	1
1	1	4	4	**2**	2	2	2	2
1	2	1	2	**1**	1	1	2	2
2	2	5	1	**1**	1	2	1	1
4	2	1	4	**2**	2	2	2	2

2	2	1	2	**2**	2	2	2	2
1	1	1	4	**2**	2	2	2	1
1	2	1	4	**2**	2	2	2	2
1	1	1	1	**1**	1	2	1	2
4	1	10	2	**1**	1	1	2	2
3	1	1	2	**1**	1	1	2	1
2	1	1	2	**2**	2	2	2	2
1	2	3	2	**1**	2	2	2	2
1	1	7	2	**1**	2	2	2	2
1	2	3	2	**1**	1	1	2	1
1	1	1	4	**2**	2	2	2	2
2	2	1	4	**2**	2	2	2	2
1	1	14	2	**2**	2	2	2	1
1	2	6	1	**2**	2	2	2	2
1	2	1	4	**2**	2	2	2	2
1	2	1	1	**1**	2	2	2	1
1	1	17	2	**1**	2	2	2	2
2	1	3	2	**1**	2	2	2	2
2	1	4	2	**1**	2	2	2	2
1	1	1	1	**1**	2	2	2	2
1	1	1	1	**1**	2	2	2	1
4	2	1	4	**2**	2	2	2	2
1	1	18	2	**2**	2	2	2	2
2	1	1	4	**2**	2	2	2	2
2	1	1	2	**2**	2	2	2	2
1	1	6	2	**1**	2	2	2	2
1	1	2	2	**1**	1	1	2	2
5	1	1	2	**2**	2	2	2	2
1	1	26	2	**2**	2	2	2	2
4	1	1	2	**2**	2	2	2	1
2	2	10	2	**1**	2	2	2	1
5	1	1	4	**2**	2	2	2	2
4	1	1	4	**2**	2	2	2	2
1	1	8	2	**2**	2	2	2	1
1	2	1	1	**2**	2	2	2	2
1	2	3	2	**1**	2	2	2	2
1	1	1	1	**1**	2	2	2	2
2	1	1	2	**2**	2	2	2	2
1	1	1	2	**1**	2	2	2	2
1	1	1	4	**2**	2	2	2	1
1	1	1	4	**2**	2	2	2	2
4	1	13	2	**2**	2	2	2	2
1	1	1	1	**2**	2	2	2	2
1	1	8	2	**1**	2	2	2	2
1	2	1	4	**2**	2	2	2	2
1	2	9	1	**2**	2	2	2	1
3	1	1	2	**1**	2	2	2	2

1	1	2	4	**2**	2	2	2	1
4	1	1	4	**2**	2	2	2	1
1	2	5	2	**1**	2	2	2	1
2	1	1	2	**2**	2	2	2	1
4	1	1	4	**2**	2	2	2	1
3	1	1	4	**2**	2	2	2	1
2	1	7	2	**1**	2	2	2	2
5	2	1	2	**2**	2	2	2	2
1	1	10	1	**1**	1	1	1	2
1	1	1	2	**1**	1	1	2	1
1	1	4	2	**1**	1	1	2	2
1	2	1	2	**1**	2	2	2	2
1	1	9	1	**2**	2	2	2	1
4	1	4	2	**1**	1	1	2	2
1	1	1	1	**1**	2	2	2	1
4	2	1	4	**2**	2	2	2	2
2	1	1	4	**2**	2	2	2	2
1	2	1	1	**2**	2	2	2	1
2	2	1	4	**2**	2	2	2	2
3	2	1	2	**2**	2	2	2	2
1	1	1	4	**2**	2	2	2	2
1	1	9	1	**1**	2	2	2	2
1	2	1	2	**2**	2	2	2	2
2	2	1	2	**1**	2	2	2	1
5	2	1	4	**2**	2	2	2	2
1	2	13	1	**1**	1	2	1	2
1	2	1	4	**2**	2	2	2	2
2	1	1	2	**1**	2	2	2	1
1	1	2	2	**2**	2	2	2	2
1	1	1	4	**2**	2	2	2	1
1	2	1	4	**2**	2	2	2	1
2	1	1	4	**2**	2	2	2	2
3	2	1	2	**1**	2	2	2	2
1	1	1	2	**1**	2	2	2	1
1	2	1	2	**2**	2	2	2	1
3	1	13	1	**1**	2	2	2	2
5	1	1	2	**1**	2	2	2	2
2	2	1	1	**2**	1	1	2	1
2	1	1	2	**2**	2	2	2	2
1	1	1	4	**2**	2	2	2	2
1	1	20	1	**2**	1	1	2	1
3	2	4	4	**2**	2	2	2	1
1	1	1	4	**2**	2	2	2	2
1	1	1	4	**2**	1	1	2	2
3	1	2	1	**1**	2	2	2	2
1	1	1	4	**2**	2	2	2	1
1	2	1	2	**2**	2	2	2	2

1	2	1	2	**1**	2	2	2	2
2	2	5	2	**2**	2	2	2	2
1	2	6	1	**1**	2	2	2	2
1	2	1	2	**1**	2	2	2	1
3	2	1	2	**1**	2	2	2	1
1	1	3	2	**1**	2	2	2	2
2	1	3	2	**1**	2	2	2	1
1	2	5	4	**2**	2	2	2	2
2	1	1	2	**1**	2	2	2	2
1	1	1	2	**1**	2	2	2	1
3	2	3	2	**2**	2	2	2	2
2	1	2	2	**1**	2	2	2	2
1	2	1	1	**1**	2	2	2	2
1	1	1	1	**1**	2	2	2	2
3	1	1	4	**2**	2	2	2	2
5	1	26	3	**2**	2	2	2	2
2	1	1	2	**1**	1	1	2	1
1	1	1	1	**1**	1	2	1	1
3	1	3	1	**1**	2	2	2	2
5	1	1	1	**1**	2	2	2	1
1	1	1	4	**2**	2	2	2	2
2	1	2	2	**1**	1	1	2	2
1	1	1	1	**2**	2	2	2	1
1	1	1	2	**1**	2	2	2	1
4	2	1	1	**1**	2	2	2	2
1	1	3	2	**1**	2	2	2	2
1	2	1	2	**2**	2	2	2	1
2	2	1	2	**1**	2	2	2	1
1	2	1	4	**2**	2	2	2	2
1	2	6	2	**1**	1	1	2	2
1	1	1	1	**2**	2	2	2	2
2	2	9	2	**1**	2	2	2	1
1	1	14	2	**1**	2	2	2	2
2	2	1	2	**1**	1	1	2	1
4	1	1	1	**1**	2	2	2	2
3	2	1	2	**1**	2	2	2	2
1	1	1	2	**1**	2	2	2	2
3	2	1	3	**1**	1	1	1	2
4	2	1	1	**1**	1	2	1	1
1	1	6	2	**1**	2	2	2	2
2	1	6	2	**1**	2	2	2	1
1	1	1	4	**2**	2	2	2	1
1	2	1	2	**2**	2	2	2	2
1	1	1	1	**1**	2	2	2	2
1	2	1	4	**2**	2	2	2	2
1	2	1	2	**1**	2	2	2	2
1	1	1	4	**2**	2	2	2	2

1	1	1	2	**2**	2	2	2	1
1	1	17	2	**1**	2	2	2	1
2	1	1	1	**1**	2	2	2	2
1	2	1	4	**2**	2	2	2	1
1	1	2	2	**2**	2	2	2	2
1	2	1	4	**2**	2	2	2	2
1	2	1	2	**1**	2	2	2	2
2	2	1	4	**2**	2	2	2	1
1	2	1	2	**1**	2	2	2	2
1	1	1	1	**1**	2	2	2	2
2	2	1	2	**1**	2	2	2	2
1	1	3	2	**1**	2	2	2	1
1	2	1	2	**2**	2	2	2	2
3	1	2	4	**2**	2	2	2	1
1	1	1	2	**2**	2	2	2	2
3	2	1	1	**2**	2	2	2	1
2	1	1	4	**2**	2	2	2	2
1	1	1	2	**1**	1	2	1	1
1	2	1	1	**1**	2	2	2	2
1	1	1	2	**1**	2	2	2	1
2	1	8	1	**2**	2	2	2	2
3	1	6	2	**1**	2	2	2	1
3	1	1	1	**1**	2	2	2	1
2	1	3	2	**2**	2	2	2	2
4	1	1	4	**2**	1	1	2	2
1	2	1	1	**1**	1	1	2	2
2	2	1	1	**1**	2	2	2	1
3	2	2	2	**1**	1	1	2	2
4	1	1	1	**1**	2	2	2	1
1	1	1	2	**1**	2	2	2	2
1	2	1	2	**1**	2	2	2	2
2	2	1	2	**1**	1	1	2	2
2	1		4	**2**	2	2	2	1
5	2	0	4	**2**	2	2	2	2
1	1		4	**2**	2	2	2	1
1	1	4	1	**2**	1	1	2	2
2	1	2	4	**2**	2	2	2	1
4	2	2	4	**2**	2	2	2	1
1	1		1	**2**	2	2	2	2
5	1		2	**1**	2	2	2	2
1	1	9	1	**1**	2	2	2	1
1	1	6	2	**1**	1	1	2	1
3	1		2	**1**	2	2	2	2
3	1		2	**1**	2	2	2	1
1	2		2	**1**	2	2	2	2
1	2	8	2	**2**	2	2	2	2
1	1		4	**2**	2	2	2	1

4	1	3	1	**2**	2	2	2	2
2	1	2	2	**1**	1	1	2	2
1	1	2	2	**1**	1	1	2	2
2	1	4	2	**1**	2	2	2	1
1	1		2	**1**	2	2	2	2
2	1	9	1	**1**	2	2	2	2
2	1	7	2	**1**	2	2	2	2
3	1	10	2	**1**	2	2	2	1
1	2		4	**2**	2	2	2	2
1	1	1	4	**2**	2	2	2	1
4	2		1	**1**	2	2	2	2
1	1		4	**2**	2	2	2	2
2	1	4	4	**2**	2	2	2	2
1	1		4	**2**	2	2	2	2
1	1	3	2	**1**	2	2	2	2
1	1	4	2	**1**	2	2	2	1
2	2		1	**2**	2	2	2	2
1	1	2	2	**1**	2	2	2	1
2	1		1	**1**	2	2	2	2
1	1		2	**2**	2	2	2	1
2	1	2	1	**1**	2	2	2	2
4	1		1	**2**	2	2	2	2
1	2		2	**1**	2	2	2	2
4	1	10	2	**2**	2	2	2	1
2	2		1	**1**	2	2	2	2
3	2		1	**2**	2	2	2	1
2	1		2	**1**	2	2	2	1
3	1	10	2	**1**	2	2	2	2
2	1	2	1	**1**	2	2	2	2
4	1	9	1	**1**	2	2	2	1
1	1		2	**1**	2	2	2	1
1	1		2	**1**	2	2	2	1
5	1		1	**2**	2	2	2	2
1	1	5	2	**2**	2	2	2	1
2	1	3	2	**2**	2	2	2	2
1	1	3	2	**2**	2	2	2	2
1	1		1	**2**	2	2	2	2
2	1		2	**2**	2	2	2	2
1	1		2	**1**	1	1	2	1
3	1		1	**1**	2	2	2	1
2	1		1	**2**	2	2	2	2
2	1	7	1	**2**	2	2	2	2
1	2		2	**2**	2	2	2	2
1	1		4	**2**	2	2	2	1
2	1		2	**1**	1	1	2	1
1	1	2	2	**2**	2	2	2	1
1	1		4	**2**	2	2	2	2

1	1		2	**1**	2	2	2	2
1	1	5	1	**2**	2	2	2	1
1	1		1	**1**	2	2	2	2
2	1		1	**2**	2	2	2	1
5	1	3	2	**1**	1	1	2	1
2	1	1	2	**2**	2	2	2	1
2	1	3	2	**1**	2	2	2	2
1	1	5	1	**2**	2	2	2	2
1	1	3	1	**1**	2	2	2	2
2	1		1	**1**	2	2	2	1
2	1	5	1	**1**	2	2	2	2
1	1		1	**1**	2	2	2	2
1	1	15	1	**2**	2	2	2	2
1	1	6	2	**2**	1	1	2	2
1	1		1	**1**	1	1	2	2
2	1	7	1	**1**	2	2	2	2
1	2		1	**2**	2	2	2	2
2	1		4	**2**	2	2	2	2
1	1		4	**2**	2	2	2	2
1	1		1	**2**	1	1	2	1
1	1	6	1	**1**	2	2	2	2
5	1		1	**1**	1	1	2	2
5	1	5	1	**1**	2	2	2	2
2	2		1	**1**	2	2	2	2
2			1	**1**	2	2	2	2
2	1	5	4	**2**	2	2	2	2
2	1		4	**2**	2	2	2	1
1	1		1	**1**	2	2	2	1
4	1		1	**1**	2	2	2	2
1	1	4	1	**1**	1	1	2	1
1	1		2	**1**	1	1	2	2
1	1	6	1	**1**	1	1	2	2
1	1	8	1	**2**	1	1	2	2
1	1	6	1	**1**	2	2	2	2
2	1	7	1	**1**	2	2	2	2
5	2	6	1	**1**	2	2	2	1
4	1	9	3	**1**	1	2	1	2
1	1	2	1	**2**	2	2	2	1
5	1	15	2	**2**	2	2	2	2
5	1	6	1	**1**	1	1	2	2
1	1	6	1	**1**	2	2	2	2
1	1		1	**2**	2	2	2	2
1	1	1	1	**2**	2	2	2	2
1	1	4	1	**1**	2	2	2	2
2	1		1	**2**	1	1	2	1
1	1		4	**2**	2	2	2	2
1	1		2	**1**	1	1	2	2

4	1	10	1	**1**	2	2	2	1
2	1	10	1	**1**	2	2	2	2
4	1	9	1	**2**	2	2	2	2
2			1	**1**	2	2	2	2
1	1	7	2	**2**	2	2	2	2
2	1	19	2	**1**	2	2	2	2
1	1		4	**2**	2	2	2	2
1	1	10	2	**1**	1	1	2	2
4	1	7	3	**1**	1	2	1	2
2	2		2	**1**	2	2	2	2
1	1		4	**2**	2	2	2	2
1	2		2	**1**	2	2	2	1
2	1	13	2	**2**	2	2	2	2
4			4	**2**	2	2	2	2
4	1		4	**2**	2	2	2	2
1	2		4	**2**	2	2	2	2
1	1	4	2	**1**	2	2	2	2
5	1	8	2	**2**	2	2	2	2
1	1		4	**2**	2	2	2	1
3	1		4	**2**	2	2	2	2
1	1		2	**1**	2	2	2	2
1	1	10	1	**1**	1	1	2	1
1	1		1	**1**	2	2	1	1
2	1		4	**2**	2	2	2	2
1	1		2	**1**	2	2	2	2
4	2		4	**2**	2	2	2	2
1	2		2	**1**	2	2	2	2
1	1	13	1	**1**	2	2	2	2
1	1	14	2	**1**	2	2	2	1
4								
4	2		2	**2**	2	2	2	1
2	2		2	**1**	1	1	2	1
1	1		2	**1**	2	2	2	2
2	1	8	2	**2**	2	2	2	2
1	1	7	2	**2**	2	2	2	1
2	1		4	**2**	2	2	2	2
2	2		1	**1**	2	2	2	2
2	1	15	2	**1**	2	2	2	2
2	1	50	2	**2**	2	2	2	1

GRAM +	GRAM -	Bacterial /fungal infections in sites	pre-candidemia hospital stay (days)	pre-candidemia hospital stay (days)	pre-candidemia hospital stay (days)	total pre-candidemia hospital stay (days)	7-days outcome	30-day outcome
2	2	2	0	1	0	1	1	1
1	1	1	0	37	0	37	1	1
2	2	1	21	68	0	89	1	1
2	2	1	0	22	14	36	1	1
2	2	2	0	1	0	1	1	1
2	2	2	0	6	0	6	1	1
2	1	1	4	0	4	4	1	1
1	1	1	0	30	0	30	1	1
2	2	1	0	0	80	80	1	2
2	2	1	0	0	91	91	2	2
2	2	1	0	6	20	26	1	2
2	2	2	0	0	13	13	1	1
2	2	1	0	22	0	22	1	1
2	2	1	0	3	1	4	1	2
2	2	2	3	90	37	130	1	1
2	2	1	0	2	0	2	1	1
1	2	1	80	0	0	80	1	1
2	1	1	0	0	49	49	1	1
1	2	2	0	0	17	17	1	1
2	2	2	0	17	0	17	2	2
2	2	1	90	0	1	91	2	2
1	2	2	0	3	1	4	1	1
1	2	2	0	4	6	10	1	1
1	1	2	0	10	0	10	1	1
1	2	2	0	30	0	30	1	1
2	2	1	0	5	0	5	1	1
2	2	1	0	0	36	36	1	2
2	1	1	0	12	0	12	1	1
1	2	1	0	0	30	30	1	1
2	1	1	0	0	5	5	1	1
2	2	1	0	0	1	1	1	1
2	2	2	61	0	0	61	2	2
1	2	1	0	7	0	7	1	1
2	1	2	0	0	1	1	2	2
1	2	1	0	0	34	34	1	1
2	2	1	0	0	1	1	1	2
2	2	2	6	0	7	13	1	1
2	2	1	0	0	21	21	1	1
2	1	1	0	3	12	15	1	1
2	2	1	0	0	1	1	1	1
2	2	1	0	0	37	37	1	2
2	2	2	0	1	0	1	1	1
1	2	2	0	0	1	1	2	2

2	2	1	0	1	0	1	1	1
2	1	1	0	0	29	29	1	1
2	2	2	0	33	0	33	1	1
2	2	1	0	0	28	28	1	1
1	2	1	0	3	0	3	1	1
2	2	1	40	0	17	57	1	2
2	1	2	7	0	2	9	1	1
2	2	1	62	120	30	212	1	1
1	2	1	28	0	2	30	2	2
1	2	2	60	15	0	75	1	2
2	2	1	0	1	0	1	1	1
1	1	1	10	0	29	39	1	1
2	2	2	0	18	0	18	1	1
2	2	2	0	17	0	17	1	1
2	2	2	0	13	0	13	1	1
2	2	1	29	19	0	48	1	1
2	2	1	0	6	0	6	1	1
2	2	1	0	0	45	45	1	1
2	1	1	23	3	28	54	1	1
2	2	2	0	3	0	3	1	1
2	2	2	2	0	10	12	1	1
1	2	1	0	0	10	10	2	2
2	1	1	23	3	20	46	1	1
2	2	2	28	20	0	48	1	1
2	1	1	0	86	0	86	1	1
2	2	1	0	1	0	1	1	2
2	2	2	0	0	16	16	1	1
2	2	2	0	0	30	30	1	1
2	2	2	0	28	0	28	1	2
2	1	2	10	10	0	20	1	1
2	2	2	36	0	25	61	1	1
2	2	2	7	0	18	25	1	1
2	2	2	0	13	0	13	1	1
2	2	1	0	1	0	1	1	1
2	2	2	6	0	12	18	1	1
2	2	2	0	0	1	1	2	2
1	1	2	2	0	20	22	1	1
2	2	2	0	0	0	0	1	1
2	1	1	0	0	10	10	1	1
2	2	1	12	0	14	26	2	2
2	2	1	34	0	0	34	1	1
2	2	2	0	1	0	1	1	1
2	1	2	0	0	87	87	1	1
2	1	2	0	1	0	1	1	1
2	2	1	0	46	0	46	1	1
2	2	1	0	13	0	13	1	1
2	2	1	39	0	0	39	1	1

2	2	2	98	0	0	98	1	1
2	2	1	7	0	9	16	1	1
1	2	2	0	4	20	24	1	1
2	2	1	1	0	29	30	1	2
2	2	2	2	0	13	15	1	1
1	2	1	1	12	27	40	1	1
2	2	1	16	0	6	22	1	2
2	2	1	62	0	0	62	1	1
2	2	1	0	0	31	31	1	1
2	2	2	2	4	2	8	1	1
2	2	2	71	0	16	87	1	1
2	2	2	0	1	0	1	1	1
2	2	2	0	8	0	8	1	1
2	2	1	16	0	0	16	1	1
1	2	2	5	33	0	38	1	1
2	2	2	1	0	5	6	1	1
1	2	2	5	33	0	38	1	1
1	2	2	0	10	0	10	1	1
2	2	1	0	3	0	0	1	1
2	2	1	0	43	0	43	1	2
2	2	1	0	19	15	34	1	1
1	2	2	15	14	3	22	1	1
2	2	1	0	17	2	19	1	2
2	2	2	9	0	0	9	1	1
1	2	1	0	20	0	20	1	1
2	2	2	0	1	0	1	2	2
2	2	1	0	14	0	14	1	1
2	2	2	19	29	0	48	1	1
2	2	1	0	0	2	2	1	1
2	2	1	10	0	0	10	1	1
2	2	2	0	5	0	5	1	1
2	2	1	0	33	10	43	1	2
2	2	1	0	33	10	43	1	2
2	2	1	0	37	13	50	1	1
2	2	2	0	0	43	43	2	2
2	2	2	12	27	0	39	1	2
2	2	1	0	8	0	8	1	1
2	2	1	1	0	0	1	1	1
1	2	1	0	2	0	2	1	1
2	1	2	2	0	14	16	1	2
2	2	1	0	2	0	2	1	2
2	1	1	29	0	2	31	2	2
1	2	2	32	0	1	22	1	1
2	2	1	0	0	6	6	1	1
1	2	1	11	6	19	36	1	2
2	2	1	15	0	18	33	1	1
2	2	2	6	0	10	16	1	2

1	1	1	51	0	57	108	1	1
2	2	1	0	23	0	23	1	2
2	1	1	0	3	0	3	1	1
2	1	2	4	0	12	16	1	2
1	2	2	6	0	17	23	1	2
2	2	1	0	8	0	8	1	1
2	2	1	45	0	8	53	1	1
2	1	1	0	33	0	33	1	1
1	2	2	6	14	0	15	1	1
2	2	2	0	12	0	12	1	1
2	2	1	15	0	10	25	1	1
2	2	1	0	1	10	11	1	1
2	2	1	0	0	8	8	1	1
2	2	2	0	0	35	35	1	1
2	2	2	0	8	0	8	1	1
2	2	1	30	0	0	30	1	1
2	2	1	0	0	15	15	1	1
1	2	1	7	40	60	107	1	1
2	2	1	30	0	0	30	1	1
2	1	1	36	66	0	102	1	2
2	2	1	30	0	0	30	1	1
1	2	1	0	38	0	38	1	2
2	2	2	5	0	0	5	1	1
1	2	2	6	0	17	23	1	2
2	2	1	0	1	0	1	1	1
2	1	1	0	18	0	18	2	2
2	1	1	1	0	14	15	1	1
2	1	1	1	0	14	15	1	1
1	1	2	45	0	24	69	1	1
1	2	1	8	0	26	34	1	1
2	2	2	0	21	8	29	1	2
2	2	1	10	2	0	12	1	1
2	2	1	0	12	0	12	1	1
1	1	2	7	7	0	14	1	1
2	2	2	13	0	19	32	1	1
2	2	2	3	0	0	3	1	1
2	2	2	0	30	0	30	1	2
2	2	2	0	7	0	7	1	1
1	2	2	0	101	0	101	1	1
2	2	2	0	0	38	38	1	2
2	2	2	0	0	36	36	1	2
2	2	1	0	0	18	18	1	1
1	2	1	0	0	35	35	1	1
2	2	1	2	0	48	50	1	1
2	2	1	0	8	0	8	1	2
2	1	1	0	12	31	43	1	2
2	2	2	0	27	4	31	2	2

2	2	2	0	3	0	3	1	1
1	1	2	0	0	30	30	1	2
2	2	2	0	7	0	7	1	1
2	2	2	1	0	41	42	1	1
2	2	1	0	45	0	45	1	2
1	2	2	8	0	8	16	1	1
2	2	1	41	1	13	55	1	1
2	2	1	1	0	0	1	1	1
2	2	2	30	0	0	30	1	1
2	1	1	0	0	19	19	1	2
2	2	2	19	0	16	35	1	2
2	2	2	9	0	2	11	1	1
1	2	1	2	60	15	77	1	2
2	2	1	77	0	0	77	1	1
2	2	1	15	0	0	15	1	1
1	2	2	0	11	25	36	1	1
2	2	1	0	0	1	1	2	2
2	2	1	1	0	26	27	1	2
2	2	2	20	0	40	60	1	1
2	2	2	0	1	0	1	1	1
1	2	2	0	29	16	45	2	2
2	2	1	0	22	2	24	2	2
2	2	1	0	2	0	2	1	1
2	2	2	0	16	0	16	1	1
2	2	1	2	120	0	122	1	1
2	2	1	6	0	5	11	1	2
2	2	2	14	0	0	14	1	1
2	2	2	0	0	23	23	1	1
2	2	2	0	50	0	50	2	2
2	1	1	0	0	2	2	1	1
1	2	1	4	0	60	64	1	1
2	2	1	0	14	26	38	1	2
2	2	1	13	0	23	36	2	2
1	2	2	16	0	3	19	1	1
2	2	1	23	0	0	23	1	1
2	2	1	13	0	0	13	1	1
2	2	1	32	16	10	52	1	1
2	2	2	25	9	0	36	1	2
2	2	1	32	4	10	46	1	1
2	1	2	0	17	0	17	1	1
2	2	2	3	0	2	5	1	1
2	2	2	2	0	44	46	1	1
2	2	1	12	0	0	12	1	1
2	2	1	0	0	31	31	1	1
2	2	1	0	0	1	1	2	2
2	1	1	0	27	0	27	1	1
2	2	1	0	0	14	14	1	1

2	1	2	0	0	18	18	1	1
2	1	2	0	17	0	17	1	1
1	1	1	45	0	19	64	1	1
2	1	2	0	1	0	1	1	1
2	1	1	0	0	7	7	2	2
1	1	1	5	0	25	30	1	2
2	2	2	13	16	0	29	1	1
2	2	2	0	0	0	0	1	1
2	2	2	0	18	0	18	1	2
2	1	1	41	0	10	51	1	1
2	2	1	23	0	0	23	1	1
2	2	1	0	1	0	1	1	1
2	1	1	0	20	0	20	1	1
2	2	1	23	0	0	23	1	1
2	1	1	0	0	8	8	1	2
2	2	2	0	2	0	2	1	1
2	2	1	4	23	36	63	1	1
1	2	1	0	22	0	22	1	2
2	2	1	0	1	0	1	1	1
2	2	2	0	1	0	1	1	1
2	2	2	0	13	0	13	1	1
2	2	2	0	27	0	27	2	2
2	2	2	0	27	28	55	1	1
2	1	2	7	0	9	16	1	1
2	2	1	0	0	16	16	1	1
2	2	1	0	20	0	20	2	2
2	2	1	0	0	17	17	1	1
1	2	2	0	1	0	1	1	1
2	2	1	2	0	9	11	1	1
2	1	1	11	0	0	11	2	2
1	1	1	0	0	46	46	1	1
2	2	2	1	0	1	2	1	1
2	2	2	0	4	0	4	1	1
2	1	2	0	74	0	74	1	2
1	1	1	0	26	0	26	2	2
2	2	1	0	18	0	18	1	2
2	2	1	0	1	0	1	1	1
2	1	1	0	7	99	106	1	1
2	2	2	0	1	0	1	1	1
2	2	2	4	0	6	10	1	2
1	2	2	83	0	0	83	1	1
1	2	2	0	0	4	4	1	1
2	2	2	0	7	0	7	1	1
2	2	2	0	39	0	39	1	1
2	2	1	89	0	0	89	1	1
2	1	2	0	60	0	60	1	1
2	2	1	0	0	1	1	1	2

2	2	1	0	20	21	41	1	2
2	2	2	0	3	0	3	1	1
2	2	2	79	0	0	79	1	2
1	1	2	0	7	0	7	1	2
1	1	2	20	0	0	20	1	1
2	2	1	50	0	24	74	1	2
2	1	1	0	2	0	2	1	1
2	2	2	0	10	22	32	1	1
2	2	1	0	7	0	7	1	1
1	2	1	1	0	2	3	2	2
2	2	1	0	1	0	1	1	1
2	2	1	46	0	23	69	1	1
2	2	2	0	0	2	2	1	2
2	2	2	14	0	1	15	1	1
2	2	1	0	0	1	1	2	2
2	2	2	23	0	0	23	1	1
1	2	1	0	8	0	8	1	1
1	1	2	0	2	0	2	1	2
2	2	1	22	0	0	22	1	1
2	1	2	14	0	0	14	1	1
2	2	2	0	63	0	63	2	2
2	2	2	20	0	3	23	1	1
1	2	1	0	113	0	113	2	2
1	2	2	0	1	0	1	2	2
2	2	1	0	5	0	5	1	2
2	2	1	0	20	0	20	1	2
1	2	1	2	0	37	39	1	1
1	2	1	2	0	48	50	1	1
2	2	2	20	0	20	40	1	1
2	2	1	0	6	0	6	2	2
2	2	2	0	0	5	5	1	2
1	1	2	0	21	0	21	1	1
2	2	1	0	12	0	12	1	1
1	2	1	3	3	135	141	1	1
2	2	1	0	43	1	44	2	2
2	2	1	8	0	0	8	1	2
2	2	2	77	7	0	84	2	2
2	2	1	0	9	0	9	1	1
2	1	2	7	0	5	12	1	2
2	2	1	3	0	18	21	1	1
1	2	1	16	0	6	22	1	1
2	1	2	0	0	45	45	2	2
2	2	1	0	11	0	11	1	1
2	2	1	0	20	0	20	2	2
2	2	1	10	0	1	17	2	2
2	2	1	0	26	0	26	1	1
2	2	2	12	0	0	12	1	1

2	1	1	0	16	0	16	1	1
1	2	2	0	1	0	1	1	1
2	2	1	10	0	0	10	1	1
1	1	1	0	33	0	33	1	1
2	2	2	25	0	0	25	1	1
2	2	2	0	11	0	11	2	2
2	2	1	20	0	1	21	2	2
1	2	1	0	1	0	1	1	1
2	2	1	0	0	1	1	1	1
2	2	2	0	0	25	25	2	2
2	2	1	0	0	3	3	1	1
2	1	2	0	0	2	2	1	2
2	2	2	0	9	0	9	1	2
1	2	2	2	0	93	95	1	1
2	2	1	0	1	0	1	1	1
2	1	2	0	13	0	13	1	2
2	2	2	0	1	0	1	1	2
1	2	2	0	36	0	36	1	1
2	2	2	15	20	3	38	1	2
1	2	1	13	31	0	44	1	1
2	2	1	26	0	8	34	1	2
1	1	1	0	17	0	17	1	2
2	1	1	0	1	0	1	1	2
2	2	1	47	0	0	47	1	1
2	2	2	0	0	7	7	1	1
2	2	1	0	19	0	19	1	1
1	2	2	0	18	0	18	1	1
2	2	1	31	0	0	31	1	1
1	2	2	40	0	0	40	1	1
2	2	2	0	21	0	21	2	2
2	2	1	14	0	0	14	1	1
2	2	1	0	0	34	34	1	2
1	2	1	34	10	0	44	1	2
2	2	2	0	0	0	0	1	1
1	2	1	0	18	11	29	1	1
2	2	1	0	0	58	58	2	1
1	1	1	5	2	27	34	2	2
1	1	1	5	2	27	34	2	2
2	2	2	1	0	13	14	2	1
2	2	1	0	27	0	27	1	1
1	2	1	0	7	0	7	1	1
1	2	2	0	16	0	16	1	1
2	2	2	0	2	0	2	1	1
2	1	1	0	25	24	49	1	1
2	2	1	3	0	3	6	1	1
2	2	1	1	0	0	1	1	1
2	1	1	0	10	0	10	1	1

2	2	1	0	27	0	27	1	1
2	2	1	0	20	0	20	1	1
2	2	1	0	20	0	20	1	1
1	1	1	0	12	5	17	1	1
2	2	2	0	14	0	14	1	1
2	2	1	0	24	0	24	1	1
2	2	1	0	0	6	6	1	1
2	1	1	0	0	14	14	1	1
2	2	1	0	0	0	0	1	1
1	2	2	6	23	5	34	2	2
2	2	1	0	0	1	1	1	1
2	2	2	7	0	20	27	1	1
2	2	1	0	0	3	3	2	2
2	2	2	0	0	3	3	2	2
2	2	1	2	0	12	14	1	1
1	2	1	10	0	4	14	1	1
2	2	1	0	0	0	0	1	1
1	2	2	19	35	8	52	1	1
2	2	1	0	0	1	1	1	2
1	2	1	0	0	4	4	1	2
2	2	1	15	0	3	18	2	2
2	2	1	0	0	11	11	1	1
2	2	1	0	23	3	26	1	1
1	1	1	0	1	0	1	1	1
2	2	1	0	35	0	35	1	1
1	2	1	0	0	0	0	1	1
1	2	2	0	0	0	0	1	1
2	2	2	0	2	0	2	1	1
2	2	2	0	23	0	23	2	2
1	2	1	0	15	0	15	1	2
1	2	1	0	7	0	7	1	1
1	2	2	0	20	0	20	1	1
2	2	2	6	0	6	12	1	1
1	2	2	0	1	5	6	1	1
2	2	2	0	5	0	5	1	1
2	2	2	0	5	0	5	1	1
2	2	2	24	0	0	24	1	1
2	2	1	0	0	19	19	1	1
1	1	2	14	35	25	74	1	1
1	2	1	49	0	0	49	1	1
2	2	1	0	25	4	29	1	1
2	2	2	0	1	22	23	1	2
2	2	1	7	17	13	37	1	1
1	1	2	16	0	3	19	1	1
1	2	1	2	0	46	48	2	2
1	2	1	3	0	12	15	1	1
2	2	2	3	0	40	43	1	1

2	2	2	3	0	0	3	1	1
2	1	2	0	1	0	1	1	1
2	2	1	0	0	8	8	1	1
1	2	1	30	0	0	30	1	1
2	2	1	11	0	0	11	1	1
1	2	2	14	0	0	14	1	1
2	2	2	1	0	0	1	1	1
2	2	1	0	17	0	17	1	2
2	2	2	0	30	0	30	1	1
1	1	2	0	1	0	1	2	2
2	2	1	0	0	1	1	1	1
2	2	2	0	0	1		1	1
2	2	1	0	0	40	40	1	1
2	2	1	0	0	60	60	1	1
2	2	1	0	0	75	75	2	2
2	2	2	27	0	0	27	1	1
2	2	1	0	0	14	14	1	2
2	2	2	1	0	0	1	1	1
2	2	2	1	0	0	1	1	1
1	2	1	32	0	0	32	1	1
2	2	2	0	37	37	0	1	1
2	2	1	0	75	0	75	1	2
2	2	2	0	20	0	20	1	1
2	2	1	0	1	0	1	1	1
2	2	1	2	0	0	2	1	1
2	2	2	25	0	0	25	1	1
1	2	1	100	20	120	120	2	2
1	2	2	0	21	0	21	1	1
2	2	1	0	33	0	33	1	1
1	2	2	9	28	0	37	1	1
2	2	1	0	6	7	13	2	2
2	2	1	14	12	0	2	1	1
2	2	2	0	11	0	11	1	1
2	2	1	23	10	0	33	1	1
2	2	1	0	24	15	39	1	1
1	1	1	0	26	34	60	1	1
2	2	2	0	28	0	28	1	1
1	2	2	0	12	0	12	1	1
2	2	1	0	14	0	14	1	1
2	2	1	0	19	0	19	1	1
2	2	2	0	5	7	12	1	1
2	2	2	0	11	0	11	1	2
2	2	1	0	36	0	36	1	1
2	2	2	0	20	0	20	1	1
2	1	2	0	15	0	15	2	2
2	2	2	0	29	0	29	2	2
2	2	2	11	0	0	11	2	2

1	2	2	0	1	0	1	1	1
2	2	1	0	13	0	13	1	1
2	2	1	0	7	0	7	1	1
2	2	1	0	1	0	1	1	1
2	2	1	0	33	0	33	1	1
2	2	1	0	40	0	40	1	1
2	2	2	0	36	0	36	2	2
2	2	1	0	13	60	73	1	1
2	2	2	0	1	0	1	1	1
2	2	1	0	26	0	26	1	1
2	2	2	0	21	0	21	2	2
1	2	1	0	8	0	8	2	2
2	2	1	0	1	0	1	1	1
2	2	2	0	2	0	2	2	2
2	2	2	10	13	0	23	2	2
2	2	1	0	8	0	8	2	2
2	2	2	0	5	0	5	1	1
2	2	2	0	5	0	5	1	1
1	2	2	0	22	0	22	1	2
2	2	2	0	20	0	20	2	2
2	2	2	0	15	0	15	1	1
1	1	1	0	1	0	1	1	1
1	1	1	0	25	0	25	2	2
2	2	2	0	0	18	18	2	2
2	2	1	0	0	20	20	1	1
2	2	2	0	0	3	3	2	2
2	2	2	0	0	1	1	2	2
2	2	1	0	40	1	41	1	1
1	1	2	0	0	1	1	1	1
							1	1
2	1	2	0	0	21	21	1	1
1	1	1	0	80	0	80	1	1
2	2	1	0	30	0	30	2	2
2	2	1	0	17	0	17	1	2
1	2	2	0	19	0	19	1	1
2	2	2	0	7	0	7	2	2
2	2	1	38	32	0	70	1	1
2	2	2	0	33	0	33	1	1
1	2	1	0	25	1	26	1	1

Charlson score
2
0
1
2
1
1
2
1
1
1
1
1
2
1
2
2
2
1
3
4
2
2
2
2
2
2
3
2
3
3
3
2
1
3
0
3
2
3
2
3
3
3
3

5
3
3
2
3
3
3
3
4
3
3
3
3
3
2
2
4
4
4
4
3
3
4
4
4
4
4
0
10
4
4
3
4
4
3
4
3
3
4
5
2
3
5
4
4
4
4

2
4
0
4
6
6
4
2
3
2
6
4
4
1
4
4
4
4
4
4
4
4
6
4
8
4
0
4
4
4
4
0
0
4
6
4
5
2
4
4
4
4
4
4
4
0
7

4
4
4
4
7
5
5
4
2
2
2
3
5
3
4
5
5
6
5
4
5
5
3
7
2
5
2
2
5
3
1
6
6
4
0
4
0
9
4
7
6
4
4
4
6
4
6

4
6
7
7
5
5
5
4
6
5
7
6
6
5
6
6
6
6
5
6
6
5
4
6
5
4
6
5
5
6
5
7
7
5
3
5
5
5
5
5
5
5
7
7
7
5
7

4
5
5
7
2
7
4
3
7
5
5
5
4
5
7
5
8
7
5
6
2
8
8
9
6
5
9
5
8
9
8
5
5
5
4
7
5
9
5
8
5
5
5
5
6
9
6

9
6
6
6
3
6
6
9
6
3
6
6
9
6
6
6
6
6
6
6
11
6
10
6
6
6
6
6
6
7
7
11
7
7
13
7
7
7
7
7
7
7
1
7
7
7
7

7
7
7
7
7
8
8
8
5
8
0
8
8
0
8
8
8
8
9
9
9
9
1
9
9
9
9
0
10
10
10
11
9
5
6
5
3
3
8
12
6
8
6
7
9
2
3

7
6
6
5
9
9
6
5
6
10
1
11
6
7
2
2
9
6
3
10
4
4
5
6
1
8
4
7
4
8
9
7
0
5
4
4
11
5
4
3
6
5
7
5
4
5
9

3
6
7
9
1
3
6
8
4
8
10
6
6
7
5
4
5
8
1
7
7
12
5
6
5
6
8
6
9
7
13
4
9
6
1
5
1
1
5
5
4
7
5
4
2
4
9

12
7
7
7
3
9
7
9
1
3
10
11
5
7
6
8
6
6
9
4
10
6
6
7
10
10
8
8
8
6
8
9
7
8
7
7
5
7
