# Supplementary material for: Candidemia in intensive care units over nine years at a large Italian university hospital: Comparison with other wards
Source: PLoS One. 2021 May 26;16(5):e0252165. doi: 10.1371/journal.pone.0252165 (PMC8153423; doi:10.1371/journal.pone.0252165)
Supplement: S2 File — (PDF) [file pone.0252165.s002.pdf]

n.	Ward at the time of candidemia	Year	Candida spp.	MIC of Amphotericin B
1	3	2015	Glabrata	S0,5
2	1	2016	Albicans	S 1
3	1	2014	Tropicalis	S 0.5
4	2	2014	Parapsilosis	S 0.25
5	1	2010	Albicans	S 0.5
6	2	2013	Albicans	S<= 0.12
7	2	2013	albicans	S<= 0.12
8	2	2018	Parapsilosis	1
9	2	2014	albicans	S 0.5
10	3	2016	Parapsilosis	S0,5
11	1	2014	Albicans	S 0.5
12	3	2018	Albicans	0,5
13	2	2018	Parapsilosis	0,5
14	2	2014	Tropicalis	S 0.5
15	2	2018	Parapsilosis	0,5
16	1	2013	Guillermondii	S<= 0.12
17	1	2016	Tropicalis	S 0,5
18	2	2010	Albicans	S 0.25
19	1	2018	Parapsilosis	1
20	1	2017	Parapsilosis	S0,5
21	2	2016	Albicans	S0,25
22	1	2010	Glabrata	S 1
23	2	2015	Albicans	S1
24	2	2017	Rugosa	/
25	3	2010	Albicans	S 0.25
26	2	2013	albicans	S <0.12
27	2	2012	Parapsilosis	S 0.25
28	2	2013	Parapsilosis	S 0.5
29	1	2018	Albicans	1
30	2	2018	Parapsilosis	0,5
31	2	2015	Albicans	S0,5
32	2	2017	Albicans	S0,5
33	3	2018	Albicans	1
34	3	2017	Guillermondii	/0,25
35	1	2016	Parapsilosis	S1
36	2	2015	Parapsilosis	S0,25
37	2	2012	Albicans	S 0.5
38	2	2012	Albicans	S 0.5
39	1	2018	Glabrata	2
40	1	2010	albicans	S 0.12
41	1	2018	Albicans	1
42	2	2013	Parapsilosis	S 0.5
43	1	2014	Albicans	S 0.5
44	1	2010	albicans	S 1

45	2	2018	Albicans	0,5
46	2	2010	albicans	S 0.25
47	2	2010	Glabrata	S 1
48	2	2012	Albicans	S 0.25
49	2	2013	albicans	S 0.5
50	2	2014	albicans	S 0.5
51	1	2011	Albicans	S 0.5
52	2	2015	albicans	S0,5
53	3	2011	Parapsilosis	S 0.5
54	1	2014	albicans	S 0.25
55	2	2011	Parapsilosis	S 1
56	1	2010	albicans	S 0.5
57	2	2010	albicans	S 0.5
58	3	2013	albicans	S 0.5
59	2	2013	albicans	S 0.25
60	2	2010	albicans	S 0.25
61	2	2018	Albicans	1
62	1	2013	Glabrata	S 1
63	1	2014	Parapsilosis	S 0.5
64	1	2015	Parapsilosis	S1
65	1	2016	Tropicalis	S1
66	2	2015	Glabrata	S1
67	2	2014	pelliculosa	S 0.25
68	2	2012	Parapsilosis	S 0.5
69	3	2013	Parapsilosis	S 0.5
70	1	2015	Albicans	S1
71	2	2017	Albicans	S1
72	3	2014	albicans	S 0.5
73	1	2014	Parapsilosis	S 0.5
74	1	2015	albicans	S1
75	2	2018	Parapsilosis	1
76	1	2010	Parapsilosis	S 0.25
77	1	2011	Parapsilosis	S 0.5
78	2	2010	Parapsilosis	S 0.5
79	2	2010	Parapsilosis	S0.5
80	2	2015	Tropicalis	S 1
81	2	2018	Parapsilosis	0,5
82	3	2015	Albicans	S0,5
83	2	2017	Albicans	S0,5
84	1	2017	Albicans	S0,5
85	1	2015	albicans	S 0,5
86	3	2010	albicans	S 0.12
87	3	2011	albicans	S 1
88	2	2011	Albicans	S 0.5
89	1	2018	Albicans	0,5
90	2	2013	Parapsilosis	S 0.75
91	2	2014	Parapsilosis	S 0.5

92	2	2013	Glabrata	S 0.5
93	3	2011	Parapsilosis	S 0.5
94	1	2015	Parapsilosis	S 0,5
95	3	2016	Albicans	S0,5
96	1	2017	Parapsilosis	S0,25
97	3	2016	Tropicalis	S1
98	1	2014	Glabrata	S 0.5
99	1	2017	Albicans	S0,5
100	3	2017	Albicans	S0,25
101	3	2010	albicans	S 0.5
102	2	2015	Albicans	S1
103	2	2018	Albicans	0,25
104	1	2010	albicans	S 0.5
105	2	2014	Albicans	S 0.5
106	2	2014	Glabrata	S 0.25
107	1	2011	Parapsilosis	S 0.5
108	3	2013	Albicans	S 0.25
109	2	2013	Tropicalis	S 1
110	3	2018	Albicans	0,5
111	1	2011	Guillermondii	S 1
112	2	2018	Albicans	0,5
113	2	2013	Parapsilosis	S<=0.12
114	2	2011	Albicans	S 0.5
115	3	2017	Albicans	S0,5
116	2	2012	Albicans	S 0.5
117	1	2014	Glabrata	S 0.5
118	3	2014	Parapsilosis	S 0.5
119	1	2013	Glabrata	S 0.25
120	3	2012	Albicans	S 0.5
121	1	2013	Albicans	S < 0.12
122	2	2018	Albicans	0,5
123	3	2012	Albicans	S 0.5
124	3	2018	Parapsilosis	0,5
125	1	2016	Albicans	S 0,5
126	3	2011	Parapsilosis	S 0.25
127	1	2017	Parapsilosis	S 0,5
128	1	2011	Tropicalis	S 1
129	1	2010	albicans	S 0.25
130	1	2017	Tropicalis	S0,5
131	2	2013	albicans	S 0.5
132	1	2010	krusei	S 1
133	1	2018	Glabrata	1
134	2	2012	Parapsilosis	S 0.5
135	3	2011	albicans	S 0.5
136	2	2018	Albicans	1
137	3	2013	krusei	S 1

138	2	2018	Krusei	0,25
139	2	2017	Tropicalis	S0,5
140	1	2016	Parapsilosis	S 0,5
141	3	2014	Parapsilosis	S 0.25
142	3	2012	Albicans	S 0.5
143	3	2017	Albicans	S0,5
144	2	2011	Parapsilosis	S 0.5
145	1	2010	albicans	S 0.5
146	2	2018	Parapsilosis	1
147	1	2011	albicans	S 0.5
148	1	2017	Parapsilosis	S0,5
149	2	2017	Parapsilosis	S0,5
150	2	2017	Albicans	S0,5
151	2	2018	Parapsilosis	0,5
152	2	2018	Parapsilosis	1
153	2	2018	Albicans	0,5
154	3	2018	Guillermondii	0,5
155	3	2018	Albicans	0,25
156	2	2017	Albicans	S1
157	2	2017	Guillermondii	/ 0,25
158	2	2018	Glabrata	
159	1	2015	albicans	S0,5
160	1	2014	Albicans	S 0.25
161	1	2012	Albicans	S 0.5
162	2	2014	Albicans	S 0.25
163	2	2013	Tropicalis	S 0.5
164	3	2012	Glabrata	S 0.5
165	1	2016	Albicans	S0,5
166	2	2018	Glabrata	0,5
167	1	2017	Albicans	S0,5
168	1	2011	Tropicalis	S 0.5
169	2	2014	albicans	S 0.25
170	2	2018	Lusitanie	0,5
171	2	2018	Guillermondii	0,5
172	2	2016	Albicans	S0,5
173	1	2010	albicans	S 0.5
174	2	2017	Parapsilosis	S0,5
175	3	2014	Tropicalis	S 1
176	1	2014	Tropicalis	S 0.5
177	3	2010	albicans	S 0.25
178	1	2013	Albicans	S<= 0.12
179	2	2014	albicans	S 1
180	2	2013	Glabrata	S 0.5
181	1	2017	Albicans	S0,5
182	1	2017	Albicans	S1
183	2	2011	albicans	S 1
184	2	2010	Tropicalis	S 1

185	3	2010	albicans	S 0.5
186	2	2017	Glabrata	S0,5
187	2	2018	Albicans	0,5
188	1	2013	albicans	S<= 0.12
189	3	2015	Albicans	S0,5
190	2	2015	Albicans	S 0,5
191	3	2014	albicans	S 0.5
192	2	2014	Tropicalis	S 0.25
193	2	2018	Albicans	1
194	1	2012	Tropicalis	S 1
195	2	2016	Parapsilosis	S 0,5
196	3	2011	albicans	S 0.5
197	1	2014	Parapsilosis	S 0.5
198	2	2014	Parapsilosis	S 0.5
199	1	2018	Albicans	1
200	2	2016	Albicans	S0,25
201	2	2016	Glabrata	S1
202	3	2016	Parapsilosis	S1
203	3	2016	Tropicalis	S1
204	2	2018	Albicans	0,5
205	1	2010	Glabrata	S 1
206	1	2017	Albicans	S0,5
207	2	2018	Albicans	0,5
208	2	2015	albicans	S0,5
209	3	2014	Parapsilosis	S 0.5
210	2	2013	albicans	S<= 0.12
211	2	2018	Albicans	0,5
212	1	2013	Albicans	S 0.5
213	2	2016	dubliniensis	S0,5
214	1	2012	albicans	S 0.5
215	3	2018	Parapsilosis	0,5
216	3	2015	Albicans	S0,5
217	2	2017	Albicans	S0,5
218	2	2017	Parapsilosis	S0,5
219	3	2013	albicans	S 0.5
220	1	2017	Glabrata	S1
221	1	2017	Parapsilosis	S0,5
222	2	2018	Parapsilosis	0,5
223	2	2016	Tropicalis	S1
224	2	2014	albicans	S 0.25
225	1	2010	Glabrata	S 1
226	3	2013	albicans	S 0.5
227	3	2012	albicans	S 0.5
228	2	2015	Tropicalis	S1
229	2	2015	Parapsilosis	S 0,5
230	2	2017	Glabrata	S0,5

231	2	2012	Guillermondii	S 0.5
232	1	2012	Albicans	S 0.5
233	1	2016	Albicans	S0,5
234	1	2017	Albicans	S0,5
235	2	2016	Albicans	S0,5
236	3	2018	Albicans	0,5
237	3	2012	Albicans	S 0.5
238	1	2017	Albicans	S0,5
239	1	2018	Parapsilosis	0,5
240	2	2016	Albicans	S1
241	1	2012	Tropicalis	S 0.5
242	1	2015	Glabrata	S0,5
243	1	2017	Parapsilosis	S0,5
244	3	2017	Tropicalis	S1
245	1	2017	Glabrata	S0,5
246	3	2014	albicans	S 0.5
247	2	2016	Parapsilosis	S1
248	1	2011	albicans	S 0.5
249	2	2013	albicans	S 0.25
250	1	2015	albicans	S0,5
251	2	2015	Albicans	S 0.5
252	2	2016	dubliniensis	S0,25
253	2	2013	Parapsilosis	S 0.25
254	2	2014	Parapsilosis	S 0.25
255	2	2017	Albicans	S0,5
256	2	2018	Albicans	0,5
257	2	2015	Parapsilosis	S0,5
258	3	2015	Parapsilosis	S0,5
259	1	2015	albicans	S1
260	1	2018	Glabrata	1
261	2	2012	Glabrata	S 1
262	2	2017	Glabrata	S1
263	3	2015	Albicans	S0,5
264	1	2016	Albicans	S0,5
265	2	2014	Tropicalis	S 0.5
266	2	2018	Albicans	0,5
267	2	2016	Tropicalis	S1
268	3	2017	Albicans	S0,25
269	2	2016	Parapsilosis	S0,5
270	2	2015	Albicans	S 0,5
271	2	2016	Albicans	S0,5
272	3	2014	Parapsilosis	S 0.5
273	3	2014	Albicans	S 0.5
274	2	2015	Albicans	S0,25
275	2	2018	Albicans	1
276	2	2018	Guillermondii	0,5

277	2	2011	Parapsilosis	S 0.5
278	1	2014	Parapsilosis	S 0.5
279	1	2014	Parapsilosis	S 0.25
280	1	2017	Glabrata	S1
281	3	2013	Glabrata	S 0.5
282	1	2011	Parapsilosis	S 0.5
283	1	2013	albicans	S 0.5
284	2	2017	Parapsilosis	S0,25
285	2	2011	albicans	S 0.5
286	3	2013	albicans	S 0.25
287	3	2010	albicans	S 0.25
288	2	2018	Albicans	0,5
289	2	2015	Albicans	S 0,5
290	2	2018	Tropicalis	1
291	2	2011	albicans	S 0.25
292	2	2016	Tropicalis	S1
293	2	2016	Albicans	S0,06
294	2	2015	Tropicalis	S0.5
295	2	2018	Albicans	1
296	3	2013	albicans	S<=0.12
297	3	2013	Glabrata	S<=0.12
298	2	2015	Parapsilosis	S 0.5
299	2	2016	Albicans	S0,5
300	2	2018	Albicans	0,5
301	2	2018	Albicans	1
302	1	2012	albicans	S 0.5
303	3	2017	Albicans	S0,5
304	2	2016	Parapsilosis	S0,5
305	1	2013	Albicans	S 0.5
306	3	2014	Albicans	S<= 0.12
307	2	2018	Albicans	0,5
308	3	2018	Parapsilosis	1
309	1	2011	Glabrata	S 0.5
310	1	2010	Albicans	S 0.25
311	2	2016	Parapsilosis	S 1
312	2	2018	Albicans	0,5
313	3	2016	Parapsilosis	S0,25
314	2	2011	albicans	S 0.5
315	3	2012	Parapsilosis	S 0.25
316	2	2015	albicans	S0,5
317	1	2011	albicans	S 1
318	2	2013	albicans	S<= 0.12
319	2	2016	Parapsilosis	S1
320	2	2015	Glabrata	S 1
321	2	2018	Parapsilosis	0,5
322	2	2018	Albicans	1
323	1	2015	Parapsilosis	S0,5

324	2	2013	Parapsilosis	S 0.5
325	2	2013	Albicans	S 0.25
326	2	2011	albicans	S 0.5
327	1	2011	Tropicalis	S 0.5
328	3	2016	Parapsilosis	S0,5
329	1	2016	Glabrata	S1
330	2	2014	Parapsilosis	S 0.5
331	1	2012	Parapsilosis	S 0.5
332	1	2011	albicans	S 0.5
333	3	2012	albicans	S 0.25
334	1	2011	Parapsilosis	S 0.5
335	3	2015	Albicans	S0,5
336	3	2015	Glabrata	S1
337	2	2015	albicans	S0,25
338	1	2010	Albicans	S<= 0.12
339	3	2014	Parapsilosis	S 0.25
340	3	2012	Tropicalis	S 0.5
341	1	2017	Albicans	S0,25
342	1	2011	albicans	S 0.5
343	1	2012	Parapsilosis	S 0.5
344	1	2013	albicans	S 0.5
345	2	2017	tropicalis	S1
346	1	2012	Parapsilosis	S 0.25
347	1	2011	albicans	S 0.5
348	1	2016	Guillermondii	S
349	2	2018	Albicans	1
350	2	2018	Albicans	1
351	2	2010	Tropicalis	S 0.12
352	3	2015	Glabrata	S1
353	2	2017	Albicans	S0,5
354	2	2013	albicans	S 0.5
355	3	2017	albicans	S0,25
356	2	2018	Parapsilosis	0,5
357	2	2015	albicans	S0,25
358	2	2011	albicans	S 0.5
359	3	2014	Glabrata	S 0.5
360	3	2017	albicans	S0,25
361	1	2013	Glabrata	S<= 0.12
362	1	2013	Glabrata	S 0.125
363	3	2011	albicans	S 0.5
364	3	2013	Tropicalis	S 0.5
365	2	2014	albicans	S 0.25
366	1	2017	Parapsilosis	S0,5
367	1	2013	Glabrata	S 0.5
368	3	2012	Parapsilosis	S 0.5
369	3	2013	Parapsilosis	S 0.5
370	3	2013	Tropicalis	S 0.5

371	2	2010	Tropicalis	S 1
372	2	2017	Parapsilosis	S0,5
373	3	2018	Parapsilosis	1
374	2	2014	albicans	S 0.5
375	1	2016	Albicans	S0,5
376	2	2015	albicans	S 0,5
377	2	2018	Albicans	1
378	1	2017	Albicans	S0,5
379	2	2015	albicans	S0,25
380	3	2015	Tropicalis	S1
381	1	2017	Parapsilosis	S1
382	1	2012	albicans	S 0.5
383	1	2015	Parapsilosis	S1
384	1	2015	albicans	S0,25
385	2	2010	Parapsilosis	S 0.25
386	3	2018	Parapsilosis	1
387	3	2013	Albicans	S 0.5
388	1	2013	albicans	S 0.5
389	1	2012	albicans	S 0.5
390	1	2012	albicans	S1
391	3	2014	albicans	S 0.5
392	3	2013	Parapsilosis	S 0.25
393	2	2014	Albicans	S 0.5
394	2	2015	albicans	S 0,5
395	2	2017	Albicans	S0,5
396	2	2017	Parapsilosis	S0,5
397	3	2013	Albicans	S<= 0.12
398	1	2010	Parapsilosis	S 0.5
399	3	2016	Albicans	S0,5
400	3	2018	Albicans	1
401	1	2013	Glabrata	S 0.25
402	2	2012	albicans	S 0.5
403	3	2014	Parapsilosis	S 0.5
404	3	2014	Parapsilosis	S 0.5
405	2	2013	Parapsilosis	S<=0.12
406	2	2016	Albicans	S0,5
407	1	2015	albicans	S0,25
408	1	2011	albicans	S 1
409	2	2015	Parapsilosis	S0,5
410	2	2012	lusitanie	S 1
411	2	2013	albicans	S <0.12
412	2	2013	albicans	S <0.12
413	2	2018	Lusitanie	0,5
414	2	2018	Glabrata	0,5
415	2	2018	Parapsilosis	1
416	2	2018	Parapsilosis	1
417	2	2018	Parapsilosis	1

418	1	2013	Glabrata	S 0.5
419	3	2010	Albicans	S 0.5
420	2	2018	Glabrata	1
421	2	2016	Parapsilosis	S1
422	3	2010	Albicans	S 0.5
423	1	2017	Parapsilosis	S0,25
424	2	2018	Albicans	1
425	2	2015	lusitanie	S<=0,12
426	2	2010	Albicans	S 0.5
427	1	2011	Guillermondii	S 0.25
428	2	2015	Albicans	S0,5
429	2	2015	Parapsilosis	S1
430	1	2016	Albicans	S0,5
431	1	2011	Albicans	S 0.5
432	2	2017	Parapsilosis	S0,5
433	2	2016	Parapsilosis	S0,25
434	3	2011	Tropicalis	S 1
435	2	2010	Albicans	S 0.5
436	2	2011	Parapsilosis	S 0.5
437	2	2013	albicans	S 0.12
438	1	2012	albicans	S 0.5
439	2	2018	Albicans	0,5
440	1	2014	albicans	S 0.5
441	1	2018	Parapsilosis	0,5
442	2	2018	Albicans	1
443	2	2017	Albicans	S0,5
444	1	2011	Parapsilosis	S 0.25
445	2	2017	Parapsilosis	S0,5
446	1	2017	Albicans	S0,5
447	1	2013	Parapsilosis	S 0.5
448	2	2012	Parapsilosis	S 0.5
449	1	2010	Tropicalis	S 1
450	1	2015	Parapsilosis	S0,25
451	1	2018	Glabrata	1
452	2	2018	Parapsilosis	1
453	3	2018	Parapsilosis	0,5
454	2	2015	Glabrata	S1
455	2	2018	Albicans	1
456	2	2018	Albicans	1
457	2	2010	Albicans	S 0.25
458	2	2016	Albicans	S0,5
459	2	2016	Parapsilosis	S1
460	2	2015	albicans	S1
461	1	2010	Albicans	S 1
462	2	2016	Albicans	S0,25
463	2	2013	Tropicalis	S 0.5
464	1	2014	albicans	S 0.5

465	1	2014	albicans	S 0.5
466	2	2016	Albicans	S0,5
467	2	2014	albicans	S 0.5
468	2	2018	Albicans	0,5
469	1	2017	Tropicalis	S1
470	2	2017	Tropicalis	S1
471	2	2017	Albicans	S0,5
472	3	2013	Tropicalis	S 0.5
473	2	2018	Albicans	0,5
474	2	2018	Glabrata	1
475	2	2018	Glabrata	1
476	1	2014	albicans	S 0.5
477	1	2015	albicans	S0,5
478	1	2017	Parapsilosis	S0,25
479	2	2017	Albicans	S0,5
480	2	2018	Kefyr	1
481	2	2010	Tropicalis	S 0.25

Susceptibility according to EUCAST (Amphotericin B)	Susceptibility according to CLSI (Amphotericin B)	MIC of Anidulafungin
S	S	S0,015
S	S	S0,015
S	S	S 0.12
S	S	S 2
S	S	S <0.015
S	S	S<= 0.015
S	S	S<= 0.015
S	S	4
S	S	S 0.06
S	S	I1
S	S	S 0.06
S	S	0,015
S	S	1
S	S	S 0.125
S	S	1
		S 0.5
S	S	S 0,06
S	S	S 0.06
S	S	2
S	S	S1
S	S	S0,015
S	S	S 0.015
S	S	S0,03
		/
S	S	S 0.12
S	S	S 0.015
S	S	S 0.25
S	S	S 1
S	S	0,06
S	S	2
S	S	S0,03
S	S	S0,12
S	S	2
		S1
S	S	S2
S	S	I1
S	S	S 0.015
S	S	S 0.06
R	S	0,03
S	S	S 0.06
S	S	0,015
S	S	S 1
S	S	S 0.06
S	S	S 0.015

S	S	0,03
S	S	S 0.03
S	S	S<= 0.015
S	S	S 0.015
S	S	S 0.03
S	S	S 0.015
S	S	S 0.015
S	S	S0,015
S	S	/0.5
S	S	S 0.015
S	S	R 0.5
S	S	S0,06
S	S	S 0.12
S	S	S 0.015
S	S	S 0.03
S	S	S 0.06
S	S	0,06
S	S	S 0.03
S	S	S 2
S	S	R1
S	S	S0,06
S	S	S<=0,015
		S 0.015
S	S	S 0.5
S	S	S 1
S	S	S0,06
S	S	S<=0,015
S	S	S 0.06
S	S	S 2
S	S	S<=0,015
S	S	2
S	S	S 1
S	S	S 1
S	S	S 2
S	S	S 2
S	S	S 0,12
S	S	2
S	S	S0,12
S	S	S0,06
S	S	S0,03
S	S	S 0,015
S	S	S 0.06
S	S	S 0.12
S	S	S <=0.015
S	S	0,03
S	S	S 0.12
S	S	S 0.5

S	S	S 0.03
S	S	S 1
S	S	I1
S	S	S0,06
S	S	S0,12
S	S	S0,06
S	S	S 0.03
S	S	S0,12
S	S	S0,015
S	S	S 0.12
S	S	S<=0,015
S	S	0,06
S	S	S 0.03
S	S	S 0.015
S	S	S<=0.015
S	S	R1
S	S	S 0.015
S	S	S 0.03
S	S	0,06
		R 2
S	S	0,06
S	S	S 0.12
S	S	R 0.06
S	S	S0,12
S	S	S 0.015
S	S	S<= 0.015
S	S	S 0.25
S	S	S 0.015
S	S	S 0.015
S	S	S 0.015
S	S	0,03
S	S	S 0.015
S	S	1
S	S	S 0,015
S	S	S 0.5
S	S	S 1
S	S	S 0.03
S	S	S 0.06
S	S	S0,12
S	S	S <= 0.015
		S 0.06
S	S	<=0,015
S	S	S 1
S	S	S 0.03
S	S	0,06
		S 0.03

S	S	0,06
S	S	S0,12
S	S	S1
S	S	S 1
S	S	S 0.015
S	S	S0,12
S	S	S 0.5
S	S	S 0.015
S	S	2
S	S	S 0.12
S	S	S2
S	S	S2
S	S	S0,015
S	S	2
S	S	4
S	S	0,06
S	S	2
S	S	<=0,015
S	S	S0,06
		S0,5
S	S	
S	S	S0,015
S	S	S<= 0.015
S	S	S<= 0.015
S	S	S<=0.015
S	S	S 0.015
S	S	S 0.015
S	S	S0,015
S	S	0,015
S	S	S0,06
S	S	S 0.03
S	S	S 0.06
S	S	0,25
S	S	1
S	S	S0,06
S	S	S 0.015
S	S	S2
S	S	S 0.06
S	S	S 0.03
S	S	S 0.12
S	S	S<= 0.015
S	S	S 0.03
S	S	S 0.03
S	S	S0,015
S	S	/
S	S	S 0.03
S	S	S 0.12

S	S	S 0.015
S	S	S0,015
S	S	0,12
S	S	S<= 0.015
S	S	S0,03
S	S	S 0,015
S	S	S 0.015
S	S	S 0.015
S	S	0,015
S	S	S 0.03
S	S	I 2
S	S	S 0.03
S	S	R 1
S	S	R 2
S	S	0,25
S	S	S<=0,015
S	S	S0,03
S	S	S0,03
S	S	S0,12
S	S	0,015
S	S	S 0.06
S	S	S0,03
S	S	0,12
S	S	S0,015
S	S	S 1
S	S	S 0.015
S	S	0,12
S	S	S 0.03
		S0,06
S	S	S<= 0.015
S	S	2
S	S	S<=0,015
S	S	S0,06
S	S	S2
S	S	S<=0.015
S	S	S0,015
S	S	S2
S	S	2
S	S	S0,015
S	S	S 0.03
S	S	S 0.03
S	S	S<= 0.015
S	S	S 0.015
S	S	S0,06
S	S	I1
S	S	S0,03

		S 0.25
S	S	S 0.06
S	S	S0,015
S	S	S0,12
S	S	S<=0,015
S	S	0,03
S	S	S 0.06
S	S	S0,12
S	S	2
S	S	S0,12
S	S	S 0.06
S	S	S0,03
S	S	S2
S	S	S0,25
S	S	S0,03
S	S	S 0.03
S	S	S2
S	S	S 0.015
S	S	S 0.015
S	S	S0,015
S	S	S 0.03
		S0,03
S	S	S 0.5
S	S	S 1
S	S	S0,015
S	S	0,12
S	S	S2
S	S	S1
S	S	S0,03
S	S	0,03
S	S	S 0.015
S	S	S0,03
S	S	S0,06
S	S	S0,06
S	S	S 0.12
S	S	0,03
S	S	S0,12
S	S	S0,015
S	S	I0,12
S	S	S 0,03
S	S	S0,03
S	S	S 1
S	S	S 0.03
S	S	S 0,06
S	S	0,03
S	S	1

S	S	R 0.5
S	S	I 0.5
S	S	S 0.5
S	S	S0,03
S	S	S<= 0.015
S	S	R 2
S	S	S 0.015
S	S	S2
S	S	S 0.015
S	S	S 0.015
S	S	S 0.12
S	S	0,03
S	S	S 0,015
S	S	0,12
S	S	S 0.03
S	S	S0,03
S	S	S0,06
S	S	S0.03
S	S	0,06
S	S	S 0.015
S	S	S 0.015
S	S	S 2
S	S	S<=0,015
S	S	0,06
S	S	0,015
S	S	S 0.03
S	S	S0,12
S	S	S2
S	S	S 0.015
S	S	S<=0.015
S	S	<=0,015
S	S	2
S	S	S 0.03
S	S	S 0.015
S	S	S 2
S	S	0,06
S	S	S2
S	S	S 0.03
S	S	S 0.25
S	S	S0,015
S	S	S 0.015
S	S	S<= 0.015
S	S	S2
S	S	S<=0,015
S	S	2
S	S	0,03
S	S	I1

S	S	S 1
S	S	S 0.015
S	S	S 0.015
S	S	S 0.03
S	S	I0,5
S	S	S0,03
S	S	S 2
S	S	S 1
S	S	S 0.03
S	S	S<=0.015
S	S	R 0.5
S	S	S<=0,015
S	S	S0,015
S	S	S0,03
S	S	S<= 0.015
S	S	S 1
S	S	S 0.03
S	S	S0,03
S	S	S<= 0.015
S	S	S 0.5
S	S	S 0.03
S	S	S0,03
S	S	R 1
S	S	S 0.03
		S1
S	S	0,015
S	S	0,12
S	S	S 0.06
S	S	S0,03
S	S	S0,06
S	S	S 0.03
S	S	S0,06
S	S	1
S	S	S<=0,015
S	S	S 0.03
S	S	S<= 0.015
S	S	S0,015
S	S	S 0.03
S	S	S 0.06
S	S	S 0.015
S	S	S 0.06
S	S	S 0.015
S	S	S1
S	S	S 0.06
S	S	S 0.5
S	S	S 1
S	S	S 0.03

S	S	S 0.06
S	S	S0,25
S	S	2
S	S	S 0.03
S	S	S<=0,015
S	S	S 0,03
S	S	0,015
S	S	S0,03
S	S	S0,015
S	S	S0,06
S	S	S2
S	S	S 0.015
S	S	I1
S	S	S0,015
S	S	S 0.5
S	S	1
S	S	S<= 0.015
S	S	S<=0.015
S	S	S 0.03
S	S	S 0.25
S	S	S<= 0.015
S	S	S 1
S	S	S 0.015
S	S	S 0,06
S	S	S0,12
S	S	S1
S	S	S 0.015
S	S	S 1
S	S	S0,015
S	S	0,12
S	S	S<= 0.015
S	S	S <= 0.015
S	S	S 2
S	S	S 2
S	S	S 0.12
S	S	S0,03
S	S	S0,03
S	S	S 0.015
S	S	S1
		S 0.015
S	S	S 0.015
S	S	S 0.015
S	S	0,25
S	S	0,03
S	S	2
S	S	2
S	S	2

S	S	S 0.03
S	S	S 0.12
S	S	0,03
S	S	S2
S	S	S 0.12
S	S	S2
S	S	0,015
		S<=0,015
S	S	S 0.06
		R 0.5
S	S	S0,06
S	S	S1
S	S	S0,015
S	S	S 0.06
S	S	S1
S	S	S1
S	S	S 0.03
S	S	S <= 0.015
S	S	R 0.5
S	S	S <=0.015
S	S	S 0.03
S	S	0,06
S	S	S 0.03
S	S	1
S	S	<=0,005
S	S	S0,12
S	S	S 0.25
S	S	S0,5
S	S	S0,06
S	S	S 0.5
S	S	S 0.25
S	S	S 0.12
S	S	I2
S	S	<=0,015
S	S	2
S	S	0,5
S	S	S0,03
S	S	0,06
S	S	<=0,015
S	S	S 0.015
S	S	S<=0,015
S	S	S1
S	S	S0,015
S	S	S 0.12
S	S	S<0,015
S	S	S 0.015
S	S	S 0.03

**S**	**S**	S<= 0.015
**S**	**S**	S0,12
**S**	**S**	S 0.03
S	S	0,03
**S**	**S**	S0,12
**S**	**S**	S0,12
**S**	**S**	S0,12
**S**	**S**	S<= 0.015
S	S	0,015
S	S	0,125
S	S	0,25
**S**	**S**	S 0.03
**S**	**S**	S0,03
**S**	**S**	S1
**S**	**S**	S0,03
S	S	0,12
**S**	**S**	S 0.12

Susceptibility according to EUCAST (Anidulafungin)	Susceptibility according to CLSI (Anidulafungin)	MIC of Caspofungin
S	S	S0,03
S	S	S0,03
S	S	S 0.06
S	S	S 0.25
S	S	S 0.03
S	S	S 0.015
S	S	S 0.015
I	S	1
R	S	S 0.06
S	S	I0,06
R	S	S 0.03
S	S	0,25
I	S	0,5
R	S	S 0.125
I	S	0,5
		S 0.25
S	S	S0,06
R	S	S 0.06
I	S	0,25
S	S	S0,5
S	S	S0,03
S	S	S 0.06
S	S	S 0,06
		/
R	S	S 0.12
S	S	S 0.03
S	S	S 0.25
S	S	S 0.12
R	S	0,03
I	S	0,25
S	S	S0,03
R	S	S0,03
R	R	1
		S0,5
S	S	S0,5
S	S	I0,25
S	S	S 0.06
R	S	S 0.25
S	S	0,06
R	S	S 0.06
S	S	0,015
S	S	S 1
R	S	S 0.125
S	S	S 0.03

S	S	0,03
S	S	S 0.03
S	S	S 0.06
S	S	S 0.03
S	S	S 0.06
S	S	S 0.06
S	S	S 0.06
S	S	S0,015
S	S	/0.25
S	S	S 0.06
S	S	S 0.25
R	S	S 0.12
R	S	S 0.12
S	S	S 0.03
S	S	S 0.03
R	S	S 0.03
R	S	0,25
S	S	S 0.06
S	S	S 0.25
S	S	R1
S	S	S0,015
S	S	S0,003
		S 0.06
S	S	S 0.25
S	S	S 0.25
R	S	S0,06
S	S	S0,015
R	S	S 0.06
S	S	S 0.5
S	S	S0,06
I	S	0,5
S	S	S 1
S	S	S 0.5
S	S	S 1
S	S	S 0.5
R	S	S 0,06
I	S	0,25
R	S	S0,06
R	S	S0,03
S	S	S0,03
S	S	S 0,015
R	S	S 0.12
R	S	S 0.25
S	S	S 0.03
S	S	0,06
S	S	S 0.5
S	S	S 0.12

S	S	S 0.12
S	S	S 0.5
S	S	I0,25
R	S	S0,06
S	S	S0,06
S	S	S0,03
S	S	S 0.06
R	S	S0,06
S	S	S0,06
R	S	S 0.12
S	S	S0,03
R	S	0,06
S	S	S 0.06
S	S	S 0.03
S	S	S 0.03
S	S	S 0.5
S	S	S 0.03
S	S	S 0.03
R	S	0,03
		R 1
R	S	0,03
S	S	S 0.06
R	S	S 0.06
R	S	S0,06
S	S	S 0.06
S	S	S 0.06
S	S	S 0.25
S	S	S 0.06
S	S	S 0.06
S	S	S 0.015
S	S	0,06
S	S	S 0.03
I	S	0,25
S	S	S 0,03
S	S	S 0.25
S	S	S0,5
S	S	S 0.03
R	S	S 0.06
S	S	S0,12
S	S	S 0.03
		S 0.25
S	S	0,06
S	S	S 0.25
S	S	S 0.03
R	S	0,06
		S 0.25

S	S	0,25
S	S	S0,015
S	S	S 0,5
S	S	S 0.25
S	S	S 0.03
R	S	S0,06
S	S	S 0.5
S	S	S 0.06
I	S	0,5
R	S	S 0.06
S	S	S1
S	S	S0,5
S	S	S0,06
I	S	0,5
I	S	1
R	S	0,06
		0,5
S	S	0,03
R	S	S0,06
		S0,12
S	S	S0,03
S	S	S 0.03
S	S	S 0.03
S	S	S 0.03
S	S	S 0.03
S	S	S 0.06
S	S	S0,03
S	S	0,03
R	S	S0,03
S	S	S 0.03
R	S	S 0.03
		0,5
		8
R	S	S0,06
S	S	S 0.06
S	S	S0,25
S	S	S 0.06
S	S	S 0.06
R	S	S 0.12
S	S	S 0.015
S	S	S 0.03
S	S	S 0.06
S	S	S0,06
**nd**	**nd**	S0,25
S	S	S 0.06
S	S	S 0.06

S	S	S 0.03
S	S	S0,12
R	S	0,06
S	S	S 0.015
S	S	S0,06
S	S	S <= 0,008
S	S	S 0.03
S	S	S<=0.008
S	S	0,015
S	S	S 0.03
S	S	I 1
S	S	S 0.03
S	S	R1
S	S	R 1
R	S	0,06
S	S	S0,03
S	S	S1
S	S	S0,06
S	S	S0,06
S	S	0,03
S	S	S 0.06
S	S	S0,06
R	S	0,06
S	S	S0,06
S	S	S 0.25
S	S	S 0.03
R	S	0,06
S	S	S 0.03
		S0,06
S	S	S 0.06
I	S	0,5
S	S	S0,03
**R**	S	S0,03
S	S	S0,12
S	S	S 0.03
S	S	S0,12
S	S	S0,5
I	S	0,5
S	S	S0,03
S	S	S 0.03
S	S	S 0.06
S	S	S 0.03
S	S	S 0.03
S	S	S0,12
S	S	I0,25
S	S	S0,06

		S 0.25
**R**	**S**	S 0.12
**S**	**S**	S0,015
**R**	**S**	S0,06
**S**	**S**	S0,03
S	S	0,12
**R**	**S**	S 0.12
**R**	**S**	S0,06
I	S	0,5
**R**	**S**	S0,03
**S**	**S**	S 0.06
**S**	**S**	S0,06
**S**	**S**	S0,5
**R**	**S**	S0,25
**S**	**S**	S0,06
**S**	**S**	S 0.03
**S**	**S**	S0,5
**S**	**S**	S 0.06
**S**	**S**	S 0.03
**S**	**S**	S0,03
**S**	**S**	S < 0.08
		S0,06
**S**	**S**	S 0.25
**S**	**S**	S 0.25
**S**	**S**	S0,06
R	S	0,03
**S**	**S**	S0,25
**S**	**S**	S0,25
**S**	**S**	S0,06
S	S	0,06
**S**	**S**	S 0.12
**S**	**S**	S0,06
**R**	**S**	S0,06
**R**	**S**	S0,06
**S**	**S**	S 0.06
S	S	0,015
**S**	**S**	S0,06
**S**	**S**	S0,03
**S**	**S**	I0,12
**S**	**S**	S 0,06
**S**	**S**	S0,015
**S**	**S**	S 0.25
**S**	**S**	S 0.03
**R**	**S**	S0,06
S	S	0,03
		0,25

	S	S	S 0.25
	S	S	I 0.5
	S	S	S 0.25
	S	S	S0,12
	S	S	S 0.06
	S	S	S 0.25
	S	S	S 0.03
	S	S	S0,5
	S	S	S 0.03
	S	S	S 0.03
	R	S	S 0.06
	S	S	0,06
	S	S	S 0,015
	R	S	0,06
	S	S	S 0.03
	S	S	S2
	R	S	S0,06
	S	S	S0.06
	R	S	0,06
	S	S	S 0.03
	S	S	S 0.06
	S	S	S 0.5
	S	S	S0,03
	R	S	0,03
	S	S	0,015
	S	S	S 0.06
	R	S	S0,06
	S	S	S0,25
	S	S	S 0.03
	S	S	S 0.06
	S	S	0,015
	I	S	0,5
	S	S	S 0.06
	S	S	S 0.015
	S	S	S 0,25
	R	S	0,06
	S	S	S1
	S	S	S 0.03
	S	S	S 0.25
	S	S	S0,06
	S	S	S 0.06
	S	S	S 0.06
	S	S	S0,25
	S	S	S 0,06
	I	S	0,5
	S	S	0,03
	S	S	I0,25

S	S	S 0.25
S	S	S 0.03
S	S	S 0.06
S	S	S 0.03
S	S	I0,25
S	S	S0,06
S	S	S 0.5
S	S	S 0.5
S	S	S 0.06
S	S	S 0.03
S	S	S 0.25
S	S	S0,03
S	S	S0,06
S	S	S0,03
S	S	S<= 0.06
S	S	S 0.5
S	S	S 0.03
S	S	S0,06
S	S	S 0.03
S	S	S 0.25
S	S	S 0.12
S	S	S0,015
S	S	S 0.25
S	S	S 0.03
		S0,5
S	S	0,06
R	S	0,5
S	S	S 0.06
S	S	S0,12
R	S	S0,06
S	S	S 0.03
R	S	S0,12
I	S	0,5
S	S	S0,015
S	S	S 0.03
S	S	S 0.03
S	S	S0,015
S	S	S 0.06
S	S	S 0.06
S	S	S 0.03
S	S	S 0.06
S	S	S 0.03
S	S	S0,5
S	S	S 0.06
S	S	S 1
S	S	S 0.25
S	S	S 0.03

S	S	S 0.06
S	S	S0,5
I	S	1
S	S	S 0.06
S	S	S0,03
S	S	S 0,03
S	S	0,03
S	S	S0,06
S	S	S0,03
S	S	S0,06
S	S	S1
S	S	S<= 0.008
S	S	I0,5
S	S	S0,03
S	S	S 0.25
I	S	0,5
S	S	S 0.03
S	S	S 0.03
S	S	S 0.06
R	S	S 0.06
S	S	S 0.03
S	S	S 0.25
S	S	S 0.03
R	S	S 0,03
R	S	S0,12
S	S	S0,5
S	S	S 0.06
S	S	S 1
S	S	S0,03
R	S	0,06
S	S	S 0.03
S	S	S 0.06
S	S	S 0.5
S	S	S 0.5
S	S	S 0.12
S	S	S0,03
S	S	S0,03
S	S	S 0.06
S	S	S0,25
		S 0.008
S	S	S 0.03
S	S	S 0.03
		0,12
S	S	0,06
I	S	0,5
I	S	0,5
I	S	0,5

S	S	S 0.12
R	S	S 0.12
S	S	0,03
S	S	S0,5
R	S	S 0.12
S	S	S0,5
S	S	0,03
		S<=0,008
R	S	S 0.06
		S 0.12
R	S	S0,06
S	S	S0,5
S	S	S0,03
R	S	S 0.12
S	S	S0,5
S	S	S0,5
S	S	S 0.03
S	S	S 0.015
S	S	S 0.5
S	S	S 0.03
S	S	S 0.03
R	S	0,03
S	S	S 0.06
I	S	0,5
S	S	0,03
R	S	S0,03
S	S	S 0.06
S	S	S0,25
R	S	S0,06
S	S	S 0.25
S	S	S 0.25
S	S	S 0.06
S	S	I0,25
S	S	0,06
I	S	0,5
I	S	0,5
S	S	S0,12
R	S	0,03
S	S	0,03
S	S	S 0.03
S	S	S0,03
S	S	S0,5
S	S	S0,03
R	S	S 0.12
S	S	S0,03
S	S	S 0.06
S	S	S 0.03

**S**	**S**	S 0.015
**R**	**S**	S0,03
**S**	**S**	S 0.03
S	S	0,25
**S**	**S**	S0,06
**S**	**S**	S0,06
**R**	**S**	S0,12
**S**	**S**	S 0.03
S	S	0,015
R	R	0,5
R	R	0,5
**S**	**S**	S 0.03
**S**	**S**	S0,03
**S**	**S**	S0,5
**S**	**S**	S0,06
		0,015
**S**	**S**	S 0.06

Susceptibility according to EUCAST (Caspofungin)	Susceptibility according to CLSI (Caspofungin)	MIC of Fluconazole
**nd**	**S**	I8
**nd**	**S**	S1
**nd**	**S**	S 2
**nd**	**S**	S 0.5
**nd**	**S**	S <0.12
**nd**	**S**	S<=0.12
**nd**	**S**	S<= 0.12
	S	0,5
**nd**	**S**	S 0.25
**nd**	**S**	S0,5
**nd**	**S**	S 0.5
**nd**	S	>64
	S	0,5
**nd**	**S**	S 1
	S	1
		/ 4
**nd**	**S**	S1
**nd**	**S**	S 0.25
	S	0,06
**nd**	**S**	I 0,4
**nd**	**S**	S<=0,12
**nd**	**S**	S 8
**nd**	**S**	S0,25
		R 8
**nd**	**S**	S 0.25
**nd**	**S**	S 0.25
**nd**	**S**	S 0.12
**nd**	**S**	S 0.25
**nd**	S	0,25
	S	0,5
**nd**	**S**	S0,5
**nd**	**S**	S0,5
**nd**	R	128
		/4
**nd**	**S**	S1
**nd**	**S**	S0,5
**nd**	**S**	S 0.25
**nd**	**S**	S 0.25
	S	32
**nd**	**S**	S 0.12
**nd**	S	1
**nd**	**S**	S 0.25
**nd**	**S**	S 1
**nd**	**S**	S 0.5

nd	S	0,25
nd	S	S 0.12
nd	S	S 2
nd	S	S 0.25
nd	S	S 0.25
nd	S	S 0.25
nd	S	S 0.25
nd	S	S0,25
nd	S	S 0.25
nd	S	S 0.25
nd	S	S 0.25
nd	S	S 0.5
nd	S	S 0.25
nd	S	S 0.25
nd	S	S 0.25
nd	S	S 0.25
nd	S	0,25
nd	S	I 16
nd	S	S 0.5
nd	S	S1
nd	S	S1
nd	S	I16
		S 4
nd	S	S 2
nd	S	S 0.5
nd	S	S0,25
nd	S	S0,5
nd	S	S 0.5
nd	S	S 0.5
nd	S	S0,25
	S	0,5
nd	S	S 0.25
nd	S	S 0.5
nd	S	S 0.25
nd	S	S.025
nd	S	S 2
	S	0,5
nd	S	S1
nd	S	S0,5
nd	S	S1
nd	S	S0,5
nd	S	S 0.5
nd	S	S<=0.12
nd	S	S 0.25
nd	S	1
nd	S	S<=0.06
nd	S	S 0.25

nd	S	I 8
nd	S	S 0.25
nd	S	S0,5
nd	S	S0,5
nd	S	S1
nd	S	I4
nd	S	R 32
nd	S	S0,5
nd	S	S2
nd	S	S 0.25
nd	S	S0,25
nd	S	0,25
nd	S	S 0.5
nd	S	S 0.5
nd	S	S 2
nd	S	S1
nd	S	S 0.5
nd	S	S 1
nd	S	0,25
		S 2
nd	S	0,5
nd	S	S 1
nd	S	S 0.5
nd	S	S0,25
nd	S	S 0.25
nd	S	I 8
nd	S	S 2
nd	S	I 8
nd	S	S 0.25
nd	S	S <0.12
nd	S	0,12
nd	S	S 0.25
	S	0,5
nd	S	S 0,5
nd	S	S 0.5
nd	S	S1
nd	S	S 2
nd	S	S 0.25
nd	S	S1
nd	S	S 0.25
		R 64
	S	8
nd	S	S 0.5
nd	S	S 0.25
nd	S	0,5
		R

	S	/
**nd**	**S**	S1
**nd**	**S**	S 0,12
**nd**	**S**	S 0.5
**nd**	**S**	S 0.25
**nd**	**S**	S0,25
**nd**	**S**	S 0.12
**nd**	**S**	S <0.12
	S	1
**nd**	**S**	S 0.12
**nd**	**S**	S1
**nd**	**S**	S0,5
**nd**	**S**	S0,25
	S	0,25
	S	0,25
**nd**	S	0,25
		2
**nd**	S	1
**nd**	**S**	S1
		/2
**nd**	**S**	S0,25
**nd**	**S**	S 0.25
**nd**	**S**	S 0.25
**nd**	**S**	S 0.25
**nd**	**S**	I 4
**nd**	**S**	R 8
**nd**	**S**	S0,25
	S	8
**nd**	**S**	S0,5
**nd**	**S**	S 1
**nd**	**S**	S 1
		1
		1
**nd**	**S**	S0,25
**nd**	**S**	S < 0.12
**nd**	**S**	S0,5
**nd**	**S**	S 0.5
**nd**	**S**	S 1
**nd**	**S**	S 0.5
**nd**	**S**	S 0.25
**nd**	**S**	S 0.25
**nd**	**S**	I 8
**nd**	**S**	S0,5
**nd**	**S**	S0.25
**nd**	**S**	R>=256
**nd**	**S**	S 2

**nd**	**S**	S 0.25
**nd**	**S**	I8
**nd**	S	0,5
**nd**	**S**	S 0.25
**nd**	**S**	S0,5
**nd**	**S**	S 0,25
**nd**	**S**	S 0.5
**nd**	**S**	S 1
**nd**	S	0,5
**nd**	**S**	R 8
**nd**	**S**	S 0,5
**nd**	**S**	S 0.25
**nd**	**S**	S 1
**nd**	**S**	S 0.5
**nd**	S	0,5
**nd**	**S**	S0,5
**nd**	**nd**	**nd**
**nd**	**S**	I16
**nd**	**S**	R8
**nd**	S	0,5
**nd**	**S**	I 8
**nd**	**S**	S0,25
**nd**	S	0,25
**nd**	**S**	S0,25
**nd**	**S**	S 0.5
**nd**	**S**	S 0.25
**nd**	S	0,12
**nd**	**S**	S 0.25
		S0,25
**nd**	**S**	S 0.5
	S	0,5
**nd**	**S**	S0,5
**nd**	**S**	S0,5
**nd**	**S**	S1
**nd**	**S**	S 0.25
**nd**	**S**	R16
**nd**	**S**	S0,5
	S	1
**nd**	**S**	S0,5
**nd**	**S**	S 0.25
**nd**	**S**	I 16
**nd**	**S**	S 2
**nd**	**S**	S 0.25
**nd**	**S**	S1
**nd**	**S**	S1
**nd**	**S**	I4

		/ 2
nd	S	S 0.25
nd	S	S0,25
nd	S	S0,25
nd	S	S0,5
nd	S	0,25
nd	S	S 1
nd	S	S0,25
	S	0,06
nd	S	S0,5
nd	S	R 8
nd	S	I8
nd	S	S0,25
nd	S	I4
nd	S	I 16
nd	S	S 0.5
nd	S	S0,25
nd	S	S 0.25
nd	S	S 1
nd	S	s<=0,12
nd	S	S 0.5
		S0,12
nd	S	S 0.5
nd	S	S 0.5
nd	S	S0,5
nd	S	0,5
nd	S	S1
nd	S	S1
nd	S	S0,5
	S	32
nd	S	I 16
nd	S	S32
nd	S	S0,5
nd	S	S0,5
nd	S	S 2
nd	S	0,25
nd	S	S2
nd	S	S0,25
nd	S	S1
nd	S	S0,5
nd	S	S0,5
nd	S	S 4
nd	S	R > 256
nd	S	S0,5
nd	S	0,25
		4

**nd**	**S**	S 0.5
**nd**	**S**	S 0.25
**nd**	**S**	S 0.25
**nd**	**S**	/8
**nd**	**S**	I 8
**nd**	**S**	S 0.5
**nd**	**S**	S 0.25
**nd**	**S**	S0,5
**nd**	**S**	S 0.25
**nd**	**S**	S 0.25
**nd**	**S**	S 0.25
**nd**	S	0,25
**nd**	**S**	S 0,25
	S	4
**nd**	**S**	S 0.25
**nd**	**R**	S2
**nd**	**S**	S0,5
**nd**	**S**	S1
**nd**	S	1
**nd**	**S**	S<=0.12
**nd**	**S**	I 8
**nd**	**S**	S 0.25
**nd**	**S**	S0,25
**nd**	S	0,25
**nd**	S	1
**nd**	**S**	S 0.25
**nd**	**S**	S1
**nd**	**S**	S0,5
**nd**	**S**	S 0.25
**nd**	**S**	S<= 0.12
**nd**	S	0,25
	S	1
**nd**	**S**	R 64
**nd**	**S**	S <0.12
**nd**	**S**	S 2
**nd**	S	0,5
**nd**	**S**	S1
**nd**	**S**	S 0.25
**nd**	**S**	S 0.25
**nd**	**S**	S0,5
**nd**	**S**	S 0.25
**nd**	**S**	S<= 0.12
**nd**	**S**	S0,5
**nd**	**S**	I8
	S	0,5
**nd**	S	0,5
**nd**	**S**	S0,25

**nd**	S	S 0.5
**nd**	S	R>256
**nd**	S	S 0.12
**nd**	S	S 2
**nd**	S	S1
**nd**	S	I4
**nd**	S	S 0.5
**nd**	S	S 0.5
**nd**	S	S < 0.12
**nd**	S	S 0.25
**nd**	S	S 0.5
**nd**	S	S0,25
**nd**	S	I16
**nd**	S	S0,25
**nd**	S	S<= 0.12
**nd**	S	S 0.5
**nd**	S	I 4
**nd**	S	S0,25
**nd**	S	S<= 0.12
**nd**	S	S 1
**nd**	S	S 0.25
**nd**	S	S2
**nd**	S	S 0.25
**nd**	S	S 0.25
		S
**nd**	S	0,5
**nd**	S	0,5
**nd**	S	S 2
**nd**	S	R>=128
**nd**	S	S0,25
**nd**	S	S 0.25
**nd**	S	S0,5
	S	0,5
**nd**	S	S0,12
**nd**	S	S 0.5
**nd**	S	I 4
**nd**	S	S0,25
**nd**	S	I 16
**nd**	S	I 16
**nd**	S	S<=0.12
**nd**	S	S 1
**nd**	S	S 0.25
**nd**	S	S1
**nd**	S	I 16
**nd**	S	S 0.5
**nd**	S	S 0.5
**nd**	S	S 1

**nd**	**S**	S 1
**nd**	**S**	S1
	S	1
**nd**	**S**	S 0.25
**nd**	**S**	S0,5
**nd**	**S**	S 0,25
**nd**	S	0,25
**nd**	**S**	S0,5
**nd**	**S**	S0,5
**nd**	**S**	S2
**nd**	**S**	S0,5
**nd**	**S**	S 0.12
**nd**	**S**	S0,05
**nd**	**S**	S0,5
**nd**	**S**	S 0.5
	S	0,5
**nd**	**S**	S 0.25
**nd**	**S**	S 0.25
**nd**	**S**	S 2
**nd**	**S**	R>256
**nd**	**S**	S 0.5
**nd**	**S**	S 0.5
**nd**	**S**	S 0.5
**nd**	**S**	S 0,25
**nd**	**S**	S0,5
**nd**	**S**	S1
**nd**	**S**	S 0.25
**nd**	**S**	S 0.5
**nd**	**S**	S0,5
**nd**	S	0,25
**nd**	**S**	I 8
**nd**	**S**	S 0.5
**nd**	**S**	S 1
**nd**	**S**	S 1
**nd**	**S**	S 0.25
**nd**	**S**	S0,25
**nd**	**S**	S0,25
**nd**	**S**	S 0.25
**nd**	**S**	S0,5
		S 0.5
**nd**	**S**	S <0.12
**nd**	**S**	S <0.12
		0,5
	S	32
	S	0,5
	S	0,5
	S	0,5

**nd**	**S**	I 8
**nd**	**S**	S 0.12
	S	16
**nd**	**S**	S0,25
**nd**	**S**	S <=0.12
**nd**	**S**	S1
**nd**	S	0,5
		S1
**nd**	**S**	S 0.25
		S1
**nd**	**S**	S0,5
**nd**	**S**	S0.5
**nd**	**S**	S0,5
**nd**	**S**	S<= 0.25
**nd**	**S**	S0,25
**nd**	**S**	S0,5
**nd**	**S**	S 1
**nd**	**S**	S <=0.12
**nd**	**S**	S 1
**nd**	**S**	S 0.5
**nd**	**S**	S 0.25
**nd**	S	0,25
**nd**	**S**	S 0.5
	S	1
**nd**	S	0,5
**nd**	**S**	S<=0,012
**nd**	**S**	S1
**nd**	**S**	S0,25
**nd**	**S**	S0,5
**nd**	**S**	S 0.5
**nd**	**S**	S 1
**nd**	**S**	S 8
**nd**	**S**	S0,25
	S	16
	S	0,5
	S	0,5
**nd**	**S**	R64
**nd**	S	0,5
**nd**	S	0,12
**nd**	**S**	S 0.5
**nd**	**S**	S<=0,12
**nd**	**S**	S1
**nd**	**S**	S0,25
**nd**	**S**	S 0.5
**nd**	**S**	S0,25
**nd**	**S**	S 0.5
**nd**	**S**	S 0.5

**nd**	**S**	S 0.5
**nd**	**S**	S0,5
**nd**	**S**	S 0.5
**nd**	S	0,12
**nd**	**S**	S1
**nd**	**S**	S1
**nd**	**S**	S0,25
**nd**	**S**	S 2
**nd**	S	0,5
	S	>32
	S	32
**nd**	**S**	S 0.25
**nd**	**S**	S0,5
**nd**	**S**	S0,5
**nd**	**S**	S0,5
		0,25
**nd**	**S**	S 0.5

Susceptibility according to EUCAST (Fluconazole)	Susceptibility according to CLSI (Fluconazole)	MIC of Micafungin
S	S	S0,015
S	S	S0,015
S	S	S 0.03
S	S	S 2
S	S	S < 0.008
S	S	S<=0.008
S	S	S<= 0.008
S	S	2
S	S	S <= 0.008
S	S	I1
S	S	S <= 0.008
R	R	0,015
S	S	1
S	S	S 0.03
S	S	1
		S 0.25
S	S	S0,015
S	S	S 0.008
S	S	2
S	S	S1
S	S	S<=0,008
S	S	S 0.015
S	S	S<=0,008
		/
S	S	S<0.008
S	S	S <= 0.008
S	S	S 0.25
S	S	S 0.5
S	S	0,015
S	S	1
S	S	S<=0,008
S	S	S0,015
R	R	2
		S0,5
S	S	S2
S	S	I1
S	S	S 0.015
S	S	S 0.015
R	S	0,015
S	S	S < 0.008
S	S	0,008
S	S	S 1
S	S	S 0.03
S	S	S 0.015

S	S	0,015
S	S	S<=0.008
S	S	S<=0.008
S	S	S <= 0.008
S	S	S <= 0.008
S	S	S 0.015
S	S	S 0.015
S	S	S0,015
S	S	/1
S	S	S<= 0.008
S	S	R 1
S	S	S 0.015
S	S	S 0.015
S	S	S<=0,008
S	S	S<= 0.008
S	S	S 0.008
S	S	0,015
S	S	S 0.015
S	S	S 2
S	S	R1
S	S	S0,03
S	S	S0,015
		S 0.008
S	S	S 0.5
S	S	S 1
S	S	S0,01
S	S	S0,015
S	S	S 0.008
S	S	S 1
S	S	S0,015
S	S	2
S	S	S 1
S	S	S 2
S	S	S 2
S	S	S 2
S	S	S 0,03
S	S	1
S	S	S0,015
S	S	S0,008
S	S	S0,015
S	S	S<=0,008
S	S	S 0.015
S	S	S 0.015
S	S	S <= 0.008
S	S	<=0,008
S	S	S 0.5
S	S	S 0.5

S	S	S 0.015
S	S	S 1
S	S	I1
S	S	S0,008
S	S	S0,5
S	S	S0,03
S	S	S 0.015
S	S	S0,015
S	S	S0,015
S	S	S<0.008
S	S	S<=0,008
S	S	<=0,008
S	S	S 0.015
S	S	S 0.015
S	S	S <=0.015
S	S	S1
S	S	S <=0.008
S	S	S 0.03
S	S	<=0,008
		R 1
S	S	0,008
S	S	S 0.12
S	S	S0,015
S	S	S0,015
S	S	S <= 0.008
S	S	S 0.015
S	S	I 0.5
S	S	S<= 0.008
S	S	S<=0.008
S	S	S<=0,008
S	S	0,03
S	S	S0,008
S	S	1
S	S	S <=0,008
S	S	S 1
S	S	S1
S	S	S 0.015
S	S	S 0.008
S	S	S0,03
S	S	S <= 0.008
		S 0.125
R	S	<=0,008
S	S	S 1
S	S	S 0.03
S	S	0,015
		S 0.12

		0,125
S	S	S0,03
S	S	S1
S	S	S 0.5
S	S	S<=0.008
S	S	S0,015
S	S	S 0.5
S	S	S<=0,008
S	S	2
S	S	S 0.015
S	S	I 4
S	S	S2
S	S	S0,015
S	S	1
S	S	4
S	S	<=0,015
		0,5
S	S	<=0,008
S	S	S0,015
		S0,125
S	S	S<=0,008
S	S	S<= 0.008
S	S	S<= 0.008
S	S	S<=0.008
S	S	S 0.03
S	S	S 0.015
S	S	S0,015
R	S	0,015
S	S	S0,015
S	S	S 0.015
S	S	S 0.015
		0,125
		2
S	S	S0,015
S	S	S 0.015
S	S	S2
S	S	S 0.03
S	S	S 0.03
S	S	S 0.008
S	S	S<= 0.008
S	S	S <= 0.008
S	S	S 0.015
S	S	S0,015
S	S	/
R	R	S<=0.015
S	S	S 0.06

S	S	S<=0.008
S	S	S0,015
S	S	0,015
S	S	S<= 0.008
S	S	S<=0,008
S	S	S 0,015
S	S	S 0.015
S	S	S<=0.008
S	S	0,015
R	R	S 0.015
S	S	I 2
S	S	S<=0.008
S	S	R 1
S	S	R 1
S	S	0,015
S	S	S<=0,008
**nd**	**nd**	**nd**
R	R	S0,015
R	R	S0,06
S	S	0,015
S	S	S 0.015
S	S	S0,008
S	S	0,008
S	S	S<=0,008
S	S	S 1
S	S	S<= 0.008
S	S	0,015
S	S	S 0.015
		S0,06
S	S	S<=0,008
S	S	1
S	S	S<=0,008
S	S	S0,015
S	S	S1
S	S	S<=0.008
S	S	S0,015
S	S	S1
S	S	2
S	S	S0,015
S	S	S<=0,008
S	S	S 0.016
S	S	S<=0.008
S	S	S 0.015
S	S	S0,03
S	S	I1
S	S	S0,015

		S 0.25
S	S	S<=0,008
S	S	S<=0,008
S	S	S0,015
S	S	S<=0,008
S	S	0,015
S	S	S<0.008
S	S	S0,015
S	S	2
S	S	S0,015
R	R	S 0.03
S	S	S0,015
S	S	S2
S	S	S0,06
S	S	S0,015
S	S	S 0.015
S	S	S2
S	S	S<= 0.008
S	S	S 0.015
S	S	S0,015
S	S	S<=0,008
		S0,015
S	S	S 0.5
S	S	S 1
S	S	S0,015
S	S	0,08
S	S	S2
S	S	S1
S	S	S<=0,008
R	S	0,015
S	S	S 0.015
S	S	S0,03
S	S	S<0,008
S	S	S0,015
S	S	S 0.06
S	S	<=0,008
S	S	S0,06
S	S	S<=0,008
S	S	I0,25
S	S	S0,015
S	S	S0,008
S	S	S 1
R	R	S 0.015
S	S	S<=0,008
S	S	<=0,008
		0,5

S	S	S 0.5
S	S	R 1
S	S	S 1
S	S	S0,03
S	S	S 0.015
S	S	R 2
S	S	S<= 0.008
S	S	S2
S	S	S 0.008
S	S	S<=0.008
S	S	S 0.015
S	S	0,015
S	S	S<=0,008
I	I	0,03
S	S	S<=0.008
S	S	S0,015
S	S	S<=0,008
S	S	S0,015
S	S	0,03
S	S	S<=0.008
S	S	S<=0.008
S	S	S 1
S	S	S0,015
S	S	<=0,008
S	S	0,015
S	S	S 0.008
S	S	S0,015
S	S	S1
S	S	S 0.008
S	S	S<=0.008
S	S	0,015
S	S	2
R	R	S 0.015
S	S	S<=0,008
S	S	S 2
S	S	<=0,008
S	S	S2
S	S	S<=0.008
S	S	S 0.25
S	S	S0,015
S	S	S 0.015
S	S	S<= 0.008
S	S	S2
S	S	S 0,015
S	S	1
S	S	<=0,008
S	S	I0,5

S	S	S 1
R	R	S<=0.008
S	S	S<=0.008
S	S	S 0.06
S	S	I0,5
S	S	S0,015
S	S	S 2
S	S	S 1
S	S	S 0.015
S	S	S<=0.008
S	S	R 1
S	S	S<=0,008
S	S	S0,015
S	S	S<=0,008
S	S	S<= 0.008
S	S	S 0.5
S	S	S 0.03
S	S	S<=0,008
S	S	S<= 0.008
S	S	S 0.5
S	S	S 0.015
S	S	S0,03
S	S	R 1
S	S	S<= 0.008
		S0,5
S	S	0,015
S	S	0,015
S	S	S 0.06
R	R	S0,015
S	S	S0,008
S	S	S <= 0.008
S	S	S0,03
S	S	2
S	S	S<=0,008
S	S	S <= 0.008
S	S	S 0.015
S	S	S0,015
S	S	S<=0.008
S	S	S < 0.008
S	S	S<=0.008
S	S	S 0.03
S	S	S <= 0.008
S	S	S1
S	S	S<= 0.008
S	S	S 1
S	S	S 1
S	S	S 0.015

S	S	S 0.06
S	S	S0,25
S	S	2
S	S	S 0.03
S	S	S0,015
S	S	S<=0,008
S	S	<=0,008
S	S	S0,015
S	S	S<=0,008
S	S	S0,03
S	S	I4
S	S	S<= 0.008
S	S	I1
S	S	S0,008
S	S	S 1
S	S	1
S	S	S<= 0.008
S	S	S<=0.008
S	S	S0,015
R	R	S 0.015
S	S	S<= 0.008
S	S	S 1
S	S	S 0.015
S	S	S <=0,008
S	S	S0,015
S	S	S2
S	S	S <=0.008
S	S	S 2
S	S	S0,03
S	S	0,015
S	S	S<= 0.008
S	S	S 0.015
S	S	S 2
S	S	S 2
S	S	S 0.12
S	S	S<=0,008
S	S	S0,008
S	S	S<= 0.008
S	S	S1
		S<=0.008
S	S	S <= 0.008
S	S	S <= 0.008
		0,06
R	S	0,15
S	S	2
S	S	2
S	S	2

S	S	S 0.015
S	S	S<0.008
R	S	0,015
S	S	S2
S	S	S<=0.008
S	S	S2
S	S	0,015
		S<=0,008
S	S	S<=0.008
		S 0.25
S	S	S<=0,008
S	S	S1
S	S	S0,015
S	S	S 0.06
S	S	S1
S	S	S2
S	S	S 0.03
S	S	S<= 0.015
S	S	R 1
S	S	S<=0.008
S	S	S 0.015
S	S	0,015
S	S	S 0.008
S	S	1
S	S	<=0,008
S	S	S0,015
S	S	S 0.125
S	S	S0,5
S	S	S0,015
S	S	S 1
S	S	S 0.5
R	R	S 0.06
S	S	I1
R	S	<=0,015
S	S	2
S	S	1
R	R	S0,015
S	S	0,015
S	S	<=0,008
S	S	S<0.008
S	S	S<=0,008
S	S	S2
S	S	S<=0,008
S	S	S<=0,008
S	S	S<0,008
S	S	S 0.03
S	S	S 0.008

S	S	S<= 0.008
S	S	S<=0,008
S	S	S<= 0.008
S	S	0,015
S	S	S0,03
S	S	S0,03
S	S	S0,015
S	S	S <=0.008
S	S	<=0,008
R	R	0,25
R	S	0,25
S	S	S<= 0.008
S	S	S<=0,008
S	S	S1
S	S	S0,015
		0,03
S	S	S 0.03

Susceptibility according to EUCAST (Micafungin)	Susceptibility according to CLSI (Micafungin)	MIC of Voriconazole
S	S	/0,12
S	S	S0,008
nd	S	S 0.12
S	S	S<= 0.008
S	S	S < 0.008
S	S	S<=0.008
S	S	S<= 0.008
R	S	0,03
S	S	S 0.015
S	S	S0,015
S	S	S<=0.008
S	S	0,008
R	S	0,015
nd	S	S 0.03
R	S	0,015
		/ 0.06
nd	S	S<=0,008
S	S	S <= 0.008
R	S	0,015
S	S	S0,12
S	S	S<=0,008
S	S	S 0.12
S	S	S<=0,0008
		/
S	S	S<0.008
S	S	S <= 0.008
S	S	S<=0.008
S	S	S<=0.008
S	S	<=0,008
R	S	<=0,008
S	S	S<=0,008
S	S	S0,008
R	R	1
		/0,25
S	S	S0,015
S	S	S0,008
S	S	S 0.008
S	S	S 0.015
S	S	
S	S	S < 0.008
S	S	0,03
S	S	S 0.008
R	S	S 0.015
S	S	S < 0.008

S	S	<=0,008
S	S	S<=0.008
S	S	S 0.06
S	S	S <= 0.008
S	S	S <= 0.008
S	S	S<= 0.008
S	S	S<= 0.008
S	S	S<=0,008
S	S	S<=0.008
S	S	S<= 0.008
S	S	S <= 0.008
S	S	S < 0.008
S	S	S<=0.008
S	S	S <=0.008
S	S	S<=0,008
S	S	S 0.008
S	S	0,008
S	S	
S	S	S 0.008
S	S	S0,03
**nd**	S	S0,12
S	S	n.d.
		S 0.12
S	S	S 0.06
S	S	S 0.015
S	S	S0,008
S	S	S<=0,008
S	S	S 0.008
S	S	S 0.015
S	S	S<=0,008
R	S	0,015
S	S	S 0.008
S	S	S 0.008
S	S	S<= 0.08
S	S	S<= 0.008
**nd**	S	I 0,25
R	S	<=0,008
S	S	S0,12
S	S	S0,008
S	S	S0,015
S	S	S<=0,008
S	S	S 0.03
S	S	S<=0.008
S	S	S <= 0.008
S	S	0,015
S	S	S<=0.008
S	S	S<=0.008

S	S	/ 1
S	S	S<=0.008
S	S	S<=0,008
S	S	0,015
S	S	S0,015
**nd**	S	S0,5
S	S	
S	S	S0,015
S	S	S0,06
S	S	S<=0.008
S	S	S<=0,008
S	S	0,008
S	S	S< 0.008
S	S	S<=0.008
S	S	S 0.06
S	S	S 0.015
S	S	S <=0.008
**nd**	S	S 0.06
S	S	0,015
		S 0.06
S	S	0,008
S	S	S<=0.008
S	S	S 0.015
S	S	S<=0,008
S	S	S <= 0.008
S	S	
S	S	S 0.06
S	S	
S	S	S<=0.008
S	S	S <0.008
R	S	0,008
S	S	S<=0.008
R	S	0,015
S	S	S0,06
S	S	S 0.015
S	S	S0,015
**nd**	S	S 0.12
S	S	S< 0.008
**nd**	S	S0,12
S	S	S 0.015
		S 0.5
S	S	
S	S	S 0.015
**R**	S	S <= 0.008
S	S	0,008
		S 0.25

	S	0,25
nd	S	I0,25
S	S	S0,03
S	S	S 0.015
S	S	S<=0.008
S	S	S<=0,008
S	S	S<=0.008
S	S	S< 0.008
R	S	0,03
S	S	S 0.008
R	S	S0,03
S	S	S0,015
S	S	S0,015
R	S	<=0,008
R	I	<=0,008
S	S	0,015
		0,12
S	S	
S	S	S0,03
		/0,06
S	S	S<=0,008
S	S	S 0.008
S	S	S<= 0.008
S	S	S<=0.008
nd	S	I 0.25
S	S	S 0.25
S	S	S0,015
S	S	
S	S	S<=0,008
nd	S	S 0.06
S	S	S 0.03
		0,015
		0,015
S	S	S0,015
S	S	S < 0.008
S	S	S0,015
nd	S	S 0.03
nd	S	S 0.06
S	S	S 0.008
S	S	S<= 0.008
S	S	S <= 0.008
S	S	/ 0.12
S	S	S<=0,008
nd	nd	S<=0,008
S	S	R>=8
nd	S	S 0.12

S	S	S 0.008
S	S	/
S	S	0,015
S	S	S<= 0.008
S	S	S0,015
S	S	S 0,008
S	S	S 0.008
nd	S	S 0.03
S	S	0,015
nd	S	I 0.5
S	S	S 0,015
S	S	S<=0.008
S	S	S 0.015
S	S	S 0.03
S	S	0,008
S	S	S<0,008
nd	nd	S0,015
S	S	S0,015
nd	S	S<=0,008
S	S	0,03
S	S	S 0.25
S	S	S<=0,008
S	S	<=0,008
S	S	S0,008
S	S	S 0.015
S	S	S <= 0.008
S	S	0,015
S	S	S > 0.008
		S0,015
S	S	S 0.06
R	S	0,015
S	S	S<=0,008
S	S	S<=0,008
S	S	S0,015
S	S	S<=0.008
S	S	/
S	S	S0,015
R	S	0,03
nd	S	S0,06
S	S	S<0.008
S	S	S 0.25
S	S	S 0.06
S	S	S <= 0.008
nd	S	S0,12
S	S	S0,03
S	S	/

		/ 0.06
S	S	S 0.03
S	S	S<=0,008
S	S	S<=0,008
S	S	S0,008
S	S	0,008
S	S	S 0.03
S	S	S<=0,008
R	S	<=0,008
S	S	S<=0,008
nd	S	I 0.25
S	S	/ 0,25
S	S	S<=0,008
nd	S	I0,5
S	S	/
S	S	S 0.015
S	S	
S	S	S<= 0.008
S	S	S 0.015
S	S	S<=0,008
S	S	S0.015
		S<=0,008
S	S	S<= 0.008
S	S	S<= 0.008
S	S	S0,015
R	S	0,008
S	S	S0,03
S	S	S0,03
S	S	S<=0,008
S	S	
S	S	/ 0.5
S	S	S1
S	S	S<0,008
S	S	S0,03
nd	S	S 0.12
S	S	<=0,008
nd	S	S0,008
S	S	S<=0,008
S	S	S0,015
S	S	S0,015
S	S	S0,015
S	S	S 0.03
S	S	R > 8
S	S	S0,015
S	S	<=0,008
		0,12

S	S	S<0.008
S	S	S<= 0.008
S	S	S<= 0.008
S	S	/
S	S	
S	S	S 0.015
S	S	S<= 0.008
S	S	S0,015
S	S	S 0.008
S	S	S<=0.008
S	S	S 0.008
S	S	?
S	S	S<=0,008
	S	0,5
S	S	S<=0.008
**nd**	S	I0,25
S	S	S<=0,008
**nd**	S	S0,12
R	S	0,015
S	S	S<=0.008
S	S	/
S	S	S<= 0.08
S	S	S<=0,008
S	S	<=0,008
S	S	0,06
S	S	S <0.008
S	S	S0,03
S	S	
S	S	S 0.008
S	S	S<=0.008
S	S	<=0,008
R	S	0,015
S	S	R 0.25
S	S	S< 0.008
S	S	S<=0,008
S	S	
S	S	R1
S	S	S<=0.008
S	S	S<=0.008
S	S	S<=0,008
S	S	S<0.008
S	S	S<= 0.008
S	S	
S	S	/ 0,25
R	S	0,015
S	S	0,015
S	S	S<=0,008

S	S	S<=0.008
S	S	R>8
S	S	S<=0.008
nd	S	S 0.12
S	S	S0,03
S	S	.
S	S	S<= 0.008
S	S	S 0.015
S	S	S<=0,008
S	S	S<=0.008
S	S	S< 0.008
S	S	S<=0,008
S	S	
S	S	S<=0,008
S	S	S<= 0.008
S	S	S<=0.008
nd	S	I 0.25
S	S	S<=0,008
S	S	S< 0.008
S	S	S 0.015
S	S	S<= 0.008
nd	S	I0,25
S	S	S 0.015
S	S	S<= 0.008
		S<=0,008
S	S	0,015
S	S	0,015
nd	S	S 0.12
S	S	R8
S	S	S<=0,008
S	S	S <= 0.008
R	S	S0,03
R	S	0,015
S	S	S0,008
S	S	S 0.008
S	S	/0.06
S	S	S0,03
S	S	/0.5
S	S	
S	S	S<=0.008
nd	S	S 0.06
S	S	S <= 0.008
S	S	S0,03
S	S	
S	S	S 0.015
S	S	S <= 0.008
nd	S	S 0.06

nd	S	S 0.06
S	S	S0,015
R	S	0,06
R	S	S<= 0.008
S	S	S 0,008
S	S	S 0,008
S	S	0,015
S	S	S0,015
S	S	S0,008
nd	S	I0,25
R	S	S<=0,008
S	S	S<= 0.008
S	S	S0,03
S	S	S0,015
S	S	S<0.008
R	S	0,015
S	S	S<= 0.008
S	S	S<=0.008
S	S	S 0.03
S	S	R>8
S	S	S<= 0.008
S	S	S <= 0.008
S	S	S 0.015
S	S	S 0,015
S	S	S0,015
S	S	S0,03
S	S	S<=0.008
S	S	S 0.008
R	S	S<0,008
S	S	<=0,008
S	S	/ 0.25
S	S	S <= 0.008
S	S	S 0.015
S	S	S 0.015
S	S	S<=0.008
S	S	S0,008
S	S	S<=0,008
S	S	S<= 0.008
S	S	S<=0,008
		S<=0.008
S	S	S <= 0.008
S	S	S <= 0.008
		<=0,008
R	R	
R	S	0,015
R	S	0,015
R	S	0,015

S	S	
S	S	S<0.008
S	S	
S	S	S<=0,008
S	S	S<= 0.008
S	S	S0,03
S	S	<=0,008
		S0,015
S	S	S<=0.008
		S 0.015
S	S	S<=0,008
S	S	S0.03
S	S	S0,0015
R	S	S<= 0.008
S	S	S<=0,008
S	S	S<0,08
**nd**	S	S 0.06
S	S	S <= 0.008
S	S	S 0.015
S	S	S<=0.008
S	S	S 0.008
S	S	<=0,008
S	S	S<= 0.008
R	S	0,03
S	S	<=0,008
S	S	S<=0,008
S	S	S 0.008
S	S	S0,008
S	S	S0,015
S	S	S 0.015
S	S	S 0.015
**nd**	S	S 0.5
S	S	S<=0,008
S	S	
R	S	0,015
R	S	0,015
S	S	-
S	S	<=0,008
S	S	0,03
S	S	S<0.008
S	S	S<=0,008
S	S	I0,25
S	S	S<=0,008
S	S	S<=0,008
S	S	S<=0,008
**nd**	S	S 0.03
S	S	S 0.008

S	S	S<= 0.008
S	S	S0,015
S	S	S<= 0.008
S	S	<=0,008
nd	S	S0,12
nd	S	S0,12
S	S	S0,008
nd	S	S 0.06
S	S	<=0,008
R	R	
R	R	
S	S	S<= 0.008
S	S	S0,03
S	S	S0,015
S	S	S0,008
		<=0,008
nd	S	S 0.03

Susceptibility according to EUCAST (Voriconazole)	Susceptibility according to CLSI (Voriconazole)
**nd**	**nd**
**S**	**S**
**S**	**S**
**S**	**S**
**S**	**S**
**S**	**S**
**S**	**S**
S	S
**S**	**S**
**S**	**S**
**S**	**S**
S	S
S	S
**S**	**S**
S	S
**S**	**S**
**S**	**S**
S	S
**S**	**S**
**S**	**S**
**nd**	**nd**
**S**	**S**
**S**	**S**
**S**	**S**
**S**	**S**
**S**	**S**
S	S
S	S
**S**	**S**
**S**	**S**
R	R
**S**	**S**
**S**	**S**
**S**	**S**
**S**	**S**
**S**	**S**
S	S
**S**	**S**
**S**	**S**
**S**	**S**

S	S
S	S
nd	nd
S	S
S	S
S	S
S	S
S	S
S	S
S	S
S	S
S	S
S	S
S	S
S	S
S	S
S	S
nd	nd
S	S
S	S
S	S
nd	nd
S	S
S	S
S	S
S	S
S	S
S	S
S	S
S	S
S	S
S	S
S	S
S	S
S	S
S	S
S	S
S	S
S	S
S	S
S	S
S	S
S	S
S	S
S	S
S	S

nd	nd
S	S
S	S
S	S
S	S
R	S
nd	nd
S	S
S	S
S	S
S	S
S	S
S	S
S	S
nd	nd
S	S
S	S
S	S
S	S
S	S
S	S
S	S
S	S
S	S
nd	nd
S	S
nd	nd
S	S
S	S
S	S
S	S
S	S
S	S
S	S
S	S
S	S
S	S
S	S
S	S
S	S
S	S
S	S

	S
S	S
S	S
S	S
S	S
S	S
S	S
S	S
S	S
S	S
S	S
S	S
S	S
S	S
S	S
S	S
S	S
S	S
S	S
S	S
S	S
S	S
nd	nd
S	S
S	S
S	S
S	S
S	S
S	S
S	S
S	S
S	S
S	S
S	S
S	S
nd	nd
S	S
S	S
R	R
S	S

S	S
nd	nd
S	S
S	S
S	S
S	S
S	S
S	S
S	S
R	S
S	S
S	S
S	S
S	S
S	S
S	S
nd	nd
S	S
S	S
S	S
nd	nd
S	S
S	S
S	S
S	S
S	S
S	S
S	S
S	S
S	S
S	S
S	S
S	S
S	S
nd	nd
S	S
S	S
S	S
S	S
nd	nd
S	S
S	S
S	S
S	S
nd	nd

S	S
S	S
S	S
S	S
S	S
S	S
S	S
S	S
S	S
S	S
nd	nd
S	S
R	S
nd	nd
S	S
nd	nd
S	S
S	S
S	S
S	S
S	S
S	S
S	S
S	S
S	S
S	S
S	S
nd	nd
nd	nd
S	S
S	S
S	S
S	S
S	S
S	S
S	S
S	S
S	S
S	S
R	R
S	S
S	S

S	S
S	S
S	S
nd	nd
nd	nd
S	S
S	S
S	S
S	S
S	S
S	S
S	S
R	I
S	S
S	S
S	S
S	S
S	S
S	S
nd	nd
S	S
S	S
S	S
S	S
S	S
S	S
nd	nd
S	S
S	S
S	S
S	S
nd	nd
S	S
S	S
R	R
S	S
S	S
S	S
S	S
S	S
nd	nd
nd	nd
S	S
S	S
S	S

S	S
R	R
S	S
S	S
S	S
nd	nd
S	S
S	S
S	S
S	S
S	S
S	S
nd	nd
S	S
S	S
S	S
S	S
S	S
S	S
S	S
S	S
S	S
S	S
S	S
S	S
S	S
S	S
nd	nd
S	S
S	S
S	S
S	S
S	S
S	S
nd	nd
S	S
nd	nd
nd	nd
S	S
S	S
S	S
S	S
nd	nd
S	S
S	S
S	S

S	S
S	S
S	S
S	S
S	S
S	S
S	S
S	S
S	S
S	S
S	S
S	S
S	S
S	S
S	S
S	S
S	S
S	S
S	S
R	R
S	S
S	S
S	S
S	S
S	S
S	S
S	S
S	S
S	S
S	S
nd	nd
S	S
S	S
S	S
S	S
S	S
S	S
S	S
S	S
S	S
S	S
S	S
S	S
S	S

nd	nd
S	S
S	S
S	S
S	S
S	S
S	S
S	S
S	S
S	S
S	S
S	S
S	S
S	S
S	S
S	S
S	S
S	S
S	S
S	S
S	S
S	S
S	S
S	S
S	S
S	S
S	S
S	S
R	S
S	S
S	S
S	S
nd	nd
S	S
S	S
S	S
S	S
S	S
S	S
S	S
S	S
S	S
S	S

S	S
S	S
S	S
S	S
S	S
S	S
S	S
S	S
S	S
S	S
S	S
S	S
S	S
S	S
